# Supplementary material for: Towards a multilevel governance framework on the implementation of patient rights in health facilities: a protocol for a systematic scoping review
Source: BMJ Open. 2020 Oct 15;10(10):e038927. doi: 10.1136/bmjopen-2020-038927 (PMC7566736; doi:10.1136/bmjopen-2020-038927)
Supplement: Supplementary data [file bmjopen-2020-038927supp002.pdf]

Supplementary file 2

Detailed search strategy in the PubMed database

Table 1: Key concepts and related free text terms for search in the database

	Key concept 1	Key concept 2	Key concept 3
	**Patient rights**	**Patient rights instruments**	**Person centered care**
**Related free text terms**	Patient bill of rights, patient rights charter, quality of health care, social(community) accountability in health, Patient accountability, patient safety, discrimination, health equity, equality, right to health, patient rights and ethics	Patient rights legislation, patient bill of rights, health ombudsman, Right to information act, health councils, patient welfare committees, patient grievance redressal systems/ committees, patient complaints system, patient advocates, parliamentary hearings, public hearing, public protests, strategic litigation, consumer forums, professional associations, social audit, audit bodies, patient suggestion box, court system for patient rights, human rights commissions, health care quality councils	Patient centered care, people centered care, patient autonomy, patient engagement, Patient participation, Patient empowerment

The free text terms for each of the key concepts mentioned in Table 1 were pilot tested in the PubMed database. Each free text terms were searched separately to see whether the search results are relevant. The results of the initial search in the PubMed database are shown in Table 2. Since the initial search yielded unmanageable number of records, the search terms were made more specific. Also, use of Boolean operators such as “AND” and application of “free text filter” yielded relevant and manageable number of records. Since the governance aspect (not a core medical topic) of the patient rights implementation is the focus in the review, it was expected that the key concepts of the review may not be well indexed in the PubMed database. Hence, the search field included “All fields”. The refined search strategy is shown in Table 3.

Table 2: Results of the pilot testing of the free text terms in the PubMed database

**Free text terms**	**Search results**
Patient* bill of rights	529
Community accountability	8128
Patient safety	157872
Health equity	24519
Right to health	66755
Equality	367726
Patient rights and ethics	33598
Quality of health care	7025620
Social accountability	36577
Patient rights legislation	31773
Health ombudsman	321
Right to information act	1952
Health councils	117769
Patient welfare committees	1149
Patient grievance redressal systems	1
Patient complaint system	7540
Patient advocates	30901
Parliamentary hearings	11
Strategic litigation	1501
Consumer forums	1025
Professional associations	74651
Social audit	3558
Audit bodies	433
Patient suggestion box	13669
Court system for patient rights	301
Human rights commission	2252
Health care quality councils	97728
Patient centered care	33517
People centered care	14100
Patient autonomy	21190
Patient engagement	66813
Patient participation	55600
Patient empowerment	58745

Table 3: Refined search strategy in the PubMed database

Data source	Search terms	Search fields	Filters	Search results
PubMed database	Patient bill of rights	All fields	Full text	253
	Patient bill of rights AND implementation	All fields	Full text	23
	Patient charter of rights	All fields	Full text	147
	Patient charter of rights AND implementation	All fields	Full text	22
	Health ombudsman	All fields	Full text	186
	Patient grievance redressal	All fields	Full text	3
	Patient complaints AND Patient rights	All fields	Full text	270
	complaints procedures AND patient rights	All fields	Full text	173
	Rights AND strategic litigation	All fields	Full text	951
	Patient rights AND Consumer forums	All fields	Full text	14
	Consumer Health Forums AND patient rights	All fields	Full text	12
	Quality Health care AND patient bill of rights	All fields	Full text	80

Similar steps will be followed in the Web of Science and Lexis Nexis Database as well. The filters, search fields and the search terms will be chosen based on what is feasible in those databases.
