# Supplementary material for: Towards a multilevel governance framework on the implementation of patient rights in health facilities: a protocol for a systematic scoping review
Source: BMJ Open. 2020 Oct 15;10(10):e038927. doi: 10.1136/bmjopen-2020-038927 (PMC7566736; doi:10.1136/bmjopen-2020-038927)
Supplement: Supplementary data [file bmjopen-2020-038927supp003.pdf]

Quality appraisal criteria for the qualitative studies

Criteria	Study 1	Study 2	Study 3	Study …
Credibility				
Transferability				
Dependability				
Confirmability				
Overall quality	H/L/Judge			
Comments				

Note:

- To give more weight to studies that score high on quality
- Sensitivity analyses could be done to strike a balance between the methodological rigor and conceptual breadth and depth
