# Supplementary material for: Towards a multilevel governance framework on the implementation of patient rights in health facilities: a protocol for a systematic scoping review
Source: BMJ Open. 2020 Oct 15;10(10):e038927. doi: 10.1136/bmjopen-2020-038927 (PMC7566736; doi:10.1136/bmjopen-2020-038927)
Supplement: Supplementary data [file bmjopen-2020-038927supp005.pdf]

Supplementary file 5

Data extraction sheet

	Authors	Author affiliations	Funding for the study	Year of publication	Article type	Study design (for research papers)	Study setting	Country
Record 1								
Record 2								
Record 3								
….								
