# Supplementary material for: Towards a multilevel governance framework on the implementation of patient rights in health facilities: a protocol for a systematic scoping review
Source: BMJ Open. 2020 Oct 15;10(10):e038927. doi: 10.1136/bmjopen-2020-038927 (PMC7566736; doi:10.1136/bmjopen-2020-038927)
Supplement: Author's manuscript [file bmjopen-2020-038927.draft_revisions.pdf]

BMJ Open is committed to open peer review. As part of this commitment we make the peer review history of every article we publish publicly available.

When an article is published we post the peer reviewers' comments and the authors' responses online. We also post the versions of the paper that were used during peer review. These are the versions that the peer review comments apply to.

The versions of the paper that follow are the versions that were submitted during the peer review process. They are not the versions of record or the final published versions. They should not be cited or distributed as the published version of this manuscript.

BMJ Open is an open access journal and the full, final, typeset and author-corrected version of record of the manuscript is available on our site with no access controls, subscription charges or pay-per-view fees (http://bmjopen.bmj.com).

If you have any questions on BMJ Open's open peer review process please email info.bmjopen@bmj.com

BMJ Open

Towards a multi-level governance framework on the implementation of patient rights policies in health facilities:a protocol for a systematic scoping review

Journal:	*BMJ Open*
Manuscript ID	bmjopen-2020-038927
Article Type:	Protocol
Date Submitted by the Author:	29-Mar-2020
Complete List of Authors:	PUTTURAJ, MEENA; The University of Trans-disciplinary Health Sciences and Technology, Centre for local health traditions and policy<br>Van Belle, Sara; Department of Public Health., Institute of Tropical Medicine<br>Criel, Bart; Institute of Tropical Medicine, Department of Public Health<br>Engel, Nora; Maastricht University,<br>Krumeich, Anja; Maastricht University, Department of Health, Ethics and Society<br>B Nagendrappa, Prakash; The University of Trans-disciplinary Health Sciences and Technology, Centre for local health traditions and policy<br>Prashanth, NS; Institute of Public Health,
Keywords:	Health policy < HEALTH SERVICES ADMINISTRATION & MANAGEMENT, PUBLIC HEALTH, HEALTH SERVICES ADMINISTRATION & MANAGEMENT

SCHOLARONE™
Manuscripts

I, the Submitting Author has the right to grant and does grant on behalf of all authors of the Work (as defined in the below author licence), an exclusive licence and/or a non-exclusive licence for contributions from authors who are: i) UK Crown employees; ii) where BMJ has agreed a CC-BY licence shall apply, and/or iii) in accordance with the terms applicable for US Federal Government officers or employees acting as part of their official duties; on a worldwide, perpetual, irrevocable, royalty-free basis to BMJ Publishing Group Ltd ("BMJ") its licensees and where the relevant Journal is co-owned by BMJ to the co-owners of the Journal, to publish the Work in this journal and any other BMJ products and to exploit all rights, as set out in our licence.

The Submitting Author accepts and understands that any supply made under these terms is made by BMJ to the Submitting Author unless you are acting as an employee on behalf of your employer or a postgraduate student of an affiliated institution which is paying any applicable article publishing charge ("APC") for Open Access articles. Where the Submitting Author wishes to make the Work available on an Open Access basis (and intends to pay the relevant APC), the terms of reuse of such Open Access shall be governed by a Creative Commons licence – details of these licences and which Creative Commons licence will apply to this Work are set out in our licence referred to above.

Other than as permitted in any relevant BMJ Author's Self Archiving Policies, I confirm this Work has not been accepted for publication elsewhere, is not being considered for publication elsewhere and does not duplicate material already published. I confirm all authors consent to publication of this Work and authorise the granting of this licence.

For peer review only

Towards a multi-level governance framework on the implementation of patient rights policies in health facilities:a protocol for a systematic scoping review

1. Meena Putturaj *(Corresponding author)*
Centre for Local Health Traditions and Policy
The University of Trans -Disciplinary Health Sciences and Technology
74/2, Post Attur via Yelahanka, Jarakabande Kaval, Bengaluru, Karnataka 560064, India
meenaputturaj@gmail.com

2.Dr.Sara Van Belle
Department of Public Health,
Institute of Tropical Medicine, Antwerp
Belgium.
svanbelle@itg.be

3.Dr.Bart Criel
Department of Public Health,
Institute of Tropical Medicine, Antwerp
Belgium.
Bcriel@itg.be

4.Dr. Nora Engel
Department of Health Ethics and Society
Maastrict University
Netherlands
n.engel@maastrichtuniversity.nl

5. Dr. Anja Krumeich
Department of Health Ethics and Society
Maastricht University
Netherlands
a.krumeich@maastrichtuniversity.nl

6.Dr. Prakash BN
Centre for Local Health Traditions and Policy
The University of Trans -Disciplinary Health Sciences and Technology
Bengaluru, Karnataka, India
bn.prakash@tdu.edu.in

7.Dr. Prashanth Nuggehalli Srinivas
Institute of Public Health,
Bengaluru,Karnataka
India.
prashanthns@iphindia.org

Word count: 4685 excluding the title page, abstract, references, figures and tables

For peer review only

Towards a multi-level governance framework on the implementation of patient rights policies in health facilities:a protocol for a systematic scoping review

Abstract

Introduction

Patients rights are “those rights that are attributed to a person seeking health care”. Patients rights have implications for the quality in health care and acts as a key accountability tool. It can galvanize structural improvements in the health system and reinforces ethical health care. States are duty bound to respect, protect and promote patient rights. The rhetoric on patients’ rights is burgeoning across the globe. With changing modes of governance styles, a number of state and non-state actors and institutions at various levels play a role in the design and implementation of (patients rights’)policies. However, there is limited understanding on the implementation mechanisms for patient rights related policies of the state from a governance point of view.We attempt to fill this gap by critically analyzing the available scholarship on patient rights through a critical interpretive synthesis approach in a systematic scoping review.

Methods

The review question is “what works in terms of the state initiated implementation strategies and instruments to respect, protect and fulfill the patient rights in the health care facilities? How do they work at what level and in which contexts?”Three databases PubMed, Lexis Nexis (Academic) and Web of Science will be systematically searched for empirical and non-empirical literature in English from both LMIC and HIC. Targeted search will be performed in grey literature and through citation and reference tracking of key records. Using the critical

For peer review only - http://bmjopen.bmj.com/site/about/guidelines.xhtml

interpretive synthesis approach , a theoretical framework on the implementation of patient rights related policies in health facilities which is grounded in the data will be developed.

Ethics & Dissemination

The review uses published literature hence ethics approval is not required. The findings of the review will be published in peer reviewed journal.

Registration

PROSPERO registration is awaited.

Key words

Patient rights implementation, critical interpretive synthesis, multi-level governance

Strengths and limitations of this study

- The proposed review will deliver a explanatory framework on the patient rights implementation in health facilities based on the data/concepts retrieved from disciplines such as public health, social sciences,political sciences and law
- The multidisciplinary approach provides a nuanced understanding of the contested concepts such as the patient rights that complex involving many stakeholders and tradeoffs
- The Critical Interpretive Synthesis (CIS)approach to synthesize the evidence is a perfect fit to understand the nature of complex fuzzy concepts such as a patient rights implementation as it enables the use of data emerging from heterogenic disciplines and methodologies

For peer review only - http://bmjopen.bmj.com/site/about/guidelines.xhtml

- Further the CIS approach is a practical and a valid method for theory building
- Considering the time frame available for the review, the following are not within the scope of this review 1.Patient rights implementation by the informal health care providers ;2. Patients rights' in health research context for instance Clinical trials 3. Patient responsibilities/obligations

Introduction

Human rights are believed to be the rights and freedoms that are inherent to every person in this world without any bias. Formally in 1948, the aftermath of the World War II, the "UN Declaration of Human Rights" laid the foundation for defining fundamental rights shared by every human being (e.g. right to life, liberty and security, right to equality, freedom from torture etc.). These rights proclaimed in the Declaration were adopted by the UN General Assembly and ever since considered as the norm(s) by all nations for all people [1]. However, cultural relativists and post-colonial critics argue that the universal human rights doctrine in a multicultural society is an imposition of Western philosophy, values and norms on non-Western cultures[2]. Raimon Panikkar, a renowned Indian philosopher, argued that despite human rights being a Western notion, it can still act as an instrument for social justice in non-Western settings e.g. India. However, a cross-cultural philosophical approach to human rights is vital [3] and states have the obligation to respect, protect and fulfill human rights[1].

Embedded within the broader perspective of human rights is the notion of "patients rights"[4-6]. Patients rights' refer to "those rights attributed to a person seeking health care"[6]. It is the application of human rights principles in a health care setting - the location where health care providers offer services to the people.

For peer review only - http://bmjopen.bmj.com/site/about/guidelines.xhtml

Townend and his colleagues [8] **(see Figure 1)**identified five main categories of patient rights' 1) *individual rights* (certain liberties guaranteed for an individual person) 2) *consumer* (one who purchases the economic services and commodities) - *based rights* 3) *social rights* (emerging from social contract e.g. between state and the citizens) 4*) procedural patients rights* 5) *informational patients rights.* The last two categories (4 &5) are cross-cutting and actually help to enforce the first three categories of patient rights.

However, the categories mentioned above are not mutually exclusive. For example, the right to informed consent (individual right) is connected to right to information (procedural right). Likewise, the right to complain depends on the right to access medical files in order to prove that the right to informed consent was violated [8].

Why focus on patient rights'?

Because of structural inequalities such as power imbalances between the patient and the provider and other social inequities related to income gap,gender inequality, social class and stigma associated with health conditions e.g. HIV, tuberculosis, mental disorders etc health care settings are specifically vulnerable to patients rights' violations[9]. Most often, the vulnerability to patients rights violations emerges from a complex mixture of organizational and systemic factors (e.g. lack of human resources,for providing respectful care) shaping the health care provider-person encounter[10].There is considerable evidence regarding the prevalence of benevolent paternalism in the medical profession[11-15]. This has given rise to the scholarly work on people-centered care models emphasizing patient autonomy and joint decision-making [16].

The importance of patient rights' could be gleaned from multiple perspectives:

For peer review only - http://bmjopen.bmj.com/site/about/guidelines.xhtml

i) From the perspective of patient safety

In a traditional hierarchical relationship, patients might refrain from having an open communication with health care providers or the health care providers may not provide in-depth information to people seeking care . This lack of open communication might deter patients from participating in the discussions related to the prevention of health care related errors and thus have implications for patient safety. Patients rights' provide a language to empower and engage the patient in the processes of delivering health care[17].

ii) From the perspective of quality in health care

The six dimensions of quality health care are safety, timeliness/accessibility, patient centeredness/acceptability, equity, efficiency and effectiveness [18,19]. Most of the components of patient rights align with the key dimensions of quality health care. For instance, scholars have reiterated the need for respecting the patient rights as a tangible entry point for achieving people- centered care[20,21].Thus, the notion of patient rights' is in consonance with the concepts of quality of health care and people-centered care.

Iii) From the health system perspective

Furthermore, patient rights' might galvanize improvements at health system level by infusing accountability through oversight mechanisms [22]. Efficient and functional patient complaints and grievance redressal systems offer critical lessons for the structural improvement of the health system[23].Evidence from UK, New-Zealand and Canada show that the recommendations of patients rights' and safety ombudsmen have the potential to influence policy makers at the highest level [24-26].

iv) From an ethical perspective

For peer review only - http://bmjopen.bmj.com/site/about/guidelines.xhtml

Patient rights' are intertwined with the four core principles of medical/ health care ethics i.e. beneficence (do good), non - maleficence (do no harm), justice and autonomy [4]. Patients rights' and the principles of medical ethics complement each other. Any deviation of the health care professional's practice from medical ethics is often equated with the violation of patient rights'. See for instance, the Mid-Staffordshire National Health System (NHS) scandal in UK which revealed the preventable deaths of patients because of medical negligence by the health care staff[27], evoked concerns on the breach of patient's dignity and their right to quality care in the NHS[9].

v) From the health equity perspective

Research studies from both high-income contexts (e.g. USA, Europe)[28,29] and LMICs (e.g. India, Kyrgyzstan) [30-32] reveal the existence of discriminatory practices towards people receiving care. The discrimination could be based on single grounds (e.g. HIV status of a person, a migrant in Europe, sex worker etc.) or on the complex intersections of multiple grounds (e.g. age, gender, ethnicity, socio-economic status, disability etc.). The growing global consensus for respectful maternity care is a case in point that illustrates the magnitude of the patient rights violations experienced by the vulnerable populations (childbearing women) at all levels of health care[33]. It is acknowledged that strengthening accountability in the health system will help tackle health inequities[34]. Patient rights' could serve as a health system accountability tool [22,28].

Rationale

The social, cultural and the policy context shape the design and the implementation of patient rights' resulting in variations across settings [5].For example, on comparing the content of the patients' rights charter agreed by the European Union[35] with that of the

For peer review only - http://bmjopen.bmj.com/site/about/guidelines.xhtml

Indian[36] and the Ugandan patients bill of rights[37], the European Union patients' rights charter had included "right to innovation" "right to avoid unnecessary suffering and pain" "right to personalized treatment" "right to respect patients time". These rights were not mentioned in the Indian and the Ugandan bills of patients rights'. The levers to enforce the patient rights also vary between countries. In countries like UK[38], Netherlands[7], Sweden[7], Canada[39] patient rights are enshrined in law and there are separate statutory bodies, health ombudsmen (i.e. an independent public authority who deals with citizens' complaints against the public/private institutions) are available to people who wish to complain when they feel that their rights are not respected. The National Health System of UK also provides assistance to the patients to navigate the grievance redressal system[38]. Inadequate institutional mechanisms to respect, protect and fulfill human (patients) rights in certain Low and Middle -income countries (LMIC) (e.g. India, South Africa, Brazil) are increasingly compensated by citizen action and strategic litigation steered by civil society organizations[22,40,41].

Patient rights' movements and civil society organizations arguing for improved accountability in health care are gathering momentum across the globe and more so in LMICs [40]. Although the rhetoric on patients rights' is burgeoning, empirical research in this area is nascent and most of the existing research studies [42-45] limit themselves to studying awareness of patients rights'. Also, the available frameworks in the literature identify the typology of patients rights'[7] and a few other attempt to situate the patients rights' within the larger human rights framework described above[4].According to the human rights law, states are duty-bound to protect human rights. States have the obligation to establish appropriate policies and ensure its implementation concerning the protection of human rights vis-a-vis patient rights[1]. In general, the importance of

For peer review only - http://bmjopen.bmj.com/site/about/guidelines.xhtml

governance for effective design and implementation of state policies is imperative. With changing modes of governance styles from top down hierarchical to more collaborative forms, a number of state and non-state actors (e.g. professional associations, civil society organizations) and institutions (such as judiciary, media) at various levels play a role in the design and implementation of (patients rights') policies. However, there is limited understanding on the governance related linkages especially with the health policy processes. The knowledge is even more limited when it comes to the implementation mechanisms for patient rights related policies of the state.

We attempt to fill this gap by undertaking a systematic scoping review by giving special emphasis on the governance/institutional arrangements (formal and informal) procedures and processes at different levels (state, district, subdistrict and the health facility level) pertaining to the patient rights implementation .In the field of evidence synthesis, systematic scoping reviews are considered as an appropriate approach to answer complex questions where systematic reviews are not possible, or do not produce the required (conceptually rich) information. Unlike the systematic reviews, systematic scoping reviews are applied for investigating broad questions that are interdisciplinary in nature but in a systematic manner and does not involve strict exclusion criteria for the studies [50].Previously several scholars have provided frameworks in an effort to standardize the conduct and the reporting of scoping reviews. The proposed review uses the most recent PRISMA guidelines[50] published in 2018 for the systematic scoping reviews

Further in this systematic scoping review, considering the limited theoretical development in the area of patient rights implementation, the critical interpretive synthesis(CIS) [51] would be a used as a technique for searching and synthesizing the evidence. The CIS

For peer review only - http://bmjopen.bmj.com/site/about/guidelines.xhtml

approach offers guidance to marshall evidence to answer complex fuzzy questions that often cut across multiple disciplines as it allows to

- use diverse body of evidence from varied forms of data sources,disciplines and methodologies
- modify the review question in response to the search results and findings of the previously retrieved items
- use literature from the adjacent fields relevant to the emerging nature of the review question
- use a pragmatic approach to identify potentially relevant and conceptually sound literature that would contribute for theory building or an explanatory framework
- critique the ways of the problematization of the phenomena under study in the literature and explore the underlying assumptions on which the solution to the social problems are constituted

For peer review only

For peer review only - http://bmjopen.bmj.com/site/about/guidelines.xhtml

CIS is successfully applied in the field of health systems to explore areas such as informal payments in maternal health care[52], access to health care[51], overuse of health services[53]. The concept of patient rights itself is a subject of multiple interpretations, partially owing to the confusion that exists on who is claiming these rights - is it the patient or consumer or citizen?[54].The interrelatedness of patient rights with health system accountability, quality of health care, health care ethics, and equity issues further makes it difficult to define clearly the patient rights conceptually. Further

Salient features of the CIS approach [51]

- The review question serves as a compass rather than an anchor and is therefore revised and refined during the review process
- The aim is to develop synthesizing argument which could take the form of a plausible theoretical framework reflecting the network of constructs grounded in the evidence
- The inductive and iterative nature of the CIS approach demands several cycles of searching, sampling, critiquing and analyzing the evidence until a theoretical saturation is reached
- The subsequent cycles of searching, sampling, critiquing and analyzing the evidence is informed by the emerging theoretical framework
- The priority for selecting the evidence is “signal”(i.e.likely relevance to answer the review question) over “noise”(the inverse of methodological quality). However, formal quality appraisal techniques could be applied for individual papers. The aim is to purposively select papers that are most relevant and conceptually rich
- Data extraction charts initially may help in the review but not an essential feature of the CIS approach
- The review may not not be strictly reproducible, auditable. It can be argued that even with primary data in a qualitative study or other evidence synthesizing techniques it is most likely that a different set of researchers may interpret the same data/ set of evidence and come up with the different theoretical model.
- The synthesizing argument “theoretical framework” is a product of interpretive process and“ authorial voice” .Therefore there is a need for constant reflexivity throughout the review process on the part of the authors.

attempting to explore the ” implementation” aspect of the patient rights adds another layer in the complexity owing to its overlapping with the related fields such as governance and health policy processes. Since the focus of the CIS approach is towards achieving conceptual clarity and theory development, it might help to search , retrieve

For peer review only - http://bmjopen.bmj.com/site/about/guidelines.xhtml

and synthesize from the literature on patient rights implementation that is diverse and heterogenic in terms of methods and disciplines.

The* initial *review question

What works in terms of the state initiated implementation strategies and instruments to respect, protect and fulfill the patient rights in the health care facilities? How do they work at what level and in which contexts?

Objectives

The objectives of this review are two fold

1)To describe the various strategies, approaches and instruments for patient rights implementation in the health facilities 2)To propose a theoretical framework on the implementation of patients' rights policies in the health facilities from a multi-level governance perspective

Methods

Operational definitions of the key terminologies used in the review

Patients rights- In this review the scope of patient rights covers the patient rights applicable for facility-based preventive, promotive , curative and rehabilitative health care services offered in the primary, secondary and tertiary level health care settings

Patients rights' implementation strategy refers to the overall strategic approach and the plan of action taken by the state and/or the various non-state actors such as communities, non-governmental organizations, civil society organizations and professional bodies to respect, protect and fulfill the patients rights'.

For peer review only - http://bmjopen.bmj.com/site/about/guidelines.xhtml

Patients rights' implementation instruments refers to the set of procedures, methods and tools available to implement patient rights. Examples include patient grievance redressal committees; patient rights charter; ombudsman; public hearing; parliamentary hearing; strategic litigation by NGO(s); public protests & patient directly filing a petition in the court system.

Patients rights' implementation mechanisms refers to the means and the ways through which a particular patient rights enforcement strategy/ Instrument is expected to work in a given context.

Multi-level governance refers to the distribution of governance across mutilple jurisdictional levels (national, subnational, local etc) in a polity.These jurisdictions can be designed around a particular community/territory or a policy problem or based on a specific functionality [55].

The initial conceptual framework to guide the evidence synthesis

The initial conceptual framework **(see Figure 2)** is developed from the concepts derived from the initial peer reviewed empirical literature on patients rights'.We also borrowed conceptual elements for this framework from the public policy implementation models of Grindle and Thomas[56], Mazmanian and Sabatier[57] , Hill and Hupe [58] and approaches such as multi-level governance[59], policy coherence[60] and what the problem represented to be (WPR approach) developed by Carol Bacchi [61]. This starter framework will be further refined using the CIS technique in this systematic scoping review.

Basic assumptions in the framework

For peer review only - http://bmjopen.bmj.com/site/about/guidelines.xhtml

1) Respecting, protecting and fulfilling the patient rights at health facility level depends on the design and implementation of patients rights' strategies through state policies.[71]

2) The engagement of the actors and the institutions in the patient rights related policy processes depend on a number of factors like the actors'/institutions formal (e.g. position in the administration), informal source of power (e.g. social status) and their interest in patients' rights issues [55,56].

3) The design and the implementation of the policy are interdependent on each other[55].

Patients rights' policy design

Policies designed at the national/provincial level should be clear, consistent, adequate and coherent to guide the policy implementers at the lower levels (district-sub-district-health facilities). If the patients rights' implementation provisions are scattered in multiple policies, then policy coherence is key to have a synergistic effect [59].

Approaches, instruments and procedures for implementation

Successful implementation depends on the commitment, discretion and disposition of the state actors belonging to the implementing agency(ies) and structures. If there is more than one implementing institution involved, and/or if the processes include different levels because of political and administrative decentralization, then effective coordination between the levels is crucial. Coordination depends on the number of steps and procedures required to achieve patient rights policy objectives. Conflicting positions and interests of the implementing agency/actors with the policy objectives is likely to affect the implementation processes in a negative way [56,59].

Resources required for design and implementation of patient rights

For peer review only - http://bmjopen.bmj.com/site/about/guidelines.xhtml

For peer review only

Political, financial, managerial and technical competencies of the actors/ institutions at the national and the subnational levels define the effectiveness of policy instruments used to enforce the patients rights' policies/strategies*.* *Political resources* entail the availability of political and/or public support for policy design and implementation. Public engagement in the policy processes concerning patient rights is key. Assessing and mobilizing political resources depends on the knowledge and willingness of the policy makers and the implementers. *Financial resources* refer to the funds that are allocated for facilitating patient rights' policies implementation activities (e.g. recruiting manpower, logistics, infrastructure, equipment, supplies, training, etc.). *Managerial resources* indicate the availability of tools monitoring mechanisms within the patients rights' enforcement system (e.g. reporting, recording, supervision of the enforcing officials). *Technical resources* denote the existence of technical capacity and assistance for policy implementation e.g. use of information and communication technology. All of these resources will influence the accessibility and availability of the patient rights' policies in the manner comprehensible to the stakeholders, including citizens (and the most vulnerable groups) [56].

Framing and competing perspectives on patient rights

Patient rights are within a contentious policy area. Therefore implementation of patient rights should not be seen as only a matter of clear policy design and matching resources for implementation. The policy documents related to patient rights (e.g. Patient rights charter) , implementation strategies and instruments are a product of a certain context and time and hence have been framed in certain discourses. Understanding how the issues on patient rights are constituted, represented, given shape and meaning within the policies is cruial

For peer review only - http://bmjopen.bmj.com/site/about/guidelines.xhtml

[61]as it determines the way the implementation is finally panned out within the multiple tiers of the polity.

Context [58]

Strategies and the policies designed to respect, protect and fulfill the patients rights' are embedded within a specific social,political,cultural,economic, legal and organizational context. The contextual conditions at the i) micro (facility and the individual patient provider) level e.g. Nature of the patient provider relationship, meso (district and subdistrict) level e.g. organizational culture of the implementation bodies and macro level (national and subnational) e.g. economic policies, reforms in health professional education and broader societal and the cultural issues (e.g. social exclusion and discrimination based on gender, class etc will influence the implementation processes. Further, it will be an easier task to implement patient rights, when the players supposed to practice them have somehow 'internalized' the notion and the importance of patients rights'[62]. In that respect that there should be room in other policies that have implications for patient rights' implementation e.g. Adapting the training curricula of health care providers, health systems managers, public health professionals etc.

Multiple potential outcomes [55]

The complex interaction of the actors/institutions, the implementation instruments and the policy context at various levels and broader societal context will determine the actual implementation practices related to patients rights' at the health facility level. Again the patient rights implementation practices could vary based on the nature of the ownership and the mission statement of the health care institution for which it exists. For instance,

For peer review only - http://bmjopen.bmj.com/site/about/guidelines.xhtml

For peer review only

there could be a private health care institution with a public goal or a public health care institution with a profit goal for sustainability reasons [63].

When the patients rights' policies are implemented effectively and conducive context conditions are in place , this should result in enhanced accountability and equity in the health system [23,29,30]. Further, at the health facility level, this is expected to lead to improved quality of care[18,21,22] and to promote a culture of respecting and protecting the patients rights'[4]. In case of ineffective implementation of patients rights' policies/strategies, there will be more discriminatory practices in the health facilities, contributing to the denial of patients rights', widening health inequities and poor accountability in the health system[24,29-33].Also, it is possible that in settings where there is poor response of the state to the patient rights violations, people might feel frustrated and resort to violence against the health care providers.

Eligibility criteria

Data sources

Search strategy:there are three main sources for this review 1) select databases 2) grey literature 3) reference tracking and citation tracking. To get a comprehensive understanding on patients rights implementation strategies and instruments from a multidisciplinary perspective, three databases PubMed (health literature), Web of Science (social sciences), Lexis Nexis Academic (Law) will be searched in a systematic manner for all types of evidence (both empirical and non-empirical) broadly on patient rights', patients rights' implementation in the health facilities and patients rights' approaches/strategies/ mechanisms/instruments. The search terms will be decided based on what terms are feasible to be used in the respective data base. Boolean operators will be used to develop

For peer review only - http://bmjopen.bmj.com/site/about/guidelines.xhtml

various combinations of search terms feasible for usage in the database selected for the review. Initially, PubMed will be searched to identify the alternative terms/index terms for the key search terms for the review. Followed by PubMed, a more detailed search will be undertaken in the Web of Sciences and Lexis Nexis databases. We have provided our detailed search strategy for each of the three databases in the supplementary file 1. Further, targeted searches will be performed in grey literature 1) Google 2) Websites of the institutions (public and private) that are relevant to the area of patient rights, social justice, human rights and accountability in health systems (e.g. People's Health Movement , Health Care Quality Accreditation agencies, other civil society institutions(national and international), Patient rights organizations (national and international) e.g. Coalition patient rights, National Patient Advocate Organization etc. Also, the reference list of the specific papers(reference tracking) and the papers that cite the key papers (citation tracking) will be hand searched to identify any additional studies that are conceptually relevant and have the potential to contribute for the emerging framework.

Table 1:Key search domains and the related terms to be used in different permutations and combinations in the selected databases

Key search domains	Related terms
Patient rights	Patient's bill of rights OR patient charter OR patient engagement ,quality of health care, person/ patient centered care, social(community) accountability in health,Patient accountability, participation, patient safety, discrimination, health

For peer review only - http://bmjopen.bmj.com/site/about/guidelines.xhtml

	equity, equality, right to health, patient rights and ethics
Patient rights instruments	Patient rights legislation, patients charter, patient bill of rights, health ombudsman, Right to information act, health councils, patient welfare committees, patient grievance redressal systems/ committees, patient complaints system, patient advocates, parliamentary hearings, public hearing, public protests, strategic litigation, patient advocacy groups, consumer forums, professional associations, social audit, audit bodies, patient suggestion box, court system for patient rights, human rights commissions, health care quality councils
Person centered care	Patient centered care, people centerd care, patient autonomy

Note: The search terms mentioned in the table are only indicative and not exhaustive. The list will be further enriched after the initial search for the studies in the PubMed database

Period of search:the aim of the literature search is to maximise the possibility of identifying the conceptually rich articles to enable theory building hence no specific time period is stipulated for this review.

Publication type:as indicated in the state of the art literature on patient rights, it is clear that the available scholarship is mostly from the European contexts.It is expected that there

For peer review only - http://bmjopen.bmj.com/site/about/guidelines.xhtml

will be limited empirical work on the patuent rights in the LMICs.In order to increase the transferability of the the theoretical framework even to the the LMIC settings it was decided that all types of evidence (both empirical and non empirical) in English from both LMIC and HIC will be included in this review

Time period scheduled for the review: The data collection will begin on 15/04/2020. The review will get completed by August 15/04/2020.

Article selection process (see Figure 3)

1. Systematic search for the articles on patient rights implementation in the selected three data bases
2. Identifying the relevant articles in grey literature and through citation and reference tracking
3. Title and abstract review of the articles to identify the relevant articles that are concordant with the initial review question
4. Creating a sampling frame of all articles
5. Purposive sampling of the articles from the sampling frame that point towards relevancy and conceptual soundness
6. Applying the quality appraisal criteria for the individual papers. Perform sensitivity analysis to strike a balance between methodological rigour and conceptual breadth and depth of the study. To give more weightage to the articles that score high on quality
7. Initial data extraction on the study characteristics
8. Concept mapping in the retrived literature and identification of the conceptual gaps

For peer review only - http://bmjopen.bmj.com/site/about/guidelines.xhtml

9. Repeating the search in the selected databases,grey literature, citation tacking and refernce tracking to fill the conceptual gaps until a theoretical saturation is reached and refining the synthesizing argument i.e.“Theoretical framework”on the patient rights implementation

Quality appraisal criteria:since the review uses articles emerging from atleast three diverse disciplines (public health, social sciences and law), finding a common quality assessment framework is challenging. However, it is expected that majority of the empirical work will be of qualitative in nature. Hence, the quality of the studies will be evaluated using the Hannes criteria (2011) [64] for assessing qualitative research (see supplementary file 2). The main parameters for evaluation would be 1) credibility 2)transferability 3) dependability 4) Confirmability. The quality of the study will be determined using a three point likert scale(strong moderate and weak). For quantitative studies, quality appraisal tool developed by Effective Public Health Practice Project[65] will be used (see supplementary file 3)

Data extraction:data will be extracted based on the review question and the emerging conceptual framework developed (see supplementary file 4).The main categories that will be charted include 1)author(s) 2)year of publication 3) no of times the article citation 4) funding source 5)setting 6) type of the paper 7) type of patient rights implementation strategy 8) type of patient rights implementation tool/instrument 9) description of the tool 10) patient rights implementation mechanism (how the tool is expected to work) 11)level at which the patient rights instrument is operationalized purpose (why) of the strategy/tool 12) Framing on the patient rights issue 13) context (macro, meso and micro) in which the strategy or the tool is operationalized 14)lessons learned actors involved in the patient rights implementation strategy 15) resources required to implement the strategy/ tool16)

For peer review only - http://bmjopen.bmj.com/site/about/guidelines.xhtml

reported outcomes 17)equity effects. New categories would be added or the existing categories will be revised in response to the evolving data collection and analysis.The first investigator will extract the data and this will be independently checked atleast by two other senior researchers of the review team. Further the four authors MP, SVB, NE and PNS will deliberate on the data collection and the data analysis at regular intervals within the stipulated time for the review.

Patient and Public involvement

This systematic scoping review is part of the 4-year PhD research project of MP. The framework developed from this review will be further empirically tested in a LMIC setting (India) during the the second phase of the PhD using comparative case study design. Further, in the third phase of the project stakeholder consultation workshop will be held to validate the theoretical framework and the policy recommendations.

Synthesis of results

The CIS approach used in the evidence synthesis is inductive and iterative in nature. The retrieved items/papers will be critically analysed forpatterns and themes that explain the phenomena of "patient rights implementation" in the literature. By constant comparison of the theoretical constructs with the data in the papers, the relationships between the theoretical constructs will be identified.The conceptual gaps identified will be further filled with subsequent search and analysis of the literature.The aim is to be critical while synthesizing the evidence by questioning 1) the ways in which the patient rights has been problematized or represented to be in the literature 2) the underlying assumptions behind the policy options or the implementation strategies for patient rights.The synthesis of the evidence retrieved in this review would thus result in a unifying framework (the synthesizing

For peer review only - http://bmjopen.bmj.com/site/about/guidelines.xhtml

argument) on the implementation of patient rights policies from a multi-level governance perspective that is grounded in the data.

Conclusion

In general, there is limited scholarly work on patient rights implementation from a multi-level governance perspective and the available frameworks on patient rights explain the nature and the typology of patient rights,[7] few others attempt to situate the patient rights' in the larger human rights framework [4]but the proposed systematic scoping review with the help of the CIS approach will deliver a framework – an explanatory model on the patient rights implementation. The framework will contribute to the knowledge domain of implementation of contested policies in the domain of patient rights. The theoretical framework will be grounded in the data from the literature belonging to several disciplines (public health, social sciences/political science and law). A multidisciplinary approach provides a nuanced understanding of the patient rights policies that are complex involving many stakeholders and tradeoffs. The framework will also inform the practice and policy decisions concerning patient rights.

Ethics and dissemination

Ethical approval will not be required because this study will retrieve and synthesise data from already published literature.The findings of this review will be published in peer reviewed journal.

References

For peer review only - http://bmjopen.bmj.com/site/about/guidelines.xhtml

For peer review only

1. United Nations. Universal Declaration of Human Rights. General Assembly resolution 217 A. Newyork: United Nations;1948. Available from: https://www.un.org/en/universal-declaration-human-rights/. Accessed 13 Jan 2020.
2. Imani O N. "Critical Impairments to Globalizing the Western Human Rights Discourse."Societies Without Borders.2008; 3 (2): 270-284.Available at:https://scholarlycommons.law.case.edu/swb/vol3/iss2/. Accessed 13 Jan 2020.
3. Panikkar R. Is the notion of Human rights a western concept? Diogenes 120; 1982.Avaliable at:http://www.derechoshumanos.unlp.edu.ar/assets/files/documentos/is-the-notion-of-human-rights-a-western-concept.pdf.Accessed 15 Jan 2020.
4. Peled-Raz M. Human rights in patient care and public health-a common ground. Public Health Rev. 2017; 38:29.
5. Cohen J, Ezer T. Human rights in patient care: a theoretical and practical framework. Health Hum Rights. 2013 Dec 12;15(2):7-19.
6. World Health organization. Patient Rights. Geneva: World Health Organization;2019. Available at: https://www.who.int/genomics/public/patientrights/en/ . Accessed 20 Jan 2020.
7. The free dictionary by Farlex.Patient rights. Pennyslvania:Farlex, Inc;2003-2019.Available from: https://medical-dictionary.thefreedictionary.com/patient+rights.Accessed 02 Jan 2020.
8. Townand D, Clemens T, Shaw D, Brand H, Nys H & Palm W.Patient's rights in the European Union, A mapping exercise. Luxembourg: European Union; 2016.

For peer review only

For peer review only - http://bmjopen.bmj.com/site/about/guidelines.xhtml

9. Méndez J. Report of the Special Rapporteur on torture and other cruel, inhuman or degrading treatment or punishment. New York: United Nations General Assembly;2013.

10. Reader WT & Gillespie A. Patient neglect in health care institutions: A systematic review and conceptual model. BMC Health Ser Res.2013;13:156.

11. Bell SK, Roche SD, Mueller A, et al Speaking up about care concerns in the ICU: patient and family experiences, attitudes and perceived barriers. BMJ Qual Saf. 2018; 27:928-936.

12. Tong WT, Lee YK, Ng CJ, Lee PY. Factors influencing implementation of a patient decision aid in a developing country: an exploratory study. Implement Sci. 2017 ;12(1):40.

13. Cubaka VK, Schriver M, Kayitare JB, Cotton P, Maindal HT, Nyirazinyoye L & Kallestrup P. 'He should feel your pain': Patient insights on patient-provider communication in Rwanda. Afr J Prim Health Care Fam Med. 2018:10(1).

14. Fochsen, G, Deshpande, K, & Thorson, A. (2006). Power Imbalance and Consumerism in the Doctor-Patient Relationship: Health Care Providers' Experiences of Patient Encounters in a Rural District in India. Qualitative Health Research, 16(9), 1236–51.

15. Henderson S. Power imbalance between nurses and patients: a potential inhibitor of partnership in care. J Clin Nurs. 2003 Jul;12(4):501-8.

16. Mead N, Bower P. Patient-centredness: a conceptual framework and review of the empirical literature. Soc Sci Med. 2000 Oct;51(7):1087-110.

17. Virona Gabriella M & Tarasenka N. Patient safety and rights: developing tools to support consumer health literacy. Regional Office for Europe. World Health Organization;2010.

For peer review only - http://bmjopen.bmj.com/site/about/guidelines.xhtml

18. Institute of Medicine. Crossing the Quality Chasm: A New Health System for the 21st Century. Washington DC: National Academy Press; 2001.

19. World Health Organization. Quality of care:A process for making strategic choices in health systems.Geneva:World Health Organization;2006.

20. Groene O. Patient centeredness and quality improvement efforts in hospitals: rationale, measurement, implementation. Int J Qual Health Care. 2011; 23(5): 531–37.

21. World Health Organization & UNICEF. Declaration of Asthana. Global conference on primary Health Care;2018. Available from: https://www.who.int/primary-health/conference-phc/declaration. Accessed Feb 20 2020.

22. Van Belle S, Boydell V, George AS, Brinkerhoff DW, Khosla R. Broadening understanding of accountability ecosystems in sexual and reproductive health and rights: A systematic review. PLoS One. 2018 May 31;13(5):e0196788.

23. Mirzoev T &Kane S.Key strategies to improve systems for managing patient complaints within health facilities- What can we learn from the existing literature?. Glob Health Action.2018;11:1458938.

24. Parliamentary and Health Service Ombudsman Impact study presentation of findings. London (United Kingdom): The Ombudsman;2010. Available at: www.ombudsman.org.uk/__data/assets/pdf_file/0013/4144/Impact-Study.pdf. Accessed Jan 02 2020.

25. Paterson R.The patients complaint system in Newzeland.Health Aff.2002;21(3):70-9.

26. Annual report 2009–2010. The Ombudsman: Quebec; 2010. Available at: www.protecteurducitoyen.qc.ca/en/cases-and-documentation/index.html. Accessed Feb 02 2020.

For peer review only - http://bmjopen.bmj.com/site/about/guidelines.xhtml

27. Francis R. Independent Inquiry into care provided by Mid Staffordshire NHS Foundation Trust January 2005 – March 2009. London: The Stationery Office;2010.

28. European Union Agency for fundamental rights. Inequalities and multiple discrimination in access to and quality health care.Luxembourg:European Union Agency for Fundamental Rights; 2013.

29. Glover ML, Sim M & Winters K. Perceived discrimination and reported trust and satisfaction with providers in African Americans. The Jackson heart study. Ethnicity & Disease.2017;27(3): 209-15.

30. Goerge S. Caste and Care: Is Indian Healthcare delivery system favourable for Dalits?Bangalore:The Institute of Social and Economic Change;2015.

31. Irudayam, A, J P Mangubhai & J G Lee. Dalit Women Speak Out: Violence against Dalit Women in India, Volume I and II. New Delhi: National Council for Dalit Human Rights;2006.

32. Open Society Foundations. Reports on the Patient Rights Violations in Krygystan. Newyork: Open Society Foundations ;2008.

33. Bowser D & Hill K.Exploring evidence for disrespect and abuse in facility based childbirth.Report of a landscape analysis.Washington DC: USAID;2010.

34. Hammonds R, Hanefeld J & Ooms G. Accountablity as a driver of heath equity.Denmark: WHO Regional Office for Europe;2019.

35. Open Society Foundations. International/European framework for protection of human rights in patient care. Public Health Fact Sheet.Newyork: Open Society Foundations;2013.Available at: https://www.opensocietyfoundations.org/publications/international-european-framework-protection-human-rights-patient-care. Accessed Feb 19 2020.

For peer review only - http://bmjopen.bmj.com/site/about/guidelines.xhtml

36. National Human Rights Commission of India. Draft of patient rights charter. NewDelhi: Ministry of Health and Family Welfare;2018.Available at: https://mohfw.gov.in/newshighlights/draft-patient-charter-prepared-national-human-rights-commission.Accessed Feb 08 2020.

37. Department of Quality Assurance. Ministry of Health. Republic of Uganda. Patient's Charter:Uganda; Republic of Uganda;2009.Available at: http://cphl.go.ug/sites/default/files/library resources/PATIENTS%27%20CHARTER.pdf. Accessed Jan 22 2020.

38. Boudioni M, McLaren S & Lister G. A critical analysis of national policies, systems, and structures of patient empowerment in England and Greece. Patient Prefer Adherence. 2017; 11:1657–69.

39. Ontario Ministry of Health Care. Patients first :Action Plan for health care. Ontario: Ministry of long term care;2008. Available at: http://www.health.gov.on.ca/en/ms/ecfa/healthy_change/faqs_patientombudsman.aspx. Accessed Jan 03 2020

40. Shukla A, More A & Marathe S. Making private health care accountable:Mobilizing civil society and ethical doctors in India.Institute of Development Studies Bulletin.2018;49(2): 129-46.

41. Open Society Foundations.Strategic litigation impact:Insights from global experience.Newyork:Open Society Foundation;2018.

42. Unnikrishnan B, Trivedi D, Kanchan T, Rekha T, Mithra P, Kumar N, Kulkarni V, Holla R &Talish M. Patients' Awareness About Their Rights: A Study from Coastal South India. Sci Eng Ethics. 2017 Feb;23(1):203-14.

For peer review only - http://bmjopen.bmj.com/site/about/guidelines.xhtml

43. Agrawal U, D'Souza BC & Seetharam AM. Awareness of Patients' Rights among patients of a Tertiary Care Teaching Hospital- A Cross-sectional Study. J Clin Diagn Res. 2017 ;11(9): IC01-IC06.

44. Kagoya HR, Kibuule D, Mitonga-Kabwebwe H, Ekirapa-Kiracho E, Ssempebwa JC. Awareness of, responsiveness to and practice of patients' rights at Uganda's national referral hospital. Afr J Prim Health Care Fam Med. 2013;5(1):491. doi:10.4102/phcfm.v5i1.491

45. Abedi G, Shojaee J, Moosazadeh M, et al. Awareness and Observance of Patient Rights from the Perspective of Iranian Patients: A Systematic Review and Meta-Analysis. Iran J Med Sci. 2017;42(3):227–34.

46. Brinkerhoff DW, Jacobstein D, Kanthor J, Rajan D & Shepard. Accountability, health governance and health systems: uncovering the linkages. Washington DC: USAID;2017.

47. Munn Z, Peters DJM, Stern C, Tufanaru C, McArthur A & Aromataris E. Systematic review or scoping review? Gudance for authors when choosing between a systematic or scoping review approach. BMC Med Res Methodol.2018;18:143.

48. Schaink AK, Kuluski K, Lyons RF, Fortin M, Jadad AR, Upshur R, Wodchis WP. A scoping review and thematic classification of patient complexity: offering a unifying framework. J Comorb. 2012 Oct 10;2:1-9. eCollection 2012. Review. PubMed PMID: 29090137; PubMed Central PMCID: PMC5556402.

49. Harfield, S.G., Davy, C., McArthur, A. et al. Characteristics of Indigenous primary health care service delivery models: a systematic scoping review. Global Health.2018;14(12). doi:10.1186/s12992-018-0332-2

For peer review only

50. Tricco, AC, Lillie, E, Zarin, W, O'Brien, KK, Colquhoun, H, Levac, D, Moher, D, Peters, MD, Horsley, T, Weeks, L, Hempel, S et al. PRISMA extension for scoping reviews (PRISMA-ScR): checklist and explanation. Ann Intern Med. 2018;169(7):467-73.

51. Dixon-Woods M, Cavers D, Agarwal S, Annandale E,Arthur A, Harvey J et al. Conducting a critical interpretive synthesis of the literature on access to health care by vulnerable groups. BMC Med Res Methodol.2006;6:35.

52. Schaaf M & Topp MS. A critical interpretive synthesis of informal payments in maternal health care.Health Policy Plan.2019;34:216-29.

53. Wilson GM, Ellen EM, Lavis NJ, Grimsahw MJ, Moa A K,Shemer J et al. Processes, contexts and rationale for disinvestment: a protocol for a critical interpretive synthesis.Syst Rev.2014;3:143.

54. Mold A. Patient's rights & the National Health Service in Britain1960s-1098s. Am J Public Health.2012;102(11): 2030-37.

55. Hooghe L &Marks G.Unravelling the central state, but how?Types of Multilevel governance.Am Polit Sci Rev.2003;97(2):233-43.

56. Grindle SM &Thomas WJ. Public choices and policy change. The political economy of reform in developing countries. Baltimore: John Hopkins University Press; 1991.

57. Sabatier P, Mazmanian D. The implementation of regulatory policy: A framework of analysis. Davis: Institute of Governmental Affairs; 1979.

58. Hill M, Hupe P. In: Aguilera , editor. Implementing Public Policy. 3rd ed. London: Sage Publications; 2014.

59. Peters BG & Pierre J. Multilevel governance.Manchester:European Policy and Research Unit, Manchester University;2002.

For peer review only - http://bmjopen.bmj.com/site/about/guidelines.xhtml

60. Franklin P, Greer LS & Falkenbach M.Policy coherence as a driver of health equity.Denmark: WHO Regional office for Europe;2019.
61. Bacchi. C. Problematization in health policy:Questioning how “problems” are constituted in policies.SAGE Open.2016;1-16.
62. Thaler HR & Sunstein RC.Nudge Improving decisions about health, wealth & Happiness.Yale University Press:New Haven& London;2008.
63. Giusti D, Criel B & Bethune DX.Viewpoint:public versus private health care debate beyond the slogans. Health Policy Plan.1997;12(3):193-98.
64. Hannes K. Chapter 4: Critical appraisal of qualitative research. In: Noyes J, Booth A, Hannes K, Harris J, Lewin S, Lockwood C(editors), Supplementary guidance fr inclusion of qualitative research in Cochare systematic reviews of interventions.Version 1(updated August 2011). Cochare Colloboration Qualitataive Methods Group; 2011.
65. Ciliska D, Miccouci S, Dobbins M & Thoas HP.Effective Public Health Practice Project. Quality assessment tool for quantitative studies. Hamilton, on: Effective Public Health Practice Project;1998. Available at: https://merst.ca/ephpp/. Acessed Feb 03 2020.

Figure legends

Figure 1:The three main and the two cross cutting categories of patient rights.

Source:Townend et al, 2016.

Figure 2:The initial conceptual framework on the implementation of patient rights policies at health facility level from a multilevel governance perspective

Figure 3:Article selection and analysis algorithm

Supplementary files

Supplementary file 1

For peer review only

For peer review only - http://bmjopen.bmj.com/site/about/guidelines.xhtml

Search template for the selected databases , grey literature, citation and reference tracking

Supplementary file 2

Quality appraisal criteria for qualitative studies

Suppplementary file 3

Quality appraisal criteria for quantitative studies

Supplementary file 4

Data extraction sheet

Authors contribution

MP and SVB led the conceptualization and the design of the protocol. MP drafted the manuscript. All authors (MP,SVB,BC,NE,AK,PBN and PNS) provided inputs , have read and approved this manuscript.

Funding statement

This work is supported by the PhD fellowship offered to Meena Putturaj by the Institute of Tropical Medicine, Antwerp, Directorate of Development Cooperation, Belgium.The time and the contributions of PNS for this project is supported by the Wellcome Trust/DBT India Alliance Intermediate Clinical and Public Health Research Fellowship awarded to him [IA/CPHI/16/1/502648].

Competing interests

The authors declare that they have no competing interests. The funders do not have any role in the design and the outcomes of this research project.

For peer review only - http://bmjopen.bmj.com/site/about/guidelines.xhtml

For peer review only

Basic individual rights	Social rights	Consumer-based rights
• Informed consent<br>• Privacy – confidentiality<br>• Access to medical record<br><br>•***Information*** about one's health and treatment options<br><br>•***Procedural*** (complain, redress, participation)	•Access to health care<br>•Equal treatment<br><br>•***Information*** on rights and entitlements (including the basket of care)<br><br>•***Procedural*** (complain, redress, participation	•Safe and timely treatment •Choice - Second opinion<br><br>•***Information*** on providers •***Procedural*** (complain, redress, participation)

Fig 1:The three main and the two cross cutting categories of patients rights'

Source: Townend et al, 2016

peer review only

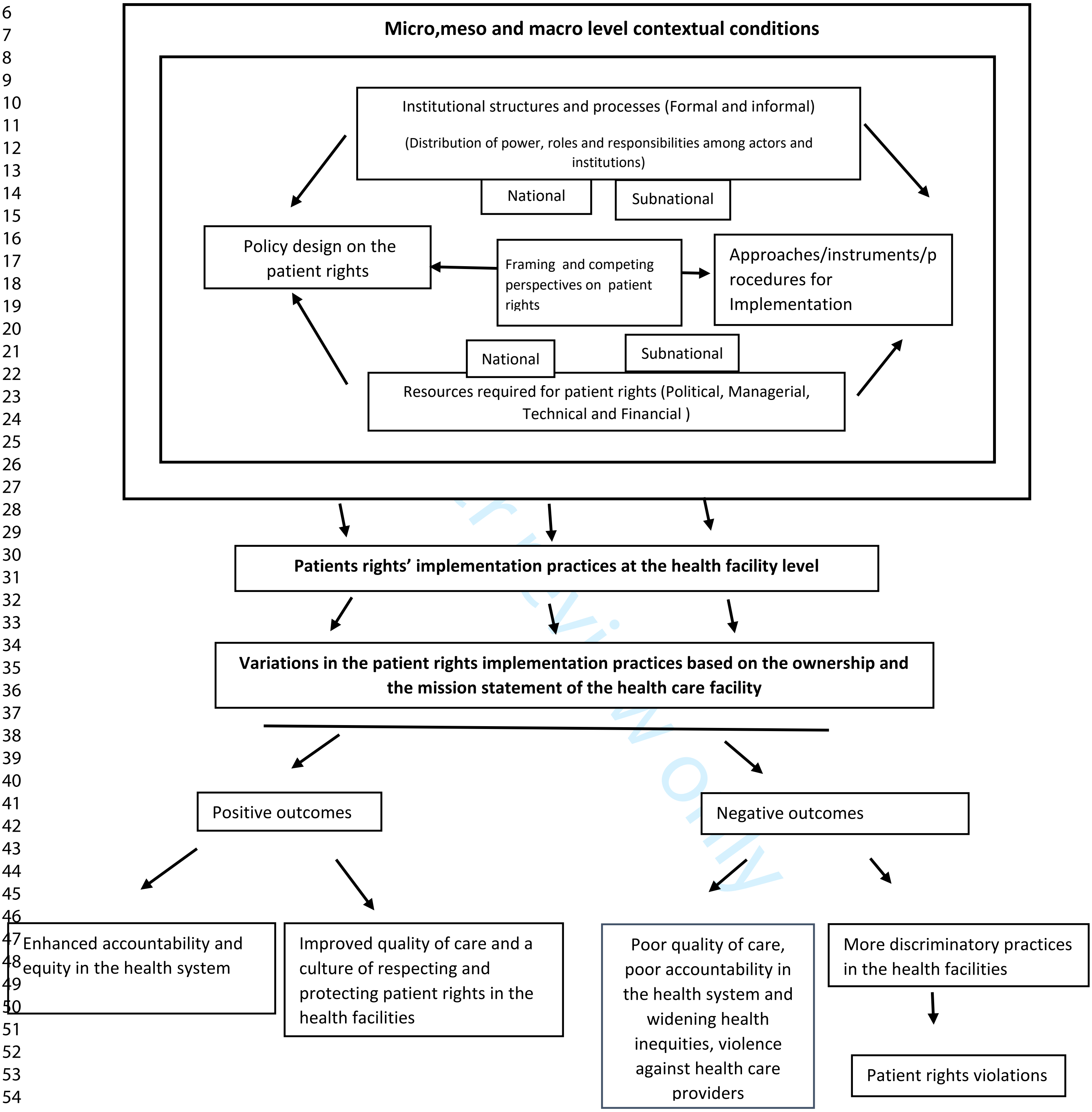

Fig 2:The initial conceptual framework on the implementation of patients right's policies at the health facility level from a multi-level governance perspective

For peer review only - http://bmjopen.bmj.com/site/about/guidelines.xhtml

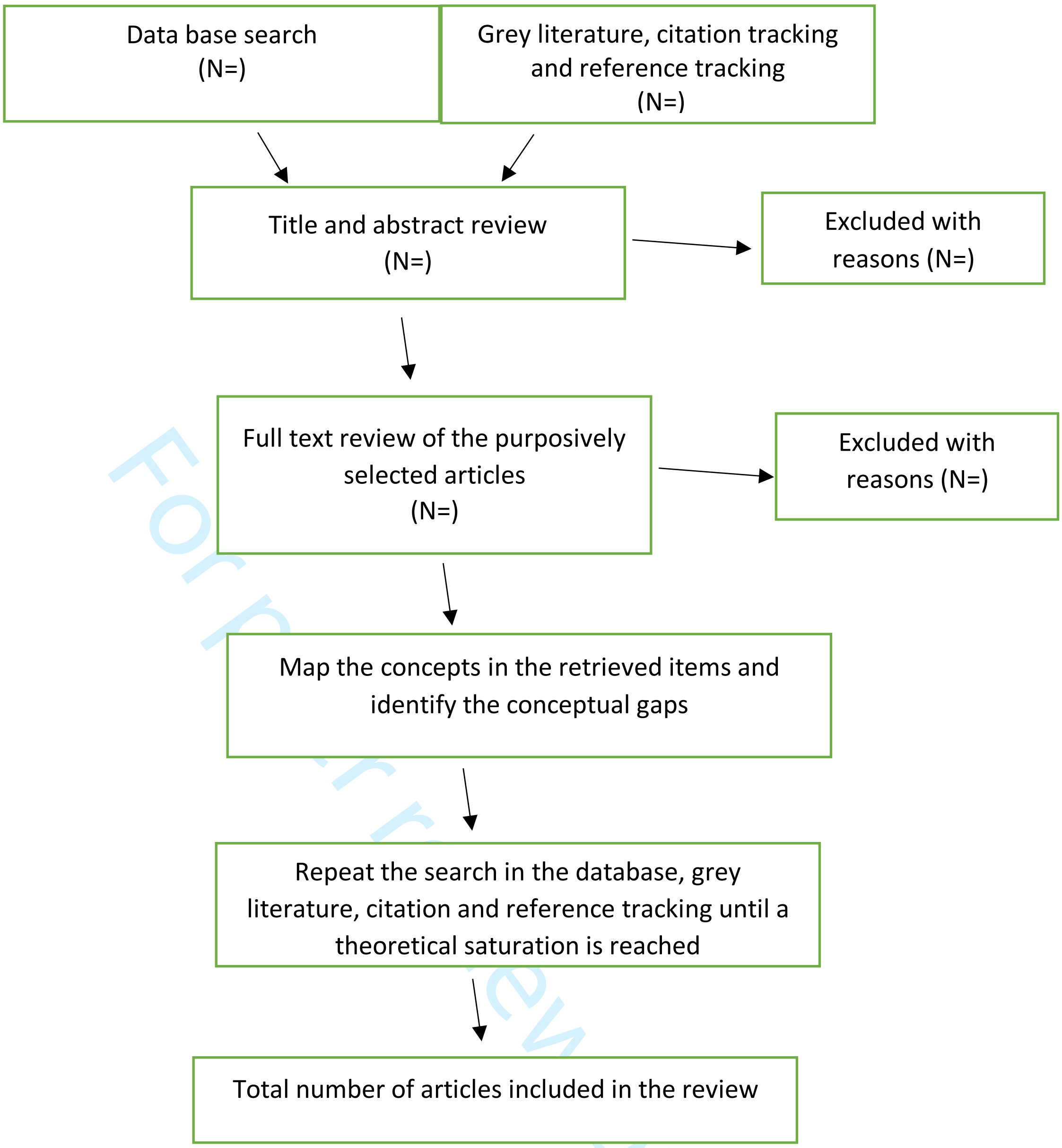

Fig3 : Article selection and analysis algorithm

For peer review only

Search template for the databases and grey literature

1. PubMed

Search terms	No of records yielded

2. Lexis Nexis

Search terms	No of records yielded

3. Web of Sciences

Search terms	No of records yielded

4. Grey literature, citation and reference tracking

	No of records yielded
**Websites**	
**Citation tracking**	
**Reference tracking**	

For peer review only

Quality appraisal criteria for the qualitative studies

Criteria	Study 1	Study 2	Study 3	Study …
Credibility				
Transferability				
Dependability				
Confirmability				
Overall quality	H/L/Judge			
Comments				

Note:

- To give more weight to studies that score high on quality
- Sensitivity analyses could be done to strike a balance between the methodological rigor and conceptual breadth and depth

For peer review only

QUALITY ASSESSMENT TOOL FOR QUANTITATIVE STUDIES

EPHPP
Effective Public Health Practice Project

COMPONENT RATINGS

A) SELECTION BIAS

(Q1) Are the individuals selected to participate in the study likely to be representative of the target population?

1 Very likely
2 Somewhat likely
3 Not likely
4 Can't tell

(Q2) What percentage of selected individuals agreed to participate?

1 80 - 100% agreement
2 60 – 79% agreement
3 less than 60% agreement
4 Not applicable
5 Can't tell

RATE THIS SECTION	STRONG	MODERATE	WEAK
See dictionary	1	2	3

B) STUDY DESIGN

Indicate the study design

1 Randomized controlled trial
2 Controlled clinical trial
3 Cohort analytic (two group pre + post)
4 Case-control
5 Cohort (one group pre + post (before and after))
6 Interrupted time series
7 Other specify ______________________
8 Can't tell

Was the study described as randomized? If NO, go to Component C.

No Yes

If Yes, was the method of randomization described? (See dictionary)

No Yes

If Yes, was the method appropriate? (See dictionary)

No Yes

RATE THIS SECTION	STRONG	MODERATE	WEAK
**See dictionary**	1	2	3

For peer review only

For peer review only - http://bmjopen.bmj.com/site/about/guidelines.xhtml

C) CONFOUNDERS

(Q1) Were there important differences between groups prior to the intervention?

1 Yes
2 No
3 Can't tell

The following are examples of confounders:

1 Race
2 Sex
3 Marital status/family
4 Age
5 SES (income or class)
6 Education
7 Health status
8 Pre-intervention score on outcome measure

(Q2) If yes, indicate the percentage of relevant confounders that were controlled (either in the design (e.g. stratification, matching) or analysis)?

1 80 – 100% (most)
2 60 – 79% (some)
3 Less than 60% (few or none)
4 Can't Tell

RATE THIS SECTION	STRONG	MODERATE	WEAK
See dictionary	1	2	3

D) BLINDING

(Q1) Was (were) the outcome assessor(s) aware of the intervention or exposure status of participants?

1 Yes
2 No
3 Can't tell

(Q2) Were the study participants aware of the research question?

1 Yes
2 No
3 Can't tell

RATE THIS SECTION	STRONG	MODERATE	WEAK
See dictionary	1	2	3

E) DATA COLLECTION METHODS

(Q1) Were data collection tools shown to be valid?

1 Yes
2 No
3 Can't tell

(Q2) Were data collection tools shown to be reliable?

1 Yes
2 No
3 Can't tell

RATE THIS SECTION	STRONG	MODERATE	WEAK
See dictionary	1	2	3

For peer review only - http://bmjopen.bmj.com/site/about/guidelines.xhtml

F) WITHDRAWALS AND DROP-OUTS

(Q1) Were withdrawals and drop-outs reported in terms of numbers and/or reasons per group?

1 Yes
2 No
3 Can't tell
4 Not Applicable (i.e. one time surveys or interviews)

(Q2) Indicate the percentage of participants completing the study. (If the percentage differs by groups, record the lowest).

1 80 -100%
2 60 - 79%
3 less than 60%
4 Can't tell
5 Not Applicable (i.e. Retrospective case-control)

RATE THIS SECTION	STRONG	MODERATE	WEAK	
See dictionary	1	2	3	Not Applicable

G) INTERVENTION INTEGRITY

(Q1) What percentage of participants received the allocated intervention or exposure of interest?

1 80 -100%
2 60 - 79%
3 less than 60%
4 Can't tell

(Q2) Was the consistency of the intervention measured?

1 Yes
2 No
3 Can't tell

(Q3) Is it likely that subjects received an unintended intervention (contamination or co-intervention) that may influence the results?

4 Yes
5 No
6 Can't tell

H) ANALYSES

(Q1) Indicate the unit of allocation (circle one)

community organization/institution practice/office individual

(Q2) Indicate the unit of analysis (circle one)

community organization/institution practice/office individual

(Q3) Are the statistical methods appropriate for the study design?

1 Yes
2 No
3 Can't tell

(Q4) Is the analysis performed by intervention allocation status (i.e. intention to treat) rather than the actual intervention received?

1 Yes
2 No
3 Can't tell

For peer review only - http://bmjopen.bmj.com/site/about/guidelines.xhtml

GLOBAL RATING

COMPONENT RATINGS
Please transcribe the information from the gray boxes on pages 1-4 onto this page. See dictionary on how to rate this section.

**A**	**SELECTION BIAS**	**STRONG**	**MODERATE**	**WEAK**	
		1	2	3	
**B**	**STUDY DESIGN**	**STRONG**	**MODERATE**	**WEAK**	
		1	2	3	
**C**	**CONFOUNDERS**	**STRONG**	**MODERATE**	**WEAK**	
		1	2	3	
**D**	**BLINDING**	**STRONG**	**MODERATE**	**WEAK**	
		1	2	3	
**E**	**DATA COLLECTION METHOD**	**STRONG**	**MODERATE**	**WEAK**	
		1	2	3	
**F**	**WITHDRAWALS AND DROPOUTS**	**STRONG**	**MODERATE**	**WEAK**	
		1	2	3	Not Applicable

GLOBAL RATING FOR THIS PAPER (circle one):

1 STRONG (no WEAK ratings)
2 MODERATE (one WEAK rating)
3 WEAK (two or more WEAK ratings)

With both reviewers discussing the ratings:

Is there a discrepancy between the two reviewers with respect to the component (A-F) ratings?

No Yes

If yes, indicate the reason for the discrepancy

1 Oversight
2 Differences in interpretation of criteria
3 Differences in interpretation of study

Final decision of both reviewers (circle one):

1 STRONG
2 MODERATE
3 WEAK

For peer review only

Data extraction sheet

Items	Article 1	Article 2	Article 3	Article n
Author				
Year of Publication				
Study setting				
Type of research design				
Type of patient rights enforcement strategy				
Type of patient rights enforcement tool/instrument				
Description of the tool				
How the tool is expected to work (the mechanism)				
Purpose (the why of the tool)				
Level at which the patient rights instrument is operationalized				
Context (macro, meso and micro) in which the tool is operationalized				
Actors involved – their competencies				
Institutions involved- their competencies				
resources required to implement the strategy/ tool				
reported outcomes				
Equity effects				

For peer review only

Preferred Reporting Items for Systematic reviews and Meta-Analyses extension for Scoping Reviews (PRISMA-ScR) Checklist

SECTION	ITEM	PRISMA-ScR CHECKLIST ITEM	REPORTED ON PAGE #
**TITLE**			
Title	1	Identify the report as a scoping review.	
**ABSTRACT**			
Structured summary	2	Provide a structured summary that includes (as applicable): background, objectives, eligibility criteria, sources of evidence, charting methods, results, and conclusions that relate to the review questions and objectives.	
**INTRODUCTION**			
Rationale	3	Describe the rationale for the review in the context of what is already known. Explain why the review questions/objectives lend themselves to a scoping review approach.	
Objectives	4	Provide an explicit statement of the questions and objectives being addressed with reference to their key elements (e.g., population or participants, concepts, and context) or other relevant key elements used to conceptualize the review questions and/or objectives.	
**METHODS**			
Protocol and registration	5	Indicate whether a review protocol exists; state if and where it can be accessed (e.g., a Web address); and if available, provide registration information, including the registration number.	
Eligibility criteria	6	Specify characteristics of the sources of evidence used as eligibility criteria (e.g., years considered, language, and publication status), and provide a rationale.	
Information sources*	7	Describe all information sources in the search (e.g., databases with dates of coverage and contact with authors to identify additional sources), as well as the date the most recent search was executed.	
Search	8	Present the full electronic search strategy for at least 1 database, including any limits used, such that it could be repeated.	
Selection of sources of evidence†	9	State the process for selecting sources of evidence (i.e., screening and eligibility) included in the scoping review.	
Data charting process‡	10	Describe the methods of charting data from the included sources of evidence (e.g., calibrated forms or forms that have been tested by the team before their use, and whether data charting was done independently or in duplicate) and any processes for obtaining and confirming data from investigators.	
Data items	11	List and define all variables for which data were sought and any assumptions and simplifications made.	
Critical appraisal of individual sources of evidence§	12	If done, provide a rationale for conducting a critical appraisal of included sources of evidence; describe the methods used and how this information was used in any data synthesis (if appropriate).	
Synthesis of results	13	Describe the methods of handling and summarizing the data that were charted.	

For peer review only

SECTION	ITEM	PRISMA-ScR CHECKLIST ITEM	REPORTED ON PAGE #
**RESULTS**			
Selection of sources of evidence	14	Give numbers of sources of evidence screened, assessed for eligibility, and included in the review, with reasons for exclusions at each stage, ideally using a flow diagram.	
Characteristics of sources of evidence	15	For each source of evidence, present characteristics for which data were charted and provide the citations.	
Critical appraisal within sources of evidence	16	If done, present data on critical appraisal of included sources of evidence (see item 12).	
Results of individual sources of evidence	17	For each included source of evidence, present the relevant data that were charted that relate to the review questions and objectives.	
Synthesis of results	18	Summarize and/or present the charting results as they relate to the review questions and objectives.	
**DISCUSSION**			
Summary of evidence	19	Summarize the main results (including an overview of concepts, themes, and types of evidence available), link to the review questions and objectives, and consider the relevance to key groups.	
Limitations	20	Discuss the limitations of the scoping review process.	
Conclusions	21	Provide a general interpretation of the results with respect to the review questions and objectives, as well as potential implications and/or next steps.	
**FUNDING**			
Funding	22	Describe sources of funding for the included sources of evidence, as well as sources of funding for the scoping review. Describe the role of the funders of the scoping review.	

JBI = Joanna Briggs Institute; PRISMA-ScR = Preferred Reporting Items for Systematic reviews and Meta-Analyses extension for Scoping Reviews.
* Where *sources of evidence* (see second footnote) are compiled from, such as bibliographic databases, social media platforms, and Web sites.
† A more inclusive/heterogeneous term used to account for the different types of evidence or data sources (e.g., quantitative and/or qualitative research, expert opinion, and policy documents) that may be eligible in a scoping review as opposed to only studies. This is not to be confused with *information sources* (see first footnote).
‡ The frameworks by Arksey and O'Malley (6) and Levac and colleagues (7) and the JBI guidance (4, 5) refer to the process of data extraction in a scoping review as data charting.
§ The process of systematically examining research evidence to assess its validity, results, and relevance before using it to inform a decision. This term is used for items 12 and 19 instead of "risk of bias" (which is more applicable to systematic reviews of interventions) to include and acknowledge the various sources of evidence that may be used in a scoping review (e.g., quantitative and/or qualitative research, expert opinion, and policy document).

From: Tricco AC, Lillie E, Zarin W, O'Brien KK, Colquhoun H, Levac D, et al. PRISMA Extension for Scoping Reviews (PRISMA-ScR): Checklist and Explanation. Ann Intern Med. ;169:467–473. doi: 10.7326/M18-0850

For peer review only

BMJ Open

Towards a multi-level governance framework on the implementation of patient rights in health facilities:a protocol for a systematic scoping review

Journal:	*BMJ Open*
Manuscript ID	bmjopen-2020-038927.R1
Article Type:	Protocol
Date Submitted by the Author:	03-Aug-2020
Complete List of Authors:	PUTTURAJ, MEENA; The University of Trans-disciplinary Health Sciences and Technology, Centre for local health traditions and policy<br>Van Belle, Sara; Department of Public Health., Institute of Tropical Medicine<br>Criel, Bart; Institute of Tropical Medicine, Department of Public Health<br>Engel, Nora; Maastricht University,<br>Krumeich, Anja; Maastricht University, Department of Health, Ethics and Society<br>B Nagendrappa, Prakash; The University of Trans-disciplinary Health Sciences and Technology, Centre for local health traditions and policy<br>Prashanth, NS; Institute of Public Health,
**Primary Subject Heading**:	Health policy
Secondary Subject Heading:	Health services research, Patient-centred medicine, Public health, Qualitative research
Keywords:	Health policy < HEALTH SERVICES ADMINISTRATION & MANAGEMENT, PUBLIC HEALTH, MEDICAL ETHICS, QUALITATIVE RESEARCH

SCHOLARONE™
Manuscripts

I, the Submitting Author has the right to grant and does grant on behalf of all authors of the Work (as defined in the below author licence), an exclusive licence and/or a non-exclusive licence for contributions from authors who are: i) UK Crown employees; ii) where BMJ has agreed a CC-BY licence shall apply, and/or iii) in accordance with the terms applicable for US Federal Government officers or employees acting as part of their official duties; on a worldwide, perpetual, irrevocable, royalty-free basis to BMJ Publishing Group Ltd ("BMJ") its licensees and where the relevant Journal is co-owned by BMJ to the co-owners of the Journal, to publish the Work in this journal and any other BMJ products and to exploit all rights, as set out in our licence.

The Submitting Author accepts and understands that any supply made under these terms is made by BMJ to the Submitting Author unless you are acting as an employee on behalf of your employer or a postgraduate student of an affiliated institution which is paying any applicable article publishing charge ("APC") for Open Access articles. Where the Submitting Author wishes to make the Work available on an Open Access basis (and intends to pay the relevant APC), the terms of reuse of such Open Access shall be governed by a Creative Commons licence – details of these licences and which Creative Commons licence will apply to this Work are set out in our licence referred to above.

Other than as permitted in any relevant BMJ Author's Self Archiving Policies, I confirm this Work has not been accepted for publication elsewhere, is not being considered for publication elsewhere and does not duplicate material already published. I confirm all authors consent to publication of this Work and authorise the granting of this licence.

For peer review only

Towards a multi-level governance framework on the implementation of patient rights policies in health facilities:a protocol for a systematic scoping review

Meena Putturaj[1,2,3,4], Sara Van Belle [1], Bart Criel[1], Nora Engel[2], Anja Krumeich[2], Prakash BN[3] & Prashanth NS [4]

Institutions

1.Institute of Tropical Medicine, Antwerp, Belgium

2.Maastricht University, Netherlands

3.The University of Transdiscplinary Health Sciences and Technology, Bengaluru, India

4.Institute of Public Health, Bengaluru,India

Authors and Author's affiliations

1. Meena Putturaj *(Corresponding author)*[1,2,3,4]
Centre for Local Health Traditions and Policy
The University of Trans -Disciplinary Health Sciences and Technology
74/2, Post Attur via Yelahanka, Jarakabande Kaval, Bengaluru, Karnataka 560064, India
meenaputturaj@gmail.com

2.Dr.Sara Van Belle[1]
svanbelle@itg.be

3.Dr.Bart Criel[1]
Bcriel@itg.be

4.Dr. Nora Engel[2]
n.engel@maastrichtuniversity.nl

5. Dr. Anja Krumeich[2]
a.krumeich@maastrichtuniversity.nl

6.Dr. Prakash BN[3]
bn.prakash@tdu.edu.in

7.Dr. Prashanth Nuggehalli Srinivas[4].
prashanthns@iphindia.org

Word count: 5132 excluding the title page, abstract, references, figures and tables

For peer review only - http://bmjopen.bmj.com/site/about/guidelines.xhtml

Towards a multi-level governance framework on the implementation of patient rights in health facilities:a protocol for a systematic scoping review

Abstract

Introduction

Patient rights are "those rights that are attributed to a person seeking health care". Patient rights have implications for quality of health care and acts as a key accountability tool. It can galvanize structural improvements in the health system and reinforces ethical health care. States are duty bound to respect, protect and promote patient rights. The rhetoric on patient rights is burgeoning across the globe. With changing modes of governance arrangements, a number of state and non-state actors and institutions at various levels play a role in the design and implementation of (patient rights)policies. However, there is limited understanding on the multilevel institutional mechanisms for patient rights implementation in health facilities. We attempt to fill this gap by critically analyzing the available scholarship on patient rights through a critical interpretive synthesis approach in a systematic scoping review.

Methods

The review question is "how does the multilevel actors, institutional structures, processes interact and influence the patient rights implementation in health care facilities? How do they work at what level and in which contexts?"Three databases PubMed, Lexis Nexis and Web of Science will be systematically searched for empirical and non- empirical literature in English from both LMIC and HIC. Targeted search will be performed in grey literature and through citation and reference tracking of key records. Using the critical interpretive

For peer review only - http://bmjopen.bmj.com/site/about/guidelines.xhtml

synthesis approach , a multilevel governance framework on the implementation of patient rights in health facilities which is grounded in the data will be developed. The review will be conducted between 15/04/2020 and 15/08/2020.

Ethics & Dissemination

The review uses published literature hence ethics approval is not required. The findings of the review will be published in a peer reviewed journal.

Registration

This review is registered with PROSPERO and the registration ID is CRD2020176939.

Key words

Patient rights implementation, critical interpretive synthesis, multi-level governance

Strengths and limitations of this study

- The proposed review will deliver an explanatory framework on the patient rights implementation in health facilities from a multilevel governance perspective
- The use of literature from multiple disciplines provide a nuanced understanding of the contested concepts such as the patient rights that are complex involving many stakeholders and tradeoffs
- The Critical Interpretive Synthesis (CIS) approach to synthesize the evidence is a perfect fit to understand the nature of complex fuzzy concepts such as a patient rights implementation as it enables the use of data emerging from heterogenic disciplines and methodologies

For peer review only - http://bmjopen.bmj.com/site/about/guidelines.xhtml

- The application of multilevel governance as the analitycal lens is useful to study what vertical and horizontal levels and in what roles various state, market, and civil society actors and institutions interact for the implementation of patient rights.
- Considering the time frame available for the review, the following are not within the scope of this review 1.Patient rights implementation by the informal health care providers 2. Patient rights concerning mental health disorders, disabilities, genetic screening, euthanasia, advance medical directives and health research as they merit specific considerations 3. Patient responsibilities/obligations 4. International mechanisms for patient rights protection(e.g. United Nations Human Rights Council).

Introduction

Human rights are believed to be the rights and freedoms that are inherent to every person in this world without any bias. Formally in 1948, in the aftermath of the World War II, the "UN Declaration of Human Rights" laid the foundation for defining fundamental rights shared by every human being (e.g. right to life, liberty and security, right to equality, freedom from torture etc.). These rights proclaimed in the Declaration were adopted by the UN General Assembly and ever since considered as the norm(s) by all nations for all people [1]. However, cultural relativists and post-colonial critics argue that the universal human rights doctrine in a multicultural society is an imposition of Western philosophy, values and norms on non-Western cultures[2]. Raimon Panikkar, an Indian philosopher, argued that despite human rights being a Western notion, it can still act as an instrument for social justice in non-Western settings e.g. India. However, a cross-cultural philosophical approach to human rights is vital [3].

For peer review only - http://bmjopen.bmj.com/site/about/guidelines.xhtml

For peer review only

Embedded within the broader perspective of human rights is the notion of “patients rights”[4-6]. Patient rights refer to “those rights attributed to a person seeking health care”[7]. It is the application of human rights principles in a health care setting - the location where health care providers offer services to the people.

Townend and his colleagues [8] **(see Figure 1)** identified five main categories of patient rights 1) *individual rights* (certain liberties guaranteed for an individual person) 2) *consumer* (one who purchases the economic services and commodities) *based rights* 3) *social rights* (emerging from social contract e.g. between state and the citizens) 4*) procedural patient rights* 5) *informational patient rights.* The last two categories (4 &5) are cross-cutting and help to enforce the first three categories of patient rights.

However, the categories mentioned above are not mutually exclusive. For example, the right to informed consent (individual right) is connected to right to information (procedural right). Likewise, the right to complain depends on the right to access medical files in order to prove that the right to informed consent was violated [8].

Why focus on patient rights’?

Because of structural inequalities such as power imbalances between the care seeking individuals and providers and other social inequities related to income gap, gender inequality, social class and stigma associated with health conditions e.g. HIV, tuberculosis, mental disorders etc, health care settings are specifically vulnerable to patient rights violations[9]. Most often, the vulnerability to patient rights violations emerges from a complex mixture of organizational and systemic factors (e.g. lack of human resources,for providing respectful care) shaping the health care provider-person encounter[10]. There is considerable evidence regarding the prevalence of paternalism in the medical

For peer review only - http://bmjopen.bmj.com/site/about/guidelines.xhtml

profession[11-15]. This has given rise to the scholarly work on people-centered care models emphasizing patient autonomy and joint decision-making in the health care processes [16].

The importance of patient rights could be gleaned from multiple perspectives:

i) From the perspective of patient safety

In a traditional hierarchical relationship, patients might refrain from having an open communication with health care providers or the health care providers may not provide in-depth information to people seeking care . This lack of open communication might deter patients from participating in the discussions related to the prevention of health care related errors and thus have implications for patient safety. Patient rights provide a language to empower and engage the care seeking individuals in the processes of delivering health care[17].

ii) From the perspective of quality of health care

The six dimensions of quality health care are safety, timeliness/accessibility, patient centeredness/acceptability, equity, efficiency and effectiveness [18,19]. Most of the components of patient rights align with the key dimensions of quality health care. For instance, scholars have reiterated the need for respecting the patient rights as a tangible entry point for achieving people- centered care[20,21].Thus, the notion of patient rights is in consonance with the concepts of quality of health care and people-centered care.

Iii) From the health system perspective

Furthermore, patient rights might galvanize improvements at health system level by infusing accountability through oversight mechanisms [22]. Efficient and functional patient complaints and grievance redressal systems offer critical lessons for the structural

For peer review only

improvement of the health system[23].Evidence from UK, New-Zealand and Canada show that the recommendations of patient rights and safety ombudsmen have the potential to influence policy makers at the highest level [24-26].

iv) From an ethical perspective

Patient rights are deeply interwoven with the four core principles of medical/ health care ethics i.e. beneficence (do good), non - maleficence (do no harm), justice and autonomy [4]. Patient rights and the principles of medical ethics complement each other. Any deviation of the health care professional's practice from medical ethics is often equated with the violation of patient rights. See for instance, the Mid-Staffordshire National Health System (NHS) scandal in UK which revealed the preventable deaths of patients because of medical negligence by the health care staff[27], evoked concerns on the breach of patient's dignity and their right to quality care in the NHS[9].

v) From the health equity perspective

Research studies from both high-income contexts (e.g. USA, Europe)[28,29] and LMICs (e.g. India, Kyrgyzstan) [30-32] reveal the existence of discriminatory practices towards people receiving care. The discrimination could be based on single grounds (e.g. HIV status of a person, a migrant in Europe, sex worker etc.) or on the complex intersections of multiple grounds (e.g. age, gender, ethnicity, socio-economic status, disability etc.). The growing global consensus for respectful maternity care is a case in point that illustrates the magnitude of the patient rights violations experienced by the vulnerable populations (childbearing women) at all levels of health care[33]. It is acknowledged that strengthening accountability in the health system will help tackle health inequities[34]. Patient rights could serve as a health system accountability tool [22,28].

For peer review only - http://bmjopen.bmj.com/site/about/guidelines.xhtml

Rationale

The social, cultural and the policy context shape the design and the implementation of patient rights resulting in variations across the settings [6]. For example, on comparing the content of the patient rights charter agreed by the European Union[35] with that of the Indian[36] and the Ugandan patient bill of rights[37], the European Union patient rights charter had included “right to innovation” “right to avoid unnecessary suffering and pain” “right to personalized treatment” “right to respect patients time”. These rights were not mentioned in the Indian and the Ugandan bills of patient rights. The levers to enforce the patient rights also vary between countries. In countries like UK[38], Netherlands[8], Sweden[8], Canada[39] patient rights are enshrined in law and there are separate statutory bodies. For example, health ombudsmen (i.e. an independent public authority who deals with citizens’ complaints against the public/private institutions) are available to people who wish to complain when they feel that their rights are not respected. The National Health System of UK also provides assistance to the care seeking individuals to navigate the grievance redressal system[38]. Inadequate institutional mechanisms to respect, protect and fulfill human (patients) rights in certain Lower middle -income countries (LMIC) (e.g. India, South Africa, Brazil) are increasingly compensated by citizen action and strategic litigation steered by civil society organizations[22,40,41].

Patient rights movements and civil society organizations arguing for improved accountability in health care are gathering momentum across the globe and more so in LMICs [40]. Although the rhetoric on patient rights is burgeoning, empirical research in this area is nascent and most of the existing research studies [42-45] limit themselves to studying awareness of patient rights. Also, the available frameworks in the literature identify the

For peer review only - http://bmjopen.bmj.com/site/about/guidelines.xhtml

For peer review only

typology of patients rights [8] and a few other attempt to situate the patients rights' within the larger human rights framework [4]. According to the human rights law, states are duty-bound to protect human rights vis-a-vis patient rights[1]. Often in LMICs, the failure of the health care related policies is attributed to ineffective and inefficient governance [46].There is limited scholarly work that shed light on the influence of the governance arrangements on patient rights implementation.

We attempt to fill this gap by critically reviewing the existing scholarship on patient rights and come up with a framework on the implementation of patient rights from a multilevel governance perspective. The concept of governance is difficult to define. Most of the definitions emphasize *power, authority and accountability* as the key aspects of governance[47]. Governance is not anymore a role played only by the governments. A broad range of actors and institutions (both state and non-state) such as the civil society, media, professional groups engage at different levels of system depending on their power and interest in the policy problem [48]. The emergence of concepts such as the multi level governance, multi tiered governance, polycentric governance, multi perspectival governance , network governance indicate the diffusion of authority from the centre to more regional and local levels thus bringing governance systems close to the communities. Multi level governance also highlights the proliferation of non-state actors in the public policy processes. In simple terms, multi-level governance refers to the dispersion of governance across multiple jurisdictions and multiple actors (international, national, subnational). The idea of multilevel governance was first applied to explain the European Union politics[49]. The concept was later used to explore the governance of common pool resources e.g. land use, irrigation systems, renewable energy and community fisheries as well as in the analysis of health care system and organization of primary health care

For peer review only - http://bmjopen.bmj.com/site/about/guidelines.xhtml

services[48,50]. There are two varieties. In type I multi level governance the jurisdictions are defined clearly by the territorial boundaries and the functions of governance are confined to these territorial boundaries e.g.Court system. An alternative form is the type II multilevel governance where the jurisdictions often overlap and operate at huge territorial scales depending on the nature of the policy problem addressed. e.g. monitoring water quality of a particular river, resolve conflicts pertaining to common pool resources etc[49]. Divay and Paquin (2013) argued that multilevel governance is not merely restricted to intergovernmental relations rather it involves flexibility in role sharing between various stakeholders (both state and non state) at multiple levels in the governance processes [as cited in 51]. For our systematic scoping review we consider the conceptualization of multi level governance as the one that is characterized by "the study of the crossroads of the vertical (intergovernmental) and horizontal (state-society) dimensions"[52]. Also, policy design and implementation is a matter of continuous negotiation and cross level interactions among the diverse set of actors within the nested jurisdictions[50,53]. Power relations drive the cross level interactions in the multilevel governance systems [50]. We propose that multilevel governance as the analytical lens is useful to study what vertical and horizontal levels in the governance system and in what roles various state, market, and civil society actors interact for the implementation of patient rights.

In the field of evidence synthesis, systematic scoping reviews are considered as an appropriate approach to answer complex questions where systematic reviews are not possible, or do not produce the required (conceptually rich) information. Unlike the systematic reviews, systematic scoping reviews are applied for investigating broad questions that are interdisciplinary in nature but in a systematic manner and does not involve strict

For peer review only - http://bmjopen.bmj.com/site/about/guidelines.xhtml

exclusion criteria for the studies [54]. The proposed review uses the most recent PRISMA guidelines[55] for reporting the systematic scoping reviews**(see supplementary file 1).**

Further in this systematic scoping review, considering the limited theoretical development in the area of patient rights implementation, the critical interpretive synthesis(CIS) [56] will be used as a technique for searching and synthesizing the evidence. The CIS approach offers guidance to marshall evidence to answer complex fuzzy questions that often cut across multiple disciplines as it allows to

- use diverse body of evidence from varied forms of data sources,disciplines and methodologies
- modify the review question in response to the search results and findings of the previously retrieved items
- use literature from the adjacent fields relevant to the emerging nature of the review question
- use a pragmatic approach to identify potentially relevant and conceptually sound literature that would contribute for theory building or an explanatory framework
- critique the ways of the problematization of the phenomena under study in the literature and explore the underlying assumptions on which the solution to the social problems are constituted

For peer review only - http://bmjopen.bmj.com/site/about/guidelines.xhtml

CIS is successfully applied in the field of health systems to explore areas such as informal payments in maternal health care[57], access to health care[56], overuse of health services[58]. The concept of patient rights itself is a subject of multiple interpretations, partially owing to the confusion that exists on who is claiming these rights; is it the patient or consumer or citizen?[59].The interrelatedness of patient rights with health system accountability, quality of health care, health care ethics, and equity issues further makes it difficult to define clearly the patient rights conceptually. Further attempting to explore the " implementation" aspect of the patient rights adds another layer in the complexity owing to its overlapping with the related fields such as governance and health policy processes. Since the focus of the CIS approach is towards achieving conceptual clarity and theory development, it might help to search, retrieve

Salient features of the CIS approach [56]

- The review question serves as a compass rather than an anchor and is therefore revised and refined during the review process
- The aim is to develop synthesizing argument which could take the form of a plausible theoretical framework reflecting the network of constructs grounded in the evidence
- The inductive and iterative nature of the CIS approach demands several cycles of searching, sampling, critiquing and analyzing the evidence until a theoretical saturation is reached
- The subsequent cycles of searching, sampling, critiquing and analyzing the evidence is informed by the emerging theoretical framework
- The priority for selecting the evidence is "signal"(i.e.likely relevance to answer the review question) over "noise"(the inverse of methodological quality). However, formal quality appraisal techniques could be applied for individual papers. The aim is to purposively select papers that are most relevant and conceptually rich
- Data extraction charts initially may help in the review but not an essential feature of the CIS approach
- The review may not not be strictly reproducible, auditable. It can be argued that even with primary data in a qualitative study or other evidence synthesizing techniques it is most likely that a different set of researchers may interpret the same data/ set of evidence and come up with the different theoretical model.
- The synthesizing argument "theoretical framework" is a product of interpretive process and" authorial voice" .Therefore there is a need for constant reflexivity throughout the review process on the part of the authors.

For peer review only - http://bmjopen.bmj.com/site/about/guidelines.xhtml

and synthesize from the literature on patient rights implementation that is diverse and heterogenic in terms of methods and disciplines.

The initial review question

How does the multilevel actors, institutional structures, processes interact and influence the patient rights implementation in health care facilities? How do they work at what level and in which contexts?

Objectives

The objectives of this review are two fold

1)To describe the institutional arrangements, strategies, approaches and instruments at various levels for patient rights implementation in health facilities 2)To propose a theoretical framework on the implementation of patient rights in health facilities from a multilevel governance perspective

Methods

Operational definitions of the key terminologies used in the review

Patient rights- In this review the scope of patient rights covers the patient rights applicable to facility-based preventive, promotive , curative and rehabilitative health care services offered in the primary, secondary and tertiary level health care settings

Patient rights implementation approach refers to the overall strategy and the plan of action taken by the state and/or the various non-state actors such as communities, non-governmental organizations, civil society organizations and professional bodies to respect, protect and fulfill the patient rights.

For peer review only - http://bmjopen.bmj.com/site/about/guidelines.xhtml

Patient rights implementation instruments refers to the set of procedures, methods and tools available to implement patient rights. Examples include patient grievance redressal committees, patient rights charter, ombudsman, public hearing, parliamentary hearing, strategic litigation by NGO(s), public protests & patient directly filing a petition in the court system.

Patient rights implementation mechanisms refers to the means and the ways through which a particular patient rights enforcement strategy/ instrument is expected to work in a given context

Multilevel governance is about the interaction of the vertical (intergovernmental) and the horizontal (state society dimension) institutions/actors at various levels for the implementation of patient rights in health facilities.

The initial conceptual framework to guide the evidence synthesis

Since the focus of this review is the instutional mechanisms for patient rights implementation,the initial conceptual framework **(see Figure 2)** is developed from the concepts derived from the literature on patients rights [4,18,21-23,28-33] and public policy implementation [60-62]. To explore the institutional dimension of patient rights we embedded the fit, interplay and scale framework on multi level governance in our conceptual scheme[63]. The fit, interplay and the scale themes are in alignment with the broader definition of multilevel governance adopted for this review. The role of language and discourse is critical in the study of multilevel governance[63]. Therefore, we adopted the idea of discourse analysis[64] on patient rights in this initial framework. This starter framework will be further refined using the CIS technique in this systematic scoping review.

For peer review only - http://bmjopen.bmj.com/site/about/guidelines.xhtml

The framework could be interpreted as a linear one which may not be the case. The elements of the framework might be inter related in a number of ways.

Patient rights implementation approaches

The implementation approaches to respect,protect and promote patient rights in health facilities could vary across the settings. A number of factors might determine the implementation approaches. To name a few, the political ideology of the state, political framing of the (patient) rights issues, type of health systems and availability of resources for delivering quality health care. The failure of the state's institutional mechanisms to adequately protect the patient rights may result in alternative institutional arrangements steered by the non-state actors such as the professional associations, state independent health care quality accrediting bodies, civil society, media etc.

Fit, interplay and the scale of institutions

The key element of the framework is the institutional dimension of patient rights implementation. By institutions we refer to the thick and the thin definitions of institutions. In the thin sense , institutions refer to the formal explicit set of rules and decision making units i.e rules in paper. On the contrary the thick sense of institutions encapsulate the "social practices" that emerge out of informal understandings between the actors i.e. rules in use. There are three lines of inquiry in studying the institutions 1) fit – the degree to which the spatial, functional and temporal institutional arrangements fit the context. Simply put, it is to see whether the institutional arrangements fit the dynamics of policy problem addressed (in this case- patient rights implementation) 2) interplay- concerns the horizontal interaction between the same level and the vertical interaction across the different levels of the institutions and actors. There could be political or functional interdependencies across

For peer review only - http://bmjopen.bmj.com/site/about/guidelines.xhtml

the institutions. 3) scale is about the density and the degree of diversity of institutions and actors from local to higher levels. The institutions can range from homogenous to heterogenous , few to many and individual actor influence to policy communities. In general the scale could be visualized as the shift from simple to complex systems as we move higher up in the levels[63].

Resources requirements

Political, financial, managerial and technical competencies of the actors/ institutions at the national and the subnational levels define the effectiveness of policy instruments used to enforce the patient rights in health facilities*. Human resources* entail the availability of adequate and competant actors for patient rights implementation. Public engagement in the policy processes concerning patient rights is key. *Financial resources* refer to the funds that are allocated for facilitating patient rights, policies implementation activities (e.g. recruiting manpower, logistics, infrastructure, equipment, supplies, training, etc.). *Managerial resources* indicate the availability of tools and monitoring mechanisms within the patient rights' enforcement system (e.g. reporting, recording, supervision of the enforcing officials). *Technical resources* denote the existence of technical capacity and assistance for policy implementation e.g. use of information and communication technology. All of these resources will influence the accessibility and availability of the patient rights' policies in the manner comprehensible to the stakeholders, including citizens (and the most vulnerable groups) [60].

Constellation of discourses on patient rights and other contextual factors

Patient rights are within a contentious policy area. Therefore implementation of patient rights should not be seen as only a matter of clear policy design and matching resources for

For peer review only - http://bmjopen.bmj.com/site/about/guidelines.xhtml

implementation. The policy documents related to patient rights (e.g. Patient rights charter) , implementation strategies and instruments are a product of a certain context and time and hence have been framed in certain discourses. Understanding how the issues on patient rights are constituted, represented, given shape and meaning within the policies is cruial as it determines the way the implementation is finally panned out within the multiple tiers of the polity[64].

Strategies and the policies designed to respect, protect and fulfill the patient rights are embedded within a specific social,political,cultural,economic, legal and organizational context. The contextual conditions at the i) micro (facility and the individual patient provider) level e.g. nature of the patient provider relationship, meso (district and subdistrict) level e.g. organizational culture of the implementation bodies and macro level (national and subnational) e.g. economic policies, reforms in health professional education and broader societal and the cultural issues (e.g. social exclusion and discrimination based on gender, class etc will influence the implementation processes. Further, it will be an easier task to implement patient rights, when the players supposed to practice them have somehow 'internalized' the notion and the importance of patients rights'[65]. In that respect that there should be room in other policies that have implications for patient rights implementation e.g. adapting the training curricula of health care providers, health systems managers, public health professionals etc.

Multiple potential outcomes [60]

The complex interaction of the actors/institutions, the implementation approaches, instruments and the policy context at various levels and broader societal context will determine the actual implementation practices related to patient rights at the health facility

For peer review only

level. Again the patient rights implementation practices could vary based on the nature of the ownership and the mission statement of the health care institution for which it exists. For instance, there could be a private health care institution with a public goal or a public health care institution with a profit goal for sustainability reasons [66].

When the patient rights policies are implemented effectively and conducive context conditions are in place , this should result in enhanced accountability and equity in the health system [23,29,30]. Further, at the health facility level, this is expected to lead to improved quality of care[18,21,22] and to promote a culture of respecting and protecting the patient rights[4]. In case of ineffective implementation of patient rights policies/strategies, there will be more discriminatory practices in the health facilities, contributing to the denial of patient rights, widening health inequities and poor accountability in the health system[24,29-33]. Also, it is possible that in settings where there is poor response of the state to the patient rights violations, people might feel frustrated, lose trust in the health system and resort to violence against the health care providers[23].

Eligibility criteria, data sources & search strategy

There are three main data sources for this review 1) select databases 2) grey literature 3) reference tracking and citation tracking. To get a comprehensive understanding on patient rights implementation strategies and instruments from a multidisciplinary perspective, three databases PubMed (health literature), Web of Science (social sciences), Lexis Nexis (Law) will be searched in a systematic manner for all types of evidence (both empirical and non-empirical) broadly on key concepts such as patient rights, patient rights instruments and person centered care. The related terms reflecting the key concepts will be used as search terms in the databases **(see Table 1).** The search terms will be pilot tested in the

For peer review only - http://bmjopen.bmj.com/site/about/guidelines.xhtml

PubMed database. Boolean operators will be used to develop various combinations of search terms feasible for usage in the PubMed database. Followed by PubMed, a more detailed search will be undertaken in the Web of Sciences and Lexis Nexis databases. We have provided our detailed search strategy for the PubMed database in the **supplementary file 2**. Further, targeted searches will be performed in grey literature 1) Google 2) Websites of the institutions (public and private) that are relevant to the area of patient rights, social justice, human rights and accountability in health systems (e.g. Open Society Foundations, People's Health Movement , health care quality accreditation agencies, other civil society institutions(national and international), Patient rights organizations (national and international) e.g. Coalition patient rights, National Patient Advocate Organization etc. Also, the reference list of the specific papers(reference tracking) and the papers that cite the key papers (citation tracking) will be hand searched to identify any additional studies that are conceptually relevant and have the potential to contribute for the emerging framework.

Table 1:Key search domains and the related terms to be used in different permutations and combinations in the selected databases

**Key search domains**	**Related terms**
Patient rights	Patient bill of rights, patient charter, ,quality of health care, social(community) accountability in health,Patient accountability, patient safety, discrimination, health equity, equality, right to health, patient rights and ethics

For peer review only - http://bmjopen.bmj.com/site/about/guidelines.xhtml

Patient rights instruments	Patient rights legislation, patient rights charter, patient bill of rights, health ombudsman, right to information act, health councils, patient welfare committees, patient grievance redressal systems/ committees, patient complaints system, patient advocates, parliamentary hearings, public hearing, public protests, strategic litigation, consumer forums, professional associations, social audit, audit bodies, patient suggestion box, court system for patient rights, human rights commissions, health care quality councils
Person centered care	Patient centered care, people centerd care, patient autonomy, patient engagement, patient participation, patient empowerment

Note: The search terms mentioned in the table are only indicative and not exhaustive. The list will be further enriched after the initial search for the studies in the PubMed database

Period of search

The aim of the literature search is to maximise the possibility of identifying the conceptually rich articles to enable theory building hence no specific time period is stipulated for this review.

Publication type

For peer review only

For peer review only - http://bmjopen.bmj.com/site/about/guidelines.xhtml

As indicated in the state of the art literature on patient rights, it is clear that the available scholarship is mostly from the European contexts.It is expected that there will be limited empirical work on the patient rights in the LMICs. In order to increase the transferability of the theoretical framework to the LMIC settings it was decided that all types of evidence (both empirical and non-empirical) in English from both LMIC and HIC will be included in this review

Time period scheduled for the review

The review will be conducted between 15/04/2020 and 15/08/2020.

Article selection process (see Figure 3)

1. Systematic search for the articles on patient rights implementation in the selected three data bases
2. Identifying the relevant articles in grey literature and through citation and reference tracking
3. Title and abstract review of the articles to identify the relevant articles that are concordant with the initial review question
4. Creating a sampling frame of all articles
5. Purposive sampling of the articles from the sampling frame that point towards relevancy and conceptual soundness
6. Applying the quality appraisal criteria for the individual papers. Perform sensitivity analysis to strike a balance between methodological rigour and conceptual breadth and depth of the study. To give more weightage to the articles that score high on quality
7. Initial data extraction on the study characteristics

For peer review only - http://bmjopen.bmj.com/site/about/guidelines.xhtml

8. Concept mapping in the retrived literature and identification of the conceptual gaps
9. Repeating the search in the selected databases,grey literature, citation tacking and reference tracking to fill the conceptual gaps until a theoretical saturation is reached and refining the synthesizing argument i.e.“theoretical framework”on the patient rights implementation

Quality appraisal criteria

Since the review uses articles emerging from atleast three diverse disciplines (public health, social sciences and law), finding a common quality assessment framework is challenging. However, it is expected that majority of the empirical work will be of qualitative in nature. Hence, the quality of the studies will be evaluated using the Hannes criteria (2011) [67] for assessing qualitative research **(see supplementary file 3).** The main parameters for evaluation would be 1) credibility 2)transferability 3) dependability 4) confirmability. The quality of the study will be determined using a three point likert scale(strong moderate and weak). For quantitative studies, quality appraisal tool developed by Effective Public Health Practice Project[68] will be used **(see supplementary file 4).**

Data extraction

The usefulness of the formal data extraction sheet for a complex review topic that uses critical interpretive synthesis technique is questioned [56]. Due to the huge set of variables involved, a formal data extracton sheet might appear messy and practically difficult to analyse. So we devised a simple data extraction proforma which include details such as the author,author affiliations, year of publication, article type, study design, funding for the study, setting and country **(see supplementary file 5)**. The broad themes, categories and the subcategories as identified in the conceptual framework and also emerging from the data

For peer review only

would be captured and organized using the NVIVO version 12 software. The first investigator will extract the data and this will be independently checked atleast by two other senior researchers of the review team. Further the four authors MP, SVB, NE and PNS will deliberate on the data collection and the data analysis at regular intervals.

Patient and Public involvement

Patient and public involvement is not applicable in this review as the research uses already published literature.

Synthesis of results

The CIS approach used in the evidence synthesis is inductive and iterative in nature. The retrieved items/papers will be critically analysed for patterns and themes that explain the phenomena of patient rights implementation in the literature. By constant comparison of the theoretical constructs with the data in the papers, the relationships between the theoretical constructs will be identified.The conceptual gaps identified will be further filled with subsequent search and analysis of the literature.The aim is to be critical while synthesizing the evidence by questioning 1) the ways in which the patient rights has been problematized or represented to be in the literature 2) the underlying assumptions behind the policy options or the implementation strategies for patient rights.The synthesis of the evidence retrieved in this review would thus result in a unifying framework (i.e.the synthesizing argument) on the implementation of patient rights from a multilevel governance perspective that is grounded in the data.

Limitations

For peer review only

Rights and responsibilities goes together. The review focusses on the rights of the patient and we do not venture into the obligations of the patient in the health care processes. Patient rights pertaining to health research context and specific conditions such as mental health disorders, disabilities, genetic screening, euthanasia and advance medical directives merit specific consideration and hence these are out of the scope of this review. In this review we deal with the institutional arrangements for patient rights protection in the health care facilities that are formally recognized and considered legitimate by the state. We exclude the health care facilities run by the informal health care providers. The international institutional mechanisms such as the Office of the United Nations High Commissioner on Human Rights (OHCHR), the Human Rights Council etc are not touched upon in this inquiry mainly because of the time constraints.

Registration

This protocol is registered in PROSPERO (https://www.crd.york.ac.uk/prospero/display_record.php?RecordID=176939). The registration ID is CRD2020176939.

Ethics and dissemination

Ethical approval will not be required because this study will retrieve and synthesise data from already published literature.The findings of this review will be published in a peer reviewed journal.

References

For peer review only - http://bmjopen.bmj.com/site/about/guidelines.xhtml

1. United Nations. Universal Declaration of Human Rights. General Assembly resolution 217 A. Newyork: United Nations;1948. Available from: https://www.un.org/en/universal-declaration-human-rights/. Accessed 13 Jan 2020.
2. Imani O N. "Critical Impairments to Globalizing the Western Human Rights Discourse."Societies Without Borders.2008; 3 (2): 270-284.Available at:https://scholarlycommons.law.case.edu/swb/vol3/iss2/. Accessed 13 Jan 2020.
3. Panikkar R. Is the notion of Human rights a western concept? Diogenes 120; 1982.Avaliable at:http://www.derechoshumanos.unlp.edu.ar/assets/files/documentos/is-the-notion-of-human-rights-a-western-concept.pdf.Accessed 15 Jan 2020.
4. Peled-Raz M. Human rights in patient care and public health-a common ground. Public Health Rev. 2017; 38:29.
5. Cohen J & Ezer T. Human rights in patient care: a theoretical and practical framework. Health Hum Rights. 2013 Dec 12;15(2):7-19.
6. World Health organization. Patient Rights. Geneva: World Health Organization;2019. Available at: https://www.who.int/genomics/public/patientrights/en/ . Accessed 20 Jan 2020.
7. The free dictionary by Farlex.Patient rights. Pennyslvania:Farlex, Inc;2003-2019.Available from: https://medical-dictionary.thefreedictionary.com/patient+rights.Accessed 02 Jan 2020.
8. Townand D, Clemens T, Shaw D, Brand H, Nys H & Palm W.Patient's rights in the European Union, A mapping exercise. Luxembourg: European Union; 2016.

For peer review only - http://bmjopen.bmj.com/site/about/guidelines.xhtml

9. Méndez J. Report of the Special Rapporteur on torture and other cruel, inhuman or degrading treatment or punishment. New York: United Nations General Assembly;2013.

10. Reader WT & Gillespie A. Patient neglect in health care institutions: A systematic review and conceptual model. BMC Health Ser Res.2013;13:156.

11. Bell SK, Roche SD, Mueller A, et al Speaking up about care concerns in the ICU: patient and family experiences, attitudes and perceived barriers. BMJ Qual Saf. 2018; 27:928-936.

12. Tong WT, Lee YK, Ng CJ, Lee PY. Factors influencing implementation of a patient decision aid in a developing country: an exploratory study. Implement Sci. 2017 ;12(1):40.

13. Cubaka VK, Schriver M, Kayitare JB, Cotton P, Maindal HT, Nyirazinyoye L & Kallestrup P. 'He should feel your pain': Patient insights on patient-provider communication in Rwanda. Afr J Prim Health Care Fam Med. 2018:10(1).

14. Fochsen, G, Deshpande, K, & Thorson, A. (2006). Power Imbalance and Consumerism in the Doctor-Patient Relationship: Health Care Providers' Experiences of Patient Encounters in a Rural District in India. Qualitative Health Research, 16(9), 1236–51.

15. Henderson S. Power imbalance between nurses and patients: a potential inhibitor of partnership in care. J Clin Nurs. 2003 Jul;12(4):501-8.

16. Mead N, Bower P. Patient-centredness: a conceptual framework and review of the empirical literature. Soc Sci Med. 2000 Oct;51(7):1087-110.

17. Virona Gabriella M & Tarasenka N. Patient safety and rights: developing tools to support consumer health literacy. Regional Office for Europe. World Health Organization;2010.

For peer review only - http://bmjopen.bmj.com/site/about/guidelines.xhtml

18. Institute of Medicine. Crossing the Quality Chasm: A New Health System for the 21st Century. Washington DC: National Academy Press; 2001.

19. World Health Organization. Quality of care:A process for making strategic choices in health systems.Geneva:World Health Organization;2006.

20. Groene O. Patient centeredness and quality improvement efforts in hospitals: rationale, measurement, implementation. Int J Qual Health Care. 2011; 23(5): 531–37.

21. World Health Organization & UNICEF. Declaration of Asthana. Global conference on primary Health Care;2018. Available from: https://www.who.int/primary-health/conference-phc/declaration. Accessed Feb 20 2020.

22. Van Belle S, Boydell V, George AS, Brinkerhoff DW, Khosla R. Broadening understanding of accountability ecosystems in sexual and reproductive health and rights: A systematic review. PLoS One. 2018:13(5);e0196788.

23. Mirzoev T &Kane S.Key strategies to improve systems for managing patient complaints within health facilities- What can we learn from the existing literature?. Glob Health Action.2018;11:1458938.

24. Parliamentary and Health Service Ombudsman Impact study presentation of findings. London (United Kingdom): The Ombudsman;2010. Available at: www.ombudsman.org.uk/__data/assets/pdf_file/0013/4144/Impact-Study.pdf. Accessed Jan 02 2020.

25. Paterson R.The patients complaint system in Newzeland.Health Aff.2002;21(3):70-9.

26. Annual report 2009–2010. The Ombudsman: Quebec; 2010. Available at: www.protecteurducitoyen.qc.ca/en/cases-and-documentation/index.html. Accessed Feb 02 2020.

For peer review only - http://bmjopen.bmj.com/site/about/guidelines.xhtml

27. Francis R. Independent Inquiry into care provided by Mid Staffordshire NHS Foundation Trust January 2005 – March 2009. London: The Stationery Office;2010.

28. European Union Agency for fundamental rights. Inequalities and multiple discrimination in access to and quality health care.Luxembourg:European Union Agency for Fundamental Rights; 2013.

29. Glover ML, Sim M & Winters K. Perceived discrimination and reported trust and satisfaction with providers in African Americans. The Jackson heart study. Ethnicity & Disease.2017;27(3): 209-15.

30. Goerge S. Caste and Care: Is Indian Healthcare delivery system favourable for Dalits?Bangalore:The Institute of Social and Economic Change;2015.

31. Irudayam, A, J P Mangubhai & J G Lee. Dalit Women Speak Out: Violence against Dalit Women in India, Volume I and II. New Delhi: National Council for Dalit Human Rights;2006.

32. Open Society Foundations. Reports on the Patient Rights Violations in Krygystan. Newyork: Open Society Foundations;2008.

33. Bowser D & Hill K.Exploring evidence for disrespect and abuse in facility based childbirth.Report of a landscape analysis.Washington DC:USAID;2010.

34. Hammonds R, Hanefeld J & Ooms G. Accountablity as a driver of heath equity.Denmark: WHO Regional Office for Europe;2019.

35. Open Society Foundations. International/European framework for protection of human rights in patient care. Public Health Fact Sheet.Newyork: Open Society Foundations;2013.Available at: https://www.opensocietyfoundations.org/publications/international-european-framework-protection-human-rights-patient-care. Accessed Feb 19 2020.

For peer review only - http://bmjopen.bmj.com/site/about/guidelines.xhtml

36. National Human Rights Commission of India. Draft of patient rights charter. NewDelhi: Ministry of Health and Family Welfare;2018.Available at: https://mohfw.gov.in/newshighlights/draft-patient-charter-prepared-national-human-rights-commission.Accessed Feb 08 2020.

37. Department of Quality Assurance. Ministry of Health. Republic of Uganda. Patient's Charter:Uganda; Republic of Uganda;2009.Available at: http://cphl.go.ug/sites/default/files/library resources/PATIENTS%27%20CHARTER.pdf. Accessed Jan 22 2020.

38. Boudioni M, McLaren S & Lister G. A critical analysis of national policies, systems, and structures of patient empowerment in England and Greece. Patient Prefer Adherence. 2017; 11:1657–69.

39. Ontario Ministry of Health Care. Patients first :Action Plan for health care. Ontario: Ministry of long term care;2008. Available at: http://www.health.gov.on.ca/en/ms/ecfa/healthy_change/faqs_patientombudsman.aspx. Accessed Jan 03 2020

40. Shukla A, More A & Marathe S. Making private health care accountable:Mobilizing civil society and ethical doctors in India.Institute of Development Studies Bulletin.2018;49(2): 129-46.

41. Open Society Foundations.Strategic litigation impact:Insights from global experience.Newyork:Open Society Foundation;2018.

42. Unnikrishnan B, Trivedi D, Kanchan T, Rekha T, Mithra P, Kumar N, Kulkarni V, Holla R &Talish M. Patients' Awareness About Their Rights: A Study from Coastal South India. Sci Eng Ethics. 2017 Feb;23(1):203-14.

For peer review only - http://bmjopen.bmj.com/site/about/guidelines.xhtml

43. Agrawal U, D'Souza BC & Seetharam AM. Awareness of Patients' Rights among patients of a Tertiary Care Teaching Hospital- A Cross-sectional Study. J Clin Diagn Res. 2017 ;11(9): IC01-IC06.

44. Kagoya HR, Kibuule D, Mitonga-Kabwebwe H, Ekirapa-Kiracho E, Ssempebwa JC. Awareness of, responsiveness to and practice of patients' rights at Uganda's national referral hospital. Afr J Prim Health Care Fam Med. 2013;5(1):491. doi:10.4102/phcfm.v5i1.491

45. Abedi G, Shojaee J, Moosazadeh M, et al. Awareness and Observance of Patient Rights from the Perspective of Iranian Patients: A Systematic Review and Meta-Analysis. Iran J Med Sci. 2017;42(3):227–34.

46. Brinkerhoff DW, Bossert TJ. Health Governance: Concepts, Experience and Programming Options. Health Systems 2020 Policy Brief. Health Systems 2020.US:USAID;2008.

47. Institute of Governance. Definition of Governance. Canada: Institute of Governance; 2020.Available at https://iog.ca/what-is-governance/. Accessed Jul 18 2020.

48. Abimbola S, Negin J, Stephen J & Martiniuk A. Towards people centered health systems: multilevel framework for analyzing primary health care governance in low & middle income countries. Health Policy Plan. 2014; 29:ii29-ii39.

49. Marks G & Hooghes L. Themes and issues in multi-level governance. in Bache, I & Flinders, M (Editors). Multi-level governance. New York: Oxford University Press; 2004.

50. Gregoria DM, Fatorelli L, Paavola J, Locatelli B, Pramova E, Nurrochmat RD, May HP….& Kusumadewi DS. Multilevel governance and power in climate change policy networks. Global Environmental Change.2019;54:64-77.

For peer review only - http://bmjopen.bmj.com/site/about/guidelines.xhtml

51. Touati N, Maillet L, Paquette AM ,Denis DJ & Rodriguez C. Understanding Multilevel Governance Processes through Complexity Theory: An Empirical Case Study of the Quebec Health-Care System. International Journal of Public Administration. 42:3;205-17.

52. Ongaro E, Massey A, Holzer M & Wayenberg E.Governance and intergovernmental relations in the European Union and United States : theoretical perspectives. UK: Edward Elgar Publishing Limited;2010.

53. Daniell KA & Kay A. Multi-level governance: Conceptual challenges and case studies from Australia. Canberra:The Australian National University Press Canberra;2017.

54. Munn Z, Peters DJM, Stern C, Tufanaru C, McArthur A & Aromataris E. Systematic review or scoping review? Guidance for authors when choosing between a systematic or scoping review approach. BMC Med Res Methodol.2018;18:143.

55. Tricco, AC, Lillie, E, Zarin, W, O'Brien, KK, Colquhoun, H, Levac, D, Moher, D, Peters, MD, Horsley, T, Weeks, L, Hempel, S et al. PRISMA extension for scoping reviews (PRISMA-ScR): checklist and explanation. Ann Intern Med. 2018;169(7):467-73.

56. Dixon-Woods M, Cavers D, Agarwal S, Annandale E,Arthur A, Harvey J…..& Sutton JA. Conducting a critical interpretive synthesis of the literature on access to health care by vulnerable groups. BMC Med Res Methodol.2006;6:35.

57. Schaaf M & Topp MS. A critical interpretive synthesis of informal payments in maternal health care.Health Policy Plan.2019;34:216-29.

58. Wilson GM, Ellen EM, Lavis NJ, Grimsahw MJ, Moa A K,Shemer J et al. Processes, contexts and rationale for disinvestment: a protocol for a critical interpretive synthesis.Syst Rev.2014;3:143.

For peer review only

59. Mold A. Patient's rights & the National Health Service in Britain1960s-1098s. Am J Public Health.2012;102(11): 2030-37.

60. Grindle SM &Thomas WJ. Public choices and policy change. The political economy of reform in developing countries. Baltimore: John Hopkins University Press; 1991.

61. Sabatier P, Mazmanian D. The implementation of regulatory policy: A framework of analysis. Davis: Institute of Governmental Affairs; 1979.

62. Hill M, Hupe P. In: Aguilera , editor. Implementing Public Policy. 3rd ed. London: Sage Publications; 2014.

63. Young OR. The institutional dimension of environmental change: fit, interplay, and scale. Cambridge, Massachusetts: MIT Press;2002.

64. Bacchi. C. Problematization in health policy:Questioning how "problems" are constituted in policies.SAGE Open.2016;1-16.

65. Thaler HR & Sunstein RC.Nudge Improving decisions about health, wealth & Happiness.Yale University Press:New Haven& London;2008.

66. Giusti D, Criel B & Bethune DX.Viewpoint:public versus private health care debate beyond the slogans. Health Policy Plan.1997;12(3):193-98.

67. Hannes K. Chapter 4: Critical appraisal of qualitative research. In: Noyes J, Booth A, Hannes K, Harris J, Lewin S, Lockwood C(editors), Supplementary guidance for inclusion of qualitative research in Cochare systematic reviews of interventions.Version 1(updated August 2011). Cochare Colloboration Qualitataive Methods Group; 2011.

68. Ciliska D, Miccouci S, Dobbins M & Thoas HP.Effective Public Health Practice Project. Quality assessment tool for quantitative studies. Hamilton, on: Effective Public Health Practice Project;1998. Available at: https://merst.ca/ephpp/. Acessed Feb 03 2020.

Figure legends

For peer review only - http://bmjopen.bmj.com/site/about/guidelines.xhtml

Figure 1:The three main and the two cross cutting categories of patient rights.

Source:Townend et al, 2016.

Figure 2:The initial conceptual framework on the implementation of patient rights in health facilities from a multilevel governance perspective

Figure 3:Article selection and analysis algorithm

Supplementary files

Supplementary file 1

PRISMA checklist

Supplementary file 2

Detailed search strategy in PubMed database

Supplementary file 3

Quality appraisal criteria for qualitative studies

Suppplementary file 4

Quality appraisal criteria for quantitative studies

Supplementary file 5

Data extraction sheet

Authors contribution

MP and SVB led the conceptualization and the design of the protocol. MP drafted the manuscript. All authors (MP,SVB,BC,NE,AK,PBN and PNS) provided inputs , have read and approved this manuscript.

For peer review only

Funding statement

This work is supported by the PhD fellowship offered to Meena Putturaj by the Institute of Tropical Medicine, Antwerp, Directorate of Development Cooperation, Belgium.The time and the contributions of PNS for this project is supported by the Wellcome Trust/DBT India Alliance Intermediate Clinical and Public Health Research Fellowship awarded to him [IA/CPHI/16/1/502648].

Competing interests

The authors declare that they have no competing interests. The funders do not have any role in the design and the outcomes of this research project.

For peer review only

For peer review only - http://bmjopen.bmj.com/site/about/guidelines.xhtml

Basic individual rights	Social rights	Consumer-based rights
• Informed consent<br>• Privacy – confidentiality<br>• Access to medical record<br><br>•***Information*** about one's health and treatment options<br><br>•***Procedural*** (complain, redress, participation)	•Access to health care<br>•Equal treatment<br><br>•***Information*** on rights and entitlements (including the basket of care)<br><br>•***Procedural*** (complain, redress, participation	•Safe and timely treatment •Choice - Second opinion<br><br>•***Information*** on providers •***Procedural*** (complain, redress, participation)

Fig 1:The three main and the two cross cutting categories of patient rights

Source: Townend et al, 2016

For peer review only

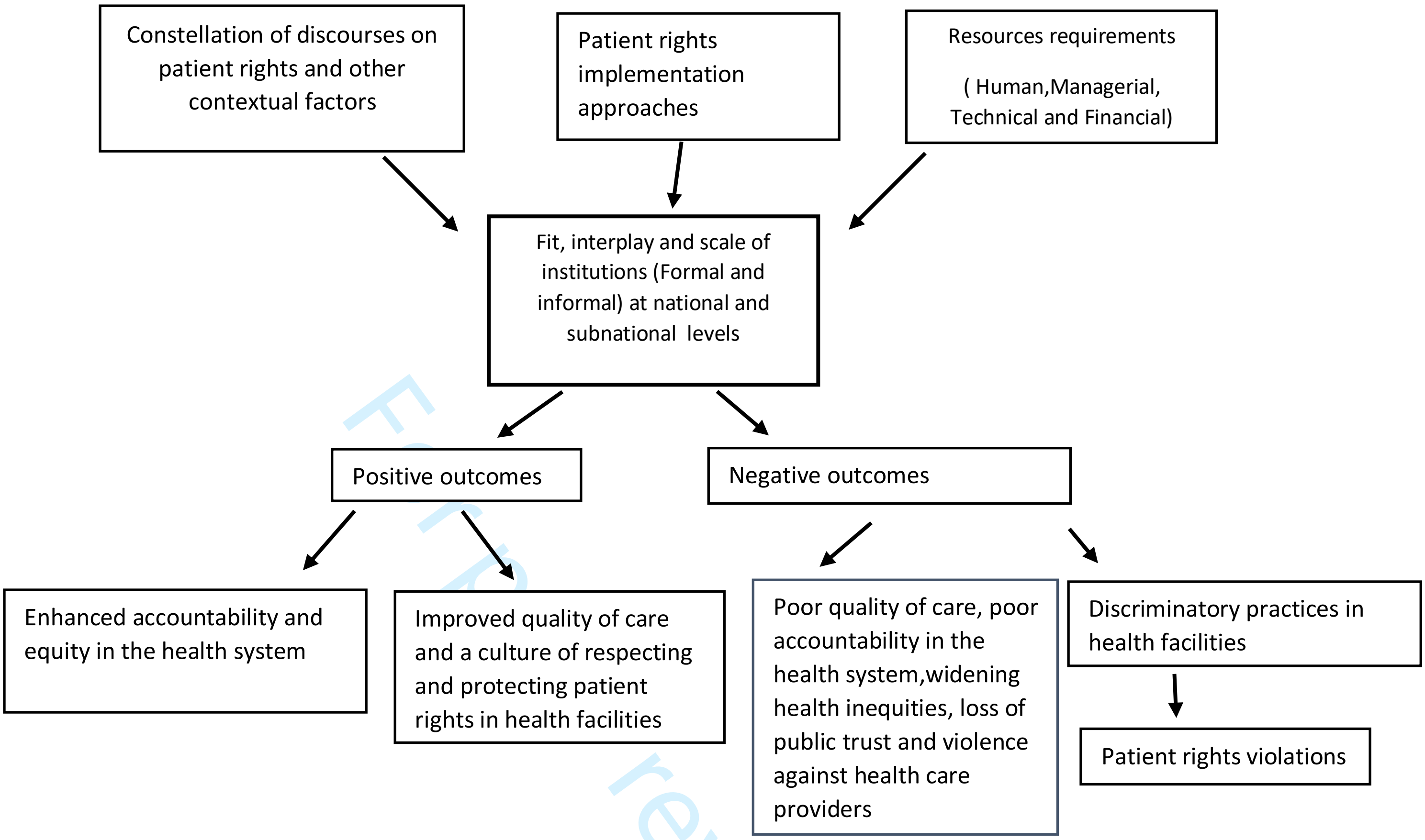

Fig 2:The initial conceptual framework on the implementation of patient rights in health facilities from a multi-level governance perspective

For peer review only - http://bmjopen.bmj.com/site/about/guidelines.xhtml

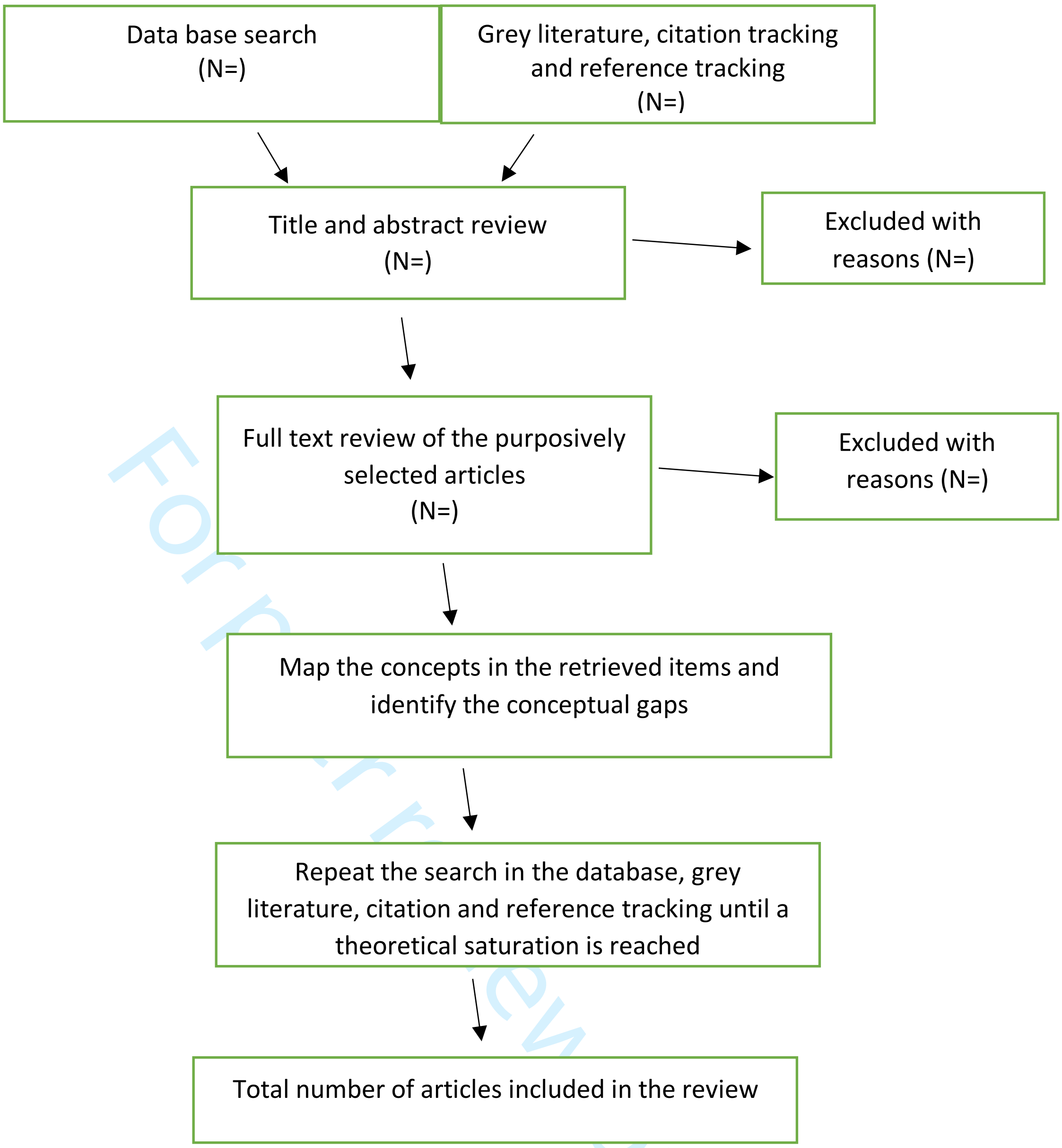

Fig3 : Article selection and analysis algorithm

For peer review only - http://bmjopen.bmj.com/site/about/guidelines.xhtml

Preferred Reporting Items for Systematic reviews and Meta-Analyses extension for Scoping Reviews (PRISMA-ScR) Checklist

SECTION	ITEM	PRISMA-ScR CHECKLIST ITEM	REPORTED ON PAGE #
**TITLE**			
Title	1	Identify the report as a scoping review.	2
**ABSTRACT**			
Structured summary	2	Provide a structured summary that includes (as applicable): background, objectives, eligibility criteria, sources of evidence, charting methods, results, and conclusions that relate to the review questions and objectives.	2,3
**INTRODUCTION**			
Rationale	3	Describe the rationale for the review in the context of what is already known. Explain why the review questions/objectives lend themselves to a scoping review approach.	4-13
Objectives	4	Provide an explicit statement of the questions and objectives being addressed with reference to their key elements (e.g., population or participants, concepts, and context) or other relevant key elements used to conceptualize the review questions and/or objectives.	13
**METHODS**			
Protocol and registration	5	Indicate whether a review protocol exists; state if and where it can be accessed (e.g., a Web address); and if available, provide registration information, including the registration number.	24
Eligibility criteria	6	Specify characteristics of the sources of evidence used as eligibility criteria (e.g., years considered, language, and publication status), and provide a rationale.	18-21
Information sources*	7	Describe all information sources in the search (e.g., databases with dates of coverage and contact with authors to identify additional sources), as well as the date the most recent search was executed.	18-21
Search	8	Present the full electronic search strategy for at least 1 database, including any limits used, such that it could be repeated.	18-21, Supplementary file-2
Selection of sources of evidence†	9	State the process for selecting sources of evidence (i.e., screening and eligibility) included in the scoping review.	18-21, Figure 3
Data charting process‡	10	Describe the methods of charting data from the included sources of evidence (e.g., calibrated forms or forms that have been tested by the team before their use, and whether data charting was done independently or in duplicate) and any processes for obtaining and confirming data from investigators.	14-18, 22,23
Data items	11	List and define all variables for which data were sought and any assumptions and simplifications made.	Figure 2, page no 14-18 &22-23, Supplementary file 5
Critical appraisal of individual sources of evidence§	12	If done, provide a rationale for conducting a critical appraisal of included sources of evidence; describe the methods used and how this information was used in any data synthesis (if appropriate).	22, supplementary file 3&4

St. Michael's
Inspired Care. Inspiring Science.

For peer review only - http://bmjopen.bmj.com/site/about/guidelines.xhtml

Synthesis of results	13	Describe the methods of handling and summarizing the data that were charted.	22,23

For peer review only

SECTION	ITEM	PRISMA-ScR CHECKLIST ITEM	REPORTED ON PAGE #
**RESULTS**			
Selection of sources of evidence	14	Give numbers of sources of evidence screened, assessed for eligibility, and included in the review, with reasons for exclusions at each stage, ideally using a flow diagram.	NA since this is a protocol
Characteristics of sources of evidence	15	For each source of evidence, present characteristics for which data were charted and provide the citations.	NA since this is a protocol
Critical appraisal within sources of evidence	16	If done, present data on critical appraisal of included sources of evidence (see item 12).	NA since this is a protocol
Results of individual sources of evidence	17	For each included source of evidence, present the relevant data that were charted that relate to the review questions and objectives.	NA since this is a protocol
Synthesis of results	18	Summarize and/or present the charting results as they relate to the review questions and objectives.	NA since this is a protocol
**DISCUSSION**			
Summary of evidence	19	Summarize the main results (including an overview of concepts, themes, and types of evidence available), link to the review questions and objectives, and consider the relevance to key groups.	NA since this is a protocol
Limitations	20	Discuss the limitations of the scoping review process.	
Conclusions	21	Provide a general interpretation of the results with respect to the review questions and objectives, as well as potential implications and/or next steps.	NA since this is a protocol
**FUNDING**			
Funding	22	Describe sources of funding for the included sources of evidence, as well as sources of funding for the scoping review. Describe the role of the funders of the scoping review.	

JBI = Joanna Briggs Institute; PRISMA-ScR = Preferred Reporting Items for Systematic reviews and Meta-Analyses extension for Scoping Reviews.
* Where *sources of evidence* (see second footnote) are compiled from, such as bibliographic databases, social media platforms, and Web sites.
† A more inclusive/heterogeneous term used to account for the different types of evidence or data sources (e.g., quantitative and/or qualitative research, expert opinion, and policy documents) that may be eligible in a scoping review as opposed to only studies. This is not to be confused with *information sources* (see first footnote).
‡ The frameworks by Arksey and O'Malley (6) and Levac and colleagues (7) and the JBI guidance (4, 5) refer to the process of data extraction in a scoping review as data charting.
§ The process of systematically examining research evidence to assess its validity, results, and relevance before using it to inform a decision. This term is used for items 12 and 19 instead of "risk of bias" (which is more applicable to systematic reviews of interventions) to include and acknowledge the various sources of evidence that may be used in a scoping review (e.g., quantitative and/or qualitative research, expert opinion, and policy document).

From: Tricco AC, Lillie E, Zarin W, O'Brien KK, Colquhoun H, Levac D, et al. PRISMA Extension for Scoping Reviews (PRISMA-ScR): Checklist and Explanation. Ann Intern Med. ;169:467–473. doi: 10.7326/M18-0850

For peer review only - http://bmjopen.bmj.com/site/about/guidelines.xhtml

Detailed search strategy in the PubMed database

Table 1: Key concepts and related free text terms for search in the database

	Key concept 1	Key concept 2	Key concept 3
	**Patient rights**	**Patient rights instruments**	**Person centered care**
**Related free text terms**	Patient bill of rights, patient rights charter, quality of health care, social(community) accountability in health, Patient accountability, patient safety, discrimination, health equity, equality, right to health, patient rights and ethics	Patient rights legislation, patient bill of rights, health ombudsman, Right to information act, health councils, patient welfare committees, patient grievance redressal systems/ committees, patient complaints system, patient advocates, parliamentary hearings, public hearing, public protests, strategic litigation, consumer forums, professional associations, social audit, audit bodies, patient suggestion box, court system for patient rights, human rights commissions, health care quality councils	Patient centered care, people centered care, patient autonomy, patient engagement, Patient participation, Patient empowerment

The free text terms for each of the key concepts mentioned in Table 1 were pilot tested in the PubMed database. Each free text terms were searched separately to see whether the search results are relevant. The results of the initial search in the PubMed database are shown in Table 2. Since the initial search yielded unmanageable number of records. The search terms were made more specific. Also, use of Boolean operators such as “AND” and application of “free text filter” yielded relevant and manageable number of records. Since the governance aspect (not a core medical topic) of the patient rights implementation is the focus in the review, it was expected that the key concepts of the review may not be well indexed in the PubMed database. Hence, the search field included “All fields”. The refined search strategy is shown in Table 3.

Table 2: Results of the pilot testing of the free text terms in the PubMed database

Free text terms	Search results
Patient* bill of rights	529
Community accountability	8128
Patient safety	157872
Health equity	24519
Right to health	66755
Equality	367726
Patient rights and ethics	33598
Quality of health care	7025620
Social accountability	36577
Patient rights legislation	31773
Health ombudsman	321
Right to information act	1952
Health councils	117769
Patient welfare committees	1149
Patient grievance redressal systems	1
Patient complaint system	7540
Patient advocates	30901
Parliamentary hearings	11
Strategic litigation	1501
Consumer forums	1025
Professional associations	74651
Social audit	3558
Audit bodies	433
Patient suggestion box	13669
Court system for patient rights	301
Human rights commission	2252
Health care quality councils	97728
Patient centered care	33517
People centered care	14100
Patient autonomy	21190
Patient engagement	66813
Patient participation	55600
Patient empowerment	58745

For peer review only

Table 3: Refined search strategy in the PubMed database

Data source	Search terms	Search fields	Filters	Search results
PubMed database	Patient bill of rights	All fields	Full text	253
	Patient bill of rights AND implementation	All fields	Full text	23
	Patient charter of rights	All fields	Full text	147
	Patient charter of rights AND implementation	All fields	Full text	22
	Health ombudsman	All fields	Full text	186
	Patient grievance redressal	All fields	Full text	3
	Patient complaints AND Patient rights	All fields	Full text	270
	complaints procedures AND patient rights	All fields	Full text	173
	Rights AND strategic litigation	All fields	Full text	951
	Patient rights AND Consumer forums	All fields	Full text	14
	Consumer Health Forums AND patient rights	All fields	Full text	12
	Quality Health care AND patient bill of rights	All fields	Full text	80

Similar steps will be followed in the Web of Science and Lexis Nexis Database as well. The filters, search fields and the search terms will be chosen based on what is feasible in those databases.

For peer review only

Quality appraisal criteria for the qualitative studies

Criteria	Study 1	Study 2	Study 3	Study …
Credibility				
Transferability				
Dependability				
Confirmability				
Overall quality	H/L/Judge			
Comments				

Note:

- To give more weight to studies that score high on quality
- Sensitivity analyses could be done to strike a balance between the methodological rigor and conceptual breadth and depth

For peer review only

QUALITY ASSESSMENT TOOL FOR QUANTITATIVE STUDIES

Effective Public Health Practice Project

COMPONENT RATINGS

A) SELECTION BIAS

(Q1) Are the individuals selected to participate in the study likely to be representative of the target population?

1 Very likely
2 Somewhat likely
3 Not likely
4 Can't tell

(Q2) What percentage of selected individuals agreed to participate?

1 80 - 100% agreement
2 60 – 79% agreement
3 less than 60% agreement
4 Not applicable
5 Can't tell

RATE THIS SECTION	STRONG	MODERATE	WEAK
See dictionary	1	2	3

B) STUDY DESIGN

Indicate the study design

1 Randomized controlled trial
2 Controlled clinical trial
3 Cohort analytic (two group pre + post)
4 Case-control
5 Cohort (one group pre + post (before and after))
6 Interrupted time series
7 Other specify ________________________________
8 Can't tell

Was the study described as randomized? If NO, go to Component C.

No Yes

If Yes, was the method of randomization described? (See dictionary)

No Yes

If Yes, was the method appropriate? (See dictionary)

No Yes

RATE THIS SECTION	STRONG	MODERATE	WEAK
**See dictionary**	1	2	3

For peer review only - http://bmjopen.bmj.com/site/about/guidelines.xhtml

C) CONFOUNDERS

(Q1) Were there important differences between groups prior to the intervention?

1 Yes
2 No
3 Can't tell

The following are examples of confounders:

1 Race
2 Sex
3 Marital status/family
4 Age
5 SES (income or class)
6 Education
7 Health status
8 Pre-intervention score on outcome measure

(Q2) If yes, indicate the percentage of relevant confounders that were controlled (either in the design (e.g. stratification, matching) or analysis)?

1 80 – 100% (most)
2 60 – 79% (some)
3 Less than 60% (few or none)
4 Can't Tell

RATE THIS SECTION	STRONG	MODERATE	WEAK
See dictionary	1	2	3

D) BLINDING

(Q1) Was (were) the outcome assessor(s) aware of the intervention or exposure status of participants?

1 Yes
2 No
3 Can't tell

(Q2) Were the study participants aware of the research question?

1 Yes
2 No
3 Can't tell

RATE THIS SECTION	STRONG	MODERATE	WEAK
See dictionary	1	2	3

For peer review only

E) DATA COLLECTION METHODS

(Q1) Were data collection tools shown to be valid?

1 Yes
2 No
3 Can't tell

(Q2) Were data collection tools shown to be reliable?

1 Yes
2 No
3 Can't tell

RATE THIS SECTION	STRONG	MODERATE	WEAK
See dictionary	1	2	3

For peer review only - http://bmjopen.bmj.com/site/about/guidelines.xhtml

F) WITHDRAWALS AND DROP-OUTS

(Q1) Were withdrawals and drop-outs reported in terms of numbers and/or reasons per group?
1 Yes
2 No
3 Can't tell
4 Not Applicable (i.e. one time surveys or interviews)

(Q2) Indicate the percentage of participants completing the study. (If the percentage differs by groups, record the lowest).
1 80 -100%
2 60 - 79%
3 less than 60%
4 Can't tell
5 Not Applicable (i.e. Retrospective case-control)

RATE THIS SECTION	STRONG	MODERATE	WEAK	
See dictionary	1	2	3	Not Applicable

G) INTERVENTION INTEGRITY

(Q1) What percentage of participants received the allocated intervention or exposure of interest?
1 80 -100%
2 60 - 79%
3 less than 60%
4 Can't tell

(Q2) Was the consistency of the intervention measured?
1 Yes
2 No
3 Can't tell

(Q3) Is it likely that subjects received an unintended intervention (contamination or co-intervention) that may influence the results?
4 Yes
5 No
6 Can't tell

H) ANALYSES

(Q1) Indicate the unit of allocation (circle one)
community organization/institution practice/office individual

(Q2) Indicate the unit of analysis (circle one)
community organization/institution practice/office individual

(Q3) Are the statistical methods appropriate for the study design?
1 Yes
2 No
3 Can't tell

(Q4) Is the analysis performed by intervention allocation status (i.e. intention to treat) rather than the actual intervention received?
1 Yes
2 No
3 Can't tell

For peer review only - http://bmjopen.bmj.com/site/about/guidelines.xhtml

GLOBAL RATING

COMPONENT RATINGS

Please transcribe the information from the gray boxes on pages 1-4 onto this page. See dictionary on how to rate this section.

**A**	**SELECTION BIAS**	**STRONG**	**MODERATE**	**WEAK**	
		1	2	3	
**B**	**STUDY DESIGN**	**STRONG**	**MODERATE**	**WEAK**	
		1	2	3	
**C**	**CONFOUNDERS**	**STRONG**	**MODERATE**	**WEAK**	
		1	2	3	
**D**	**BLINDING**	**STRONG**	**MODERATE**	**WEAK**	
		1	2	3	
**E**	**DATA COLLECTION METHOD**	**STRONG**	**MODERATE**	**WEAK**	
		1	2	3	
**F**	**WITHDRAWALS AND DROPOUTS**	**STRONG**	**MODERATE**	**WEAK**	
		1	2	3	Not Applicable

GLOBAL RATING FOR THIS PAPER (circle one):

1 STRONG (no WEAK ratings)
2 MODERATE (one WEAK rating)
3 WEAK (two or more WEAK ratings)

With both reviewers discussing the ratings:

Is there a discrepancy between the two reviewers with respect to the component (A-F) ratings?

No Yes

If yes, indicate the reason for the discrepancy

1 Oversight
2 Differences in interpretation of criteria
3 Differences in interpretation of study

Final decision of both reviewers (circle one):

1 STRONG
2 MODERATE
3 WEAK

For peer review only - http://bmjopen.bmj.com/site/about/guidelines.xhtml

Data extraction sheet

	Authors	Author affiliations	Funding for the study	Year of publication	Article type	Study design (for research papers)	Study setting	Country
Record 1								
Record 2								
Record 3								
….								

For peer review only

BMJ Open

Towards a multi-level governance framework on the implementation of patient rights in health facilities:a protocol for a systematic scoping review

Journal:	*BMJ Open*
Manuscript ID	bmjopen-2020-038927.R2
Article Type:	Protocol
Date Submitted by the Author:	24-Aug-2020
Complete List of Authors:	PUTTURAJ, MEENA; The University of Trans-disciplinary Health Sciences and Technology, Centre for local health traditions and policy<br>Van Belle, Sara; Department of Public Health., Institute of Tropical Medicine<br>Criel, Bart; Institute of Tropical Medicine, Department of Public Health<br>Engel, Nora; Maastricht University,<br>Krumeich, Anja; Maastricht University, Department of Health, Ethics and Society<br>B Nagendrappa, Prakash; The University of Trans-disciplinary Health Sciences and Technology, Centre for local health traditions and policy<br>Prashanth, NS; Institute of Public Health,
**Primary Subject Heading**:	Health policy
Secondary Subject Heading:	Health services research, Patient-centred medicine, Public health, Qualitative research
Keywords:	Health policy < HEALTH SERVICES ADMINISTRATION & MANAGEMENT, PUBLIC HEALTH, MEDICAL ETHICS, QUALITATIVE RESEARCH

SCHOLARONE™
Manuscripts

I, the Submitting Author has the right to grant and does grant on behalf of all authors of the Work (as defined in the below author licence), an exclusive licence and/or a non-exclusive licence for contributions from authors who are: i) UK Crown employees; ii) where BMJ has agreed a CC-BY licence shall apply, and/or iii) in accordance with the terms applicable for US Federal Government officers or employees acting as part of their official duties; on a worldwide, perpetual, irrevocable, royalty-free basis to BMJ Publishing Group Ltd ("BMJ") its licensees and where the relevant Journal is co-owned by BMJ to the co-owners of the Journal, to publish the Work in this journal and any other BMJ products and to exploit all rights, as set out in our licence.

The Submitting Author accepts and understands that any supply made under these terms is made by BMJ to the Submitting Author unless you are acting as an employee on behalf of your employer or a postgraduate student of an affiliated institution which is paying any applicable article publishing charge ("APC") for Open Access articles. Where the Submitting Author wishes to make the Work available on an Open Access basis (and intends to pay the relevant APC), the terms of reuse of such Open Access shall be governed by a Creative Commons licence – details of these licences and which Creative Commons licence will apply to this Work are set out in our licence referred to above.

Other than as permitted in any relevant BMJ Author's Self Archiving Policies, I confirm this Work has not been accepted for publication elsewhere, is not being considered for publication elsewhere and does not duplicate material already published. I confirm all authors consent to publication of this Work and authorise the granting of this licence.

For peer review only

Towards a multi-level governance framework on the implementation of patient rights in health facilities:a protocol for a systematic scoping review

Meena Putturaj[1,2,3,4], Sara Van Belle [1], Bart Criel[1], Nora Engel[2], Anja Krumeich[2], Prakash BN[3] & Prashanth NS [4]

Institutions

1.Institute of Tropical Medicine, Antwerp, Belgium

2.Maastricht University, Netherlands

3.The University of Transdiscplinary Health Sciences and Technology, Bengaluru, India

4.Institute of Public Health, Bengaluru,India

Authors and Author's affiliations

1. Meena Putturaj *(Corresponding author)*[1,2,3,4]
Centre for Local Health Traditions and Policy
The University of Trans -Disciplinary Health Sciences and Technology
74/2, Post Attur via Yelahanka, Jarakabande Kaval, Bengaluru, Karnataka 560064, India
meenaputturaj@gmail.com

2.Dr.Sara Van Belle[1]
svanbelle@itg.be

3.Dr.Bart Criel[1]
Bcriel@itg.be

4.Dr. Nora Engel[2]
n.engel@maastrichtuniversity.nl

5. Dr. Anja Krumeich[2]
a.krumeich@maastrichtuniversity.nl

6.Dr. Prakash BN[3]
bn.prakash@tdu.edu.in

7.Dr. Prashanth Nuggehalli Srinivas[4].
prashanthns@iphindia.org

Word count: 5346 excluding the title page, abstract, references, figures and tables

For peer review only - http://bmjopen.bmj.com/site/about/guidelines.xhtml

Towards a multi-level governance framework on the implementation of patient rights in health facilities:a protocol for a systematic scoping review

Abstract

Introduction

Patient rights are “those rights that are attributed to a person seeking health care”. Patient rights have implications for quality of health care and acts as a key accountability tool. It can galvanize structural improvements in the health system and reinforces ethical health care. States are duty bound to respect, protect and promote patient rights. The rhetoric on patient rights is burgeoning across the globe. With changing modes of governance arrangements, a number of state and non-state actors and institutions at various levels play a role in the design and implementation of (patient rights)policies. However, there is limited understanding on the multilevel institutional mechanisms for patient rights implementation in health facilities. We attempt to fill this gap by critically analyzing the available scholarship on patient rights through a critical interpretive synthesis approach in a systematic scoping review.

Methods

The review question is “how do the multilevel actors, institutional structures, processes interact and influence the patient rights implementation in health care facilities? How do they work at what level and in which contexts?”Three databases PubMed, Lexis Nexis and Web of Science will be systematically searched until 30th April 2020, for empirical and non-empirical literature in English from both LMIC and HIC. Targeted search will be performed in grey literature and through citation and reference tracking of key records. Using the critical

For peer review only - http://bmjopen.bmj.com/site/about/guidelines.xhtml

interpretive synthesis approach , a multilevel governance framework on the implementation of patient rights in health facilities which is grounded in the data will be developed.

Ethics & Dissemination

The review uses published literature hence ethics approval is not required. The findings of the review will be published in a peer reviewed journal.

Registration

This review is registered with PROSPERO and the registration ID is CRD2020176939.

Key words

Patient rights implementation, critical interpretive synthesis, multi-level governance

Strengths and limitations of this study

- The proposed review will deliver an explanatory framework on the patient rights implementation in health facilities from a multilevel governance perspective
- The use of literature from multiple disciplines provide a nuanced understanding of the contested concepts such as the patient rights that are complex involving many stakeholders and tradeoffs
- The Critical Interpretive Synthesis (CIS) approach to synthesize the evidence is a perfect fit to understand the nature of complex fuzzy concepts such as a patient rights implementation as it enables the use of data emerging from heterogenic disciplines and methodologies

For peer review only - http://bmjopen.bmj.com/site/about/guidelines.xhtml

- The application of multilevel governance as the analitycal lens is useful to study what vertical and horizontal levels and in what roles various state, market, and civil society actors and institutions interact for the implementation of patient rights.
- Considering the time frame available for the review, the following are not within the scope of this review 1.Patient rights implementation by the informal health care providers 2. Patient rights concerning mental health disorders, disabilities, genetic screening, euthanasia, advance medical directives and health research as they merit specific considerations 3. Patient responsibilities/obligations 4. International mechanisms for patient rights protection(e.g. United Nations Human Rights Council).

Introduction

Human rights are believed to be the rights and freedoms that are inherent to every person in this world without any bias. Formally in 1948, in the aftermath of the World War II, the "UN Declaration of Human Rights" laid the foundation for defining fundamental rights shared by every human being (e.g. right to life, liberty and security, right to equality, freedom from torture etc.). These rights proclaimed in the Declaration were adopted by the UN General Assembly and ever since considered as the norm(s) by all nations for all people [1]. However, cultural relativists and post-colonial critics argue that the universal human rights doctrine in a multicultural society is an imposition of Western philosophy, values and norms on non-Western cultures[2]. Raimon Panikkar, an Indian philosopher, argued that despite human rights being a Western notion, it can still act as an instrument for social justice in non-Western settings e.g. India. However, a cross-cultural philosophical approach to human rights is vital [3].

For peer review only

Embedded within the broader perspective of human rights is the notion of “patients rights”[4-6]. Patient rights refer to “those rights attributed to a person seeking health care”[7]. It is the application of human rights principles in a health care setting - the location where health care providers offer services to the people.

Townend and his colleagues [8] **(see Figure 1)** identified five main categories of patient rights 1) *individual rights* (certain liberties guaranteed for an individual person) 2) *consumer* (one who purchases the economic services and commodities) *based rights* 3) *social rights* (emerging from social contract e.g. between state and the citizens) 4*) procedural patient rights* 5) *informational patient rights.* The last two categories (4 &5) are cross-cutting and help to enforce the first three categories of patient rights.

However, the categories mentioned above are not mutually exclusive. For example, the right to informed consent (individual right) is connected to right to information (procedural right). Likewise, the right to complain depends on the right to access medical files in order to prove that the right to informed consent was violated [8].

Why focus on patient rights’?

Because of structural inequalities such as power imbalances between the care seeking individuals and providers and other social inequities related to income gap, gender inequality, social class and stigma associated with health conditions e.g. HIV, tuberculosis, mental disorders etc, health care settings are specifically vulnerable to patient rights violations[9]. Most often, the vulnerability to patient rights violations emerges from a complex mixture of organizational and systemic factors (e.g. lack of human resources,for providing respectful care) shaping the health care provider-person encounter[10]. There is considerable evidence regarding the prevalence of paternalism in the medical

For peer review only - http://bmjopen.bmj.com/site/about/guidelines.xhtml

profession[11-15]. This has given rise to the scholarly work on people-centered care models emphasizing patient autonomy and joint decision-making in the health care processes [16].

The importance of patient rights could be gleaned from multiple perspectives:

i) From the perspective of patient safety

In a traditional hierarchical relationship, patients might refrain from having an open communication with health care providers or the health care providers may not provide in-depth information to people seeking care . This lack of open communication might deter patients from participating in the discussions related to the prevention of health care related errors and thus have implications for patient safety. Patient rights provide a language to empower and engage the care seeking individuals in the processes of delivering health care[17].

ii) From the perspective of quality of health care

The six dimensions of quality health care are safety, timeliness/accessibility, patient centeredness/acceptability, equity, efficiency and effectiveness [18,19]. Most of the components of patient rights align with the key dimensions of quality health care. For instance, scholars have reiterated the need for respecting the patient rights as a tangible entry point for achieving people- centered care[20,21].Thus, the notion of patient rights is in consonance with the concepts of quality of health care and people-centered care.

Iii) From the health system perspective

Furthermore, patient rights might galvanize improvements at health system level by infusing accountability through oversight mechanisms [22]. Efficient and functional patient complaints and grievance redressal systems offer critical lessons for the structural

For peer review only - http://bmjopen.bmj.com/site/about/guidelines.xhtml

improvement of the health system[23].Evidence from UK, New-Zealand and Canada show that the recommendations of patient rights and safety ombudsmen have the potential to influence policy makers at the highest level [24-26].

iv) From an ethical perspective

Patient rights are deeply interwoven with the four core principles of medical/ health care ethics i.e. beneficence (do good), non - maleficence (do no harm), justice and autonomy [4]. Patient rights and the principles of medical ethics complement each other. Any deviation of the health care professional's practice from medical ethics is often equated with the violation of patient rights. See for instance, the Mid-Staffordshire National Health System (NHS) scandal in UK which revealed the preventable deaths of patients because of medical negligence by the health care staff[27], evoked concerns on the breach of patient's dignity and their right to quality care in the NHS[9].

v) From the health equity perspective

Research studies from both high-income contexts (e.g. USA, Europe)[28,29] and LMICs (e.g. India, Kyrgyzstan) [30-32] reveal the existence of discriminatory practices towards people receiving care. The discrimination could be based on single grounds (e.g. HIV status of a person, a migrant in Europe, sex worker etc.) or on the complex intersections of multiple grounds (e.g. age, gender, ethnicity, socio-economic status, disability etc.). The growing global consensus for respectful maternity care is a case in point that illustrates the magnitude of the patient rights violations experienced by the vulnerable populations (childbearing women) at all levels of health care[33]. It is acknowledged that strengthening accountability in the health system will help tackle health inequities[34]. Patient rights could serve as a health system accountability tool [22,28].

For peer review only - http://bmjopen.bmj.com/site/about/guidelines.xhtml

Rationale

The social, cultural and the policy context shape the design and the implementation of patient rights resulting in variations across the settings [6]. For example, on comparing the content of the patient rights charter agreed by the European Union[35] with that of the Indian[36] and the Ugandan patient bill of rights[37], the European Union patient rights charter had included “right to innovation” “right to avoid unnecessary suffering and pain” “right to personalized treatment” “right to respect patients time”. These rights were not mentioned in the Indian and the Ugandan bills of patient rights. The levers to enforce the patient rights also vary between countries. In countries like UK[38], Netherlands[8], Sweden[8], Canada[39] patient rights are enshrined in law and there are separate statutory bodies. For example, health ombudsmen (i.e. an independent public authority who deals with citizens’ complaints against the public/private institutions) are available to people who wish to complain when they feel that their rights are not respected. The National Health System of UK also provides assistance to the care seeking individuals to navigate the grievance redressal system[38]. Inadequate institutional mechanisms to respect, protect and fulfill human (patients) rights in certain Lower middle -income countries (LMIC) (e.g. India, South Africa, Brazil) are increasingly compensated by citizen action and strategic litigation steered by civil society organizations[22,40,41].

Patient rights movements and civil society organizations arguing for improved accountability in health care are gathering momentum across the globe and more so in LMICs [40]. Although the rhetoric on patient rights is burgeoning, empirical research in this area is nascent and most of the existing research studies [42-45] limit themselves to studying awareness of patient rights. Also, the available frameworks in the literature identify the

For peer review only

typology of patients rights [8] and a few other attempt to situate the patients rights' within the larger human rights framework [4]. According to the human rights law, states are duty-bound to protect human rights vis-a-vis patient rights[1]. Often in LMICs, the failure of the health care related policies is attributed to ineffective and inefficient governance [46].There is limited scholarly work that shed light on the influence of the governance arrangements on patient rights implementation.

We attempt to fill this gap by critically reviewing the existing scholarship on patient rights and come up with a framework on the implementation of patient rights from a multilevel governance perspective. The concept of governance is difficult to define. Most of the definitions emphasize *power, authority and accountability* as the key aspects of governance[47]. Governance is not anymore a role played only by the governments. A broad range of actors and institutions (both state and non-state) such as the civil society, media, professional groups engage at different levels of system depending on their power and interest in the policy problem [48]. The emergence of concepts such as the multi level governance, multi tiered governance, polycentric governance, multi perspectival governance, network governance indicate the diffusion of authority from the centre to more regional and local levels thus bringing governance systems close to the communities. Multi level governance also highlights the proliferation of non-state actors in the public policy processes. In simple terms, multi-level governance refers to the dispersion of governance across multiple jurisdictions and multiple actors (international, national, subnational). The idea of multilevel governance was first applied to explain the European Union politics[49]. The concept was later used to explore the governance of common pool resources e.g. land use, irrigation systems, renewable energy and community fisheries as well as in the analysis of health care system and organization of primary health care

For peer review only - http://bmjopen.bmj.com/site/about/guidelines.xhtml

services[48,50]. There are two varieties. In type I multi level governance the jurisdictions are defined clearly by the territorial boundaries and the functions of governance are confined to these territorial boundaries e.g.Court system. An alternative form is the type II multilevel governance where the jurisdictions often overlap and operate at huge territorial scales depending on the nature of the policy problem addressed. e.g. monitoring water quality of a particular river, resolve conflicts pertaining to common pool resources etc[49]. Divay and Paquin (2013) argued that multilevel governance is not merely restricted to intergovernmental relations rather it involves flexibility in role sharing between various stakeholders (both state and non state) at multiple levels in the governance processes [as cited in 51]. For our systematic scoping review we consider the conceptualization of multi level governance as the one that is characterized by “the study of the crossroads of the vertical (intergovernmental) and horizontal (state-society) dimensions”[52]. Also, policy design and implementation is a matter of continuous negotiation and cross level interactions among the diverse set of actors within the nested jurisdictions[50,53]. Power relations drive the cross level interactions in the multilevel governance systems [50]. We propose that multilevel governance as the analytical lens is useful to study what vertical and horizontal levels in the governance system and in what roles various state, market, and civil society actors interact for the implementation of patient rights.

In the field of evidence synthesis, systematic scoping reviews are considered as an appropriate approach to answer complex questions where systematic reviews are not possible, or do not produce the required (conceptually rich) information. Unlike the systematic reviews, systematic scoping reviews are applied for investigating broad questions that are interdisciplinary in nature but in a systematic manner and does not involve strict

For peer review only - http://bmjopen.bmj.com/site/about/guidelines.xhtml

exclusion criteria for the studies [54]. The proposed review uses the PRISMA-P guidelines[55] for reporting systematic reviews**(see supplementary file 1).**

Further in this systematic scoping review, considering the limited theoretical development in the area of patient rights implementation, the critical interpretive synthesis(CIS) [56] will be used as a technique for searching and synthesizing the evidence. The CIS approach offers guidance to marshall evidence to answer complex fuzzy questions that often cut across multiple disciplines as it allows to

- use diverse body of evidence from varied forms of data sources,disciplines and methodologies
- modify the review question in response to the search results and findings of the previously retrieved items
- use literature from the adjacent fields relevant to the emerging nature of the review question
- use a pragmatic approach to identify potentially relevant and conceptually sound literature that would contribute for theory building or an explanatory framework
- critique the ways of the problematization of the phenomena under study in the literature and explore the underlying assumptions on which the solution to the social problems are constituted

We have shown the salient features of CIS approach in **box 1**[56]. CIS is successfully applied in the field of health systems to explore areas such as informal payments in maternal health care[57], access to health care[56], overuse of health services[58]. The concept of patient rights itself is a subject of multiple interpretations, partially owing to the confusion that exists on who is claiming these rights; is it the patient or consumer or

For peer review only - http://bmjopen.bmj.com/site/about/guidelines.xhtml

For peer review only

citizen?[59].The interrelatedness of patient rights with health system accountability, quality of health care, health care ethics, and equity issues further makes it difficult to define clearly the patient rights conceptually. Further attempting to explore the ” implementation” aspect of the patient rights adds another layer in the complexity owing to its overlapping with the related fields such as governance and health policy processes. Since the focus of the CIS approach is towards achieving conceptual clarity and theory development, it might help to search, retrieve and synthesize from the literature on patient rights implementation that is diverse and heterogenic in terms of methods and disciplines.

Box 1: Salient features of CIS approach

- The review question serves as a compass rather than an anchor and is therefore revised and refined during the review process
- The aim is to develop synthesizing argument which could take the form of a plausible theoretical framework reflecting the network of constructs grounded in the evidence
- The inductive and iterative nature of the CIS approach demands several cycles of searching, sampling, critiquing and analyzing the evidence until a theoretical saturation is reached
- The subsequent cycles of searching, sampling, critiquing and analyzing the evidence is informed by the emerging theoretical framework
- The priority for selecting the evidence is “signal”(i.e.likely relevance to answer the review question) over “noise”(the inverse of methodological quality). However, formal quality appraisal techniques could be applied for individual papers. The aim is to purposively select papers that are most relevant and conceptually rich
- Data extraction charts initially may help in the review but not an essential feature of the CIS approach
- The review may not not be strictly reproducible, auditable. It can be argued that even with primary data in a qualitative study or other evidence synthesizing techniques it is most likely that a different set of researchers may interpret the same data/ set of evidence and come up with the different theoretical model.
- The synthesizing argument “theoretical framework” is a product of interpretive process and“ authorial voice” .Therefore there is a need for constant reflexivity throughout the review process on the part of the authors.

The initial review question

For peer review only - http://bmjopen.bmj.com/site/about/guidelines.xhtml

How do the multilevel actors, institutional structures, processes interact and influence the patient rights implementation in health care facilities? How do they work at what level and in which contexts?

Objectives

The objectives of this review are two fold:

1)To describe the institutional arrangements, strategies, approaches and instruments at various levels for patient rights implementation in health facilities 2)To propose a theoretical framework on the implementation of patient rights in health facilities from a multilevel governance perspective

Methods

Operational definitions of the key terminologies used in the review:

Patient rights- In this review the scope of patient rights covers the patient rights applicable to facility-based preventive, promotive , curative and rehabilitative health care services offered in the primary, secondary and tertiary level health care settings

Patient rights implementation approach refers to the overall strategy and the plan of action taken by the state and/or the various non-state actors such as communities, non-governmental organizations, civil society organizations and professional bodies to respect, protect and fulfill the patient rights.

Patient rights implementation instruments refers to the set of procedures, methods and tools available to implement patient rights. Examples include patient grievance redressal committees, patient rights charter, ombudsman, public hearing, parliamentary hearing,

For peer review only - http://bmjopen.bmj.com/site/about/guidelines.xhtml

strategic litigation by NGO(s), public protests & patient directly filing a petition in the court system.

Patient rights implementation mechanisms refers to the means and the ways through which a particular patient rights enforcement strategy/ instrument is expected to work in a given context

Multilevel governance is about the interaction of the vertical (intergovernmental) and the horizontal (state society dimension) institutions/actors at various levels for the implementation of patient rights in health facilities.

The initial conceptual framework to guide the evidence synthesis

Since the focus of this review is the instutional mechanisms for patient rights implementation, the initial conceptual framework **(see Figure 2)** is developed from the concepts derived from the literature on patients rights [4,18,21-23,28-33] and public policy implementation [60-62]. To explore the institutional dimension of patient rights we embedded the fit, interplay and scale framework on multi level governance in our conceptual scheme[63]. The fit, interplay and the scale themes are in alignment with the broader definition of multilevel governance adopted for this review. The role of language and discourse is critical in the study of multilevel governance[63]. Therefore, we adopted the idea of discourse analysis[64] on patient rights in this initial framework. This starter framework will be further refined using the CIS technique in this systematic scoping review. The framework could be interpreted as a linear one which may not be the case. The elements of the framework might be inter related in a number of ways.

Patient rights implementation approaches

For peer review only - http://bmjopen.bmj.com/site/about/guidelines.xhtml

The implementation approaches to respect,protect and promote patient rights in health facilities could vary across the settings. A number of factors might determine the implementation approaches. To name a few, the political ideology of the state, political framing of the (patient) rights issues, type of health systems and availability of resources for delivering quality health care. The failure of the state's institutional mechanisms to adequately protect the patient rights may result in alternative institutional arrangements steered by the non-state actors such as the professional associations, state independent health care quality accrediting bodies, civil society, media etc.

Fit, interplay and the scale of institutions

The key element of the framework is the institutional dimension of patient rights implementation. By institutions we refer to the thick and the thin definitions of institutions. In the thin sense , institutions refer to the formal explicit set of rules and decision making units i.e rules in paper. On the contrary the thick sense of institutions encapsulate the "social practices" that emerge out of informal understandings between the actors i.e. rules in use. There are three lines of inquiry in studying the institutions 1) fit – the degree to which the spatial, functional and temporal institutional arrangements fit the context. Simply put, it is to see whether the institutional arrangements fit the dynamics of policy problem addressed (in this case- patient rights implementation) 2) interplay- concerns the horizontal interaction between the same level and the vertical interaction across the different levels of the institutions and actors. There could be political or functional interdependencies across the institutions. 3) scale is about the density and the degree of diversity of institutions and actors from local to higher levels. The institutions can range from homogenous to heterogenous , few to many and individual actor influence to policy communities. In general

For peer review only

the scale could be visualized as the shift from simple to complex systems as we move higher up in the levels[63].

Resources requirements

Political, financial, managerial and technical competencies of the actors/ institutions at the national and the subnational levels define the effectiveness of policy instruments used to enforce the patient rights in health facilities*.* *Human resources* entail the availability of adequate and competant actors for patient rights implementation. Public engagement in the policy processes concerning patient rights is key. *Financial resources* refer to the funds that are allocated for facilitating patient rights, policies implementation activities (e.g. recruiting manpower, logistics, infrastructure, equipment, supplies, training, etc.). *Managerial resources* indicate the availability of tools and monitoring mechanisms within the patient rights' enforcement system (e.g. reporting, recording, supervision of the enforcing officials). *Technical resources* denote the existence of technical capacity and assistance for policy implementation e.g. use of information and communication technology. All of these resources will influence the accessibility and availability of the patient rights' policies in the manner comprehensible to the stakeholders, including citizens (and the most vulnerable groups) [60].

Constellation of discourses on patient rights and other contextual factors

Patient rights are within a contentious policy area. Therefore implementation of patient rights should not be seen as only a matter of clear policy design and matching resources for implementation. The policy documents related to patient rights (e.g. Patient rights charter) , implementation strategies and instruments are a product of a certain context and time and hence have been framed in certain discourses. Understanding how the issues on patient

For peer review only - http://bmjopen.bmj.com/site/about/guidelines.xhtml

rights are constituted, represented, given shape and meaning within the policies is cruial as it determines the way the implementation is finally panned out within the multiple tiers of the polity[64].

Strategies and the policies designed to respect, protect and fulfill the patient rights are embedded within a specific social,political,cultural,economic, legal and organizational context. The contextual conditions at the i) micro (facility and the individual patient provider) level e.g. nature of the patient provider relationship, meso (district and subdistrict) level e.g. organizational culture of the implementation bodies and macro level (national and subnational) e.g. economic policies, reforms in health professional education and broader societal and the cultural issues (e.g. social exclusion and discrimination based on gender, class etc will influence the implementation processes. Further, it will be an easier task to implement patient rights, when the players supposed to practice them have somehow ‘internalized’ the notion and the importance of patients rights’[65]. In that respect that there should be room in other policies that have implications for patient rights implementation e.g. adapting the training curricula of health care providers, health systems managers, public health professionals etc.

Multiple potential outcomes

The complex interaction of the actors/institutions, the implementation approaches, instruments and the policy context at various levels and broader societal context will determine the actual implementation practices[60]. Again the patient rights implementation practices could vary based on the nature of the ownership and the mission statement of the health care institution for which it exists. For instance, there could be a private health care

For peer review only - http://bmjopen.bmj.com/site/about/guidelines.xhtml

institution with a public goal or a public health care institution with a profit goal for sustainability reasons [66].

When the patient rights policies are implemented effectively and conducive context conditions are in place , this should result in enhanced accountability and equity in the health system [23,29,30]. Further, at the health facility level, this is expected to lead to improved quality of care[18,21,22] and to promote a culture of respecting and protecting the patient rights[4]. In case of ineffective implementation of patient rights policies/strategies, there will be more discriminatory practices in the health facilities, contributing to the denial of patient rights, widening health inequities and poor accountability in the health system[24,29-33]. Also, it is possible that in settings where there is poor response of the state to the patient rights violations, people might feel frustrated, lose trust in the health system and resort to violence against the health care providers[23].

Eligibility criteria, data sources & search strategy

There are three main data sources for this review 1) select databases 2) grey literature 3) reference tracking and citation tracking. To get a comprehensive understanding on patient rights implementation strategies and instruments from a multidisciplinary perspective, three databases PubMed (health literature), Web of Science (social sciences), Lexis Nexis (Law) will be searched in a systematic manner for all types of evidence (both empirical and non-empirical) broadly on key concepts such as patient rights, patient rights instruments and person centered care. The related terms reflecting the key concepts will be used as search terms in the databases **(see Table 1).** The search terms will be pilot tested in the PubMed database. Boolean operators will be used to develop various combinations of search terms feasible for usage in the PubMed database. Followed by PubMed, a more

For peer review only - http://bmjopen.bmj.com/site/about/guidelines.xhtml

For peer review only

detailed search will be undertaken in the Web of Sciences and Lexis Nexis databases. We have provided our detailed search strategy for the PubMed database in the **supplementary file 2**. Further, targeted searches will be performed in grey literature 1) Google 2) Websites of the institutions (public and private) that are relevant to the area of patient rights, social justice, human rights and accountability in health systems (e.g. Open Society Foundations, People's Health Movement , health care quality accreditation agencies, other civil society institutions(national and international), Patient rights organizations (national and international) e.g. Coalition patient rights, National Patient Advocate Organization etc. Also, the reference list of the specific papers(reference tracking) and the papers that cite the key papers (citation tracking) will be hand searched to identify any additional studies that are conceptually relevant and have the potential to contribute to the emerging framework.

Table 1:Key search domains and the related terms to be used in different permutations and combinations in the selected databases

**Key search domains**	**Related terms**
Patient rights	Patient bill of rights, patient charter, ,quality of health care, social(community) accountability in health,Patient accountability, patient safety, discrimination, health equity, equality, right to health, patient rights and ethics
Patient rights instruments	Patient rights legislation, patient rights charter, patient bill of rights, health ombudsman, right to information act,

For peer review only - http://bmjopen.bmj.com/site/about/guidelines.xhtml

	health councils, patient welfare committees, patient grievance redressal systems/ committees, patient complaints system, patient advocates, parliamentary hearings, public hearing, public protests, strategic litigation, consumer forums, professional associations, social audit, audit bodies, patient suggestion box, court system for patient rights, human rights commissions, health care quality councils
Person centered care	Patient centered care, people centerd care, patient autonomy, patient engagement, patient participation, patient empowerment

Note: The search terms mentioned in the table are only indicative and not exhaustive. The list will be further enriched after the initial search for the studies in the PubMed database

Period of search

The aim of the literature search is to maximise the possibility of identifying the conceptually rich articles to enable theory building. Hence in the database searches, no specific start date will be mentioned. However, the end date for the database search will be 30th April 2020.

Publication type

As indicated in the state of the art literature on patient rights, it is clear that the available scholarship is mostly from the European contexts.It is expected that there will be limited empirical work on the patient rights in the LMICs. In order to increase the transferability of

For peer review only - http://bmjopen.bmj.com/site/about/guidelines.xhtml

the theoretical framework to the LMIC settings it was decided that all types of evidence (both empirical and non-empirical) in English from both LMIC and HIC will be included in this review.

Time period scheduled for the review

The review will be conducted between 15/04/2020 and 15/08/2020.

Article selection process

1. Systematic search for the articles on patient rights implementation in the selected three data bases
2. Identifying the relevant articles in grey literature and through citation and reference tracking
3. Title and abstract review of the articles to identify the relevant articles that are concordant with the initial review question
4. Creating a sampling frame of all articles
5. Purposive sampling of the articles from the sampling frame that point towards relevancy and conceptual soundness
6. Applying the quality appraisal criteria for the individual papers. Perform sensitivity analysis to strike a balance between methodological rigour and conceptual breadth and depth of the study. To give more weightage to the articles that score high on quality
7. Initial data extraction on the study characteristics
8. Concept mapping in the retrieved literature and identification of the conceptual gaps
9. Repeating the search in the selected databases,grey literature, citation tacking and reference tracking to fill the conceptual gaps until a theoretical saturation is reached

For peer review only - http://bmjopen.bmj.com/site/about/guidelines.xhtml

and refining the synthesizing argument i.e.“theoretical framework”on the patient rights implementation

The article selection process is schematically shown in **Figure 3.**

Quality appraisal criteria

Since the review uses articles emerging from atleast three diverse disciplines (public health, social sciences and law), finding a common quality assessment framework is challenging. However, it is expected that majority of the empirical work will be of qualitative in nature. Hence, the quality of the studies will be evaluated using the Hannes criteria (2011) [67] for assessing qualitative research **(see supplementary file 3).** The main parameters for evaluation would be 1) credibility 2)transferability 3) dependability 4) confirmability. The quality of the study will be determined using a three point likert scale(strong moderate and weak). For quantitative studies, quality appraisal tool developed by Effective Public Health Practice Project[68] will be used **(see supplementary file 4).**

Data extraction

The usefulness of the formal data extraction sheet for a complex review topic that uses critical interpretive synthesis technique is questioned [56]. Due to the huge set of variables involved, a formal data extracton sheet might appear messy and practically difficult to analyse. So we devised a simple data extraction proforma which include details such as the author,author affiliations, year of publication, article type, study design, funding for the study, setting and country **(see supplementary file 5)**. The broad themes, categories and the subcategories as identified in the conceptual framework and also emerging from the data would be captured and organized using the NVIVO version 12 software. The first

For peer review only - http://bmjopen.bmj.com/site/about/guidelines.xhtml

investigator will extract the data and this will be independently checked atleast by two other senior researchers of the review team. Further the four authors MP, SVB, NE and PNS will deliberate on the data collection and the data analysis at regular intervals.

Patient and Public involvement

Patient and public involvement is not applicable in this review as the research uses already published literature.

Synthesis of results

The CIS approach used in the evidence synthesis is inductive and iterative in nature. The retrieved items/papers will be critically analysed for patterns and themes that explain the phenomena of patient rights implementation in the literature. By constant comparison of the theoretical constructs with the data in the papers, the relationships between the theoretical constructs will be identified.The conceptual gaps identified will be further filled with subsequent search and analysis of the literature.The aim is to be critical while synthesizing the evidence by questioning 1) the ways in which the patient rights has been problematized or represented to be in the literature 2) the underlying assumptions behind the policy options or the implementation strategies for patient rights.The synthesis of the evidence retrieved in this review would thus result in a unifying framework (i.e.the synthesizing argument) on the implementation of patient rights from a multilevel governance perspective that is grounded in the data.

Limitations

Rights and responsibilities goes together. The review focusses on the rights of the patient and we do not venture into the obligations of the patient in the health care processes.

For peer review only - http://bmjopen.bmj.com/site/about/guidelines.xhtml

Patient rights pertaining to health research context and specific conditions such as mental health disorders, disabilities, genetic screening, euthanasia and advance medical directives merit specific consideration and hence these are out of the scope of this review. In this review we deal with the institutional arrangements for patient rights protection in the health care facilities that are formally recognized and considered legitimate by the state. We exclude the health care facilities run by the informal health care providers. The international institutional mechanisms such as the Office of the United Nations High Commissioner on Human Rights (OHCHR), the Human Rights Council etc are not touched upon in this inquiry mainly because of the time constraints.

Registration

This protocol is registered in PROSPERO (https://www.crd.york.ac.uk/prospero/display_record.php?RecordID=176939). The registration ID is CRD2020176939.

Ethics and dissemination

Ethical approval will not be required because this study will retrieve and synthesise data from already published literature.The findings of this review will be published in a peer reviewed journal.

References

1. United Nations. Universal Declaration of Human Rights. General Assembly resolution 217 A. Newyork: United Nations;1948. Available from: https://www.un.org/en/universal-declaration-human-rights/. Accessed 13 Jan 2020.

For peer review only - http://bmjopen.bmj.com/site/about/guidelines.xhtml

2. Imani O N. "Critical Impairments to Globalizing the Western Human Rights Discourse."Societies Without Borders.2008; 3 (2): 270-284.Available at:https://scholarlycommons.law.case.edu/swb/vol3/iss2/. Accessed 13 Jan 2020.
3. Panikkar R. Is the notion of Human rights a western concept? Diogenes 120; 1982.Avaliable at:http://www.derechoshumanos.unlp.edu.ar/assets/files/documentos/is-the-notion-of-human-rights-a-western-concept.pdf.Accessed 15 Jan 2020.
4. Peled-Raz M. Human rights in patient care and public health-a common ground. Public Health Rev. 2017; 38:29.
5. Cohen J & Ezer T. Human rights in patient care: a theoretical and practical framework. Health Hum Rights. 2013 Dec 12;15(2):7-19.
6. World Health organization. Patient Rights. Geneva: World Health Organization;2019. Available at: https://www.who.int/genomics/public/patientrights/en/ . Accessed 20 Jan 2020.
7. The free dictionary by Farlex.Patient rights. Pennyslvania:Farlex, Inc;2003-2019.Available from: https://medical-dictionary.thefreedictionary.com/patient+rights.Accessed 02 Jan 2020.
8. Townand D, Clemens T, Shaw D, Brand H, Nys H & Palm W.Patient's rights in the European Union, A mapping exercise. Luxembourg: European Union; 2016.
9. Méndez J. Report of the Special Rapporteur on torture and other cruel, inhuman or degrading treatment or punishment. New York: United Nations General Assembly;2013.
10. Reader WT & Gillespie A. Patient neglect in health care institutions: A systematic review and conceptual model. BMC Health Ser Res.2013;13:156.

For peer review only - http://bmjopen.bmj.com/site/about/guidelines.xhtml

11. Bell SK, Roche SD, Mueller A, et al Speaking up about care concerns in the ICU: patient and family experiences, attitudes and perceived barriers. BMJ Qual Saf. 2018; 27:928-936.

12. Tong WT, Lee YK, Ng CJ, Lee PY. Factors influencing implementation of a patient decision aid in a developing country: an exploratory study. Implement Sci. 2017 ;12(1):40.

13. Cubaka VK, Schriver M, Kayitare JB, Cotton P, Maindal HT, Nyirazinyoye L & Kallestrup P. 'He should feel your pain': Patient insights on patient-provider communication in Rwanda. Afr J Prim Health Care Fam Med. 2018:10(1).

14. Fochsen, G, Deshpande, K, & Thorson, A. (2006). Power Imbalance and Consumerism in the Doctor-Patient Relationship: Health Care Providers' Experiences of Patient Encounters in a Rural District in India. Qualitative Health Research, 16(9), 1236–51.

15. Henderson S. Power imbalance between nurses and patients: a potential inhibitor of partnership in care. J Clin Nurs. 2003 Jul;12(4):501-8.

16. Mead N, Bower P. Patient-centredness: a conceptual framework and review of the empirical literature. Soc Sci Med. 2000 Oct;51(7):1087-110.

17. Virona Gabriella M & Tarasenka N. Patient safety and rights: developing tools to support consumer health literacy. Regional Office for Europe. World Health Organization;2010.

18. Institute of Medicine. Crossing the Quality Chasm: A New Health System for the 21st Century. Washington DC: National Academy Press; 2001.

19. World Health Organization. Quality of care:A process for making strategic choices in health systems.Geneva:World Health Organization;2006.

For peer review only

20. Groene O. Patient centeredness and quality improvement efforts in hospitals: rationale, measurement, implementation. Int J Qual Health Care. 2011; 23(5): 531–37.

21. World Health Organization & UNICEF. Declaration of Asthana. Global conference on primary Health Care;2018. Available from: https://www.who.int/primary-health/conference-phc/declaration. Accessed Feb 20 2020.

22. Van Belle S, Boydell V, George AS, Brinkerhoff DW, Khosla R. Broadening understanding of accountability ecosystems in sexual and reproductive health and rights: A systematic review. PLoS One. 2018:13(5);e0196788.

23. Mirzoev T &Kane S.Key strategies to improve systems for managing patient complaints within health facilities- What can we learn from the existing literature?. Glob Health Action.2018;11:1458938.

24. Parliamentary and Health Service Ombudsman Impact study presentation of findings. London (United Kingdom): The Ombudsman;2010. Available at: www.ombudsman.org.uk/__data/assets/pdf_file/0013/4144/Impact-Study.pdf. Accessed Jan 02 2020.

25. Paterson R.The patients complaint system in Newzeland.Health Aff.2002;21(3):70-9.

26. Annual report 2009–2010. The Ombudsman: Quebec; 2010. Available at: www.protecteurducitoyen.qc.ca/en/cases-and-documentation/index.html. Accessed Feb 02 2020.

27. Francis R. Independent Inquiry into care provided by Mid Staffordshire NHS Foundation Trust January 2005 – March 2009. London: The Stationery Office;2010.

For peer review only

For peer review only - http://bmjopen.bmj.com/site/about/guidelines.xhtml

28. European Union Agency for fundamental rights. Inequalities and multiple discrimination in access to and quality health care.Luxembourg:European Union Agency for Fundamental Rights; 2013.

29. Glover ML, Sim M & Winters K. Perceived discrimination and reported trust and satisfaction with providers in African Americans. The Jackson heart study. Ethnicity & Disease.2017;27(3): 209-15.

30. Goerge S. Caste and Care: Is Indian Healthcare delivery system favourable for Dalits?Bangalore:The Institute of Social and Economic Change;2015.

31. Irudayam, A, J P Mangubhai & J G Lee. Dalit Women Speak Out: Violence against Dalit Women in India, Volume I and II. New Delhi: National Council for Dalit Human Rights;2006.

32. Open Society Foundations. Reports on the Patient Rights Violations in Krygystan. Newyork: Open Society Foundations;2008.

33. Bowser D & Hill K.Exploring evidence for disrespect and abuse in facility based childbirth.Report of a landscape analysis.Washington DC:USAID;2010.

34. Hammonds R, Hanefeld J & Ooms G. Accountablity as a driver of heath equity.Denmark: WHO Regional Office for Europe;2019.

35. Open Society Foundations. International/European framework for protection of human rights in patient care. Public Health Fact Sheet.Newyork: Open Society Foundations;2013.Available at: https://www.opensocietyfoundations.org/publications/international-european-framework-protection-human-rights-patient-care. Accessed Feb 19 2020.

For peer review only

For peer review only - http://bmjopen.bmj.com/site/about/guidelines.xhtml

36. National Human Rights Commission of India. Draft of patient rights charter. NewDelhi: Ministry of Health and Family Welfare;2018.Available at: https://mohfw.gov.in/newshighlights/draft-patient-charter-prepared-national-human-rights-commission.Accessed Feb 08 2020.

37. Department of Quality Assurance. Ministry of Health. Republic of Uganda. Patient's Charter:Uganda; Republic of Uganda;2009.Available at: http://cphl.go.ug/sites/default/files/library resources/PATIENTS%27%20CHARTER.pdf. Accessed Jan 22 2020.

38. Boudioni M, McLaren S & Lister G. A critical analysis of national policies, systems, and structures of patient empowerment in England and Greece. Patient Prefer Adherence. 2017; 11:1657–69.

39. Ontario Ministry of Health Care. Patients first :Action Plan for health care. Ontario: Ministry of long term care;2008. Available at: http://www.health.gov.on.ca/en/ms/ecfa/healthy_change/faqs_patientombudsman.aspx. Accessed Jan 03 2020

40. Shukla A, More A & Marathe S. Making private health care accountable:Mobilizing civil society and ethical doctors in India.Institute of Development Studies Bulletin.2018;49(2): 129-46.

41. Open Society Foundations.Strategic litigation impact:Insights from global experience.Newyork:Open Society Foundation;2018.

42. Unnikrishnan B, Trivedi D, Kanchan T, Rekha T, Mithra P, Kumar N, Kulkarni V, Holla R &Talish M. Patients' Awareness About Their Rights: A Study from Coastal South India. Sci Eng Ethics. 2017 Feb;23(1):203-14.

For peer review only - http://bmjopen.bmj.com/site/about/guidelines.xhtml

43. Agrawal U, D'Souza BC & Seetharam AM. Awareness of Patients' Rights among patients of a Tertiary Care Teaching Hospital- A Cross-sectional Study. J Clin Diagn Res. 2017 ;11(9): IC01-IC06.

44. Kagoya HR, Kibuule D, Mitonga-Kabwebwe H, Ekirapa-Kiracho E, Ssempebwa JC. Awareness of, responsiveness to and practice of patients' rights at Uganda's national referral hospital. Afr J Prim Health Care Fam Med. 2013;5(1):491. doi:10.4102/phcfm.v5i1.491

45. Abedi G, Shojaee J, Moosazadeh M, et al. Awareness and Observance of Patient Rights from the Perspective of Iranian Patients: A Systematic Review and Meta-Analysis. Iran J Med Sci. 2017;42(3):227–34.

46. Brinkerhoff DW, Bossert TJ. Health Governance: Concepts, Experience and Programming Options. Health Systems 2020 Policy Brief. Health Systems 2020.US:USAID;2008.

47. Institute of Governance. Definition of Governance. Canada: Institute of Governance; 2020.Available at https://iog.ca/what-is-governance/. Accessed Jul 18 2020.

48. Abimbola S, Negin J, Stephen J & Martiniuk A. Towards people centered health systems: multilevel framework for analyzing primary health care governance in low & middle income countries. Health Policy Plan. 2014; 29:ii29-ii39.

49. Marks G & Hooghes L. Themes and issues in multi-level governance. in Bache, I & Flinders, M (Editors). Multi-level governance. New York: Oxford University Press; 2004.

50. Gregoria DM, Fatorelli L, Paavola J, Locatelli B, Pramova E, Nurrochmat RD, May HP….& Kusumadewi DS. Multilevel governance and power in climate change policy networks. Global Environmental Change.2019;54:64-77.

For peer review only

51. Touati N, Maillet L, Paquette AM ,Denis DJ & Rodriguez C. Understanding Multilevel Governance Processes through Complexity Theory: An Empirical Case Study of the Quebec Health-Care System. International Journal of Public Administration. 42:3;205-17.

52. Ongaro E, Massey A, Holzer M & Wayenberg E.Governance and intergovernmental relations in the European Union and United States : theoretical perspectives. UK: Edward Elgar Publishing Limited;2010.

53. Daniell KA & Kay A. Multi-level governance: Conceptual challenges and case studies from Australia. Canberra:The Australian National University Press Canberra;2017.

54. Munn Z, Peters DJM, Stern C, Tufanaru C, McArthur A & Aromataris E. Systematic review or scoping review? Guidance for authors when choosing between a systematic or scoping review approach. BMC Med Res Methodol.2018;18:143.

55. Shameer L, Moher M, Clarke M, Ghersi D, Liberati A, Petticrew M…..& PRISMA-P group. Preferred reporting items for systematic review and meta-analysis protocols (PRISMA-P)2015:elaboration and explanation. BMJ.2015;349:g7647.

56. Dixon-Woods M, Cavers D, Agarwal S, Annandale E,Arthur A, Harvey J…..& Sutton JA. Conducting a critical interpretive synthesis of the literature on access to health care by vulnerable groups. BMC Med Res Methodol.2006;6:35.

57. Schaaf M & Topp MS. A critical interpretive synthesis of informal payments in maternal health care.Health Policy Plan.2019;34:216-29.

58. Wilson GM, Ellen EM, Lavis NJ, Grimsahw MJ, Moa A K,Shemer J et al. Processes, contexts and rationale for disinvestment: a protocol for a critical interpretive synthesis.Syst Rev.2014;3:143.

For peer review only

For peer review only - http://bmjopen.bmj.com/site/about/guidelines.xhtml

59. Mold A. Patient's rights & the National Health Service in Britain1960s-1098s. Am J Public Health.2012;102(11): 2030-37.

60. Grindle SM &Thomas WJ. Public choices and policy change. The political economy of reform in developing countries. Baltimore: John Hopkins University Press; 1991.

61. Sabatier P, Mazmanian D. The implementation of regulatory policy: A framework of analysis. Davis: Institute of Governmental Affairs; 1979.

62. Hill M, Hupe P. In: Aguilera , editor. Implementing Public Policy. 3rd ed. London: Sage Publications; 2014.

63. Young OR. The institutional dimension of environmental change: fit, interplay, and scale. Cambridge, Massachusetts: MIT Press;2002.

64. Bacchi. C. Problematization in health policy:Questioning how "problems" are constituted in policies.SAGE Open.2016;1-16.

65. Thaler HR & Sunstein RC.Nudge Improving decisions about health, wealth & Happiness.Yale University Press:New Haven& London;2008.

66. Giusti D, Criel B & Bethune DX.Viewpoint:public versus private health care debate beyond the slogans. Health Policy Plan.1997;12(3):193-98.

67. Hannes K. Chapter 4: Critical appraisal of qualitative research. In: Noyes J, Booth A, Hannes K, Harris J, Lewin S, Lockwood C(editors), Supplementary guidance for inclusion of qualitative research in Cochare systematic reviews of interventions.Version 1(updated August 2011). Cochare Colloboration Qualitataive Methods Group; 2011.

68. Ciliska D, Miccouci S, Dobbins M & Thoas HP.Effective Public Health Practice Project. Quality assessment tool for quantitative studies. Hamilton, on: Effective Public Health Practice Project;1998. Available at: https://merst.ca/ephpp/. Acessed Feb 03 2020.

Figure legends

For peer review only - http://bmjopen.bmj.com/site/about/guidelines.xhtml

Figure 1:The three main and the two cross cutting categories of patient rights.

Source:Townend et al, 2016.

Figure 2:The initial conceptual framework on the implementation of patient rights in health facilities from a multilevel governance perspective

Figure 3:Article selection and analysis algorithm

Supplementary files

Supplementary file 1

PRISMA-P checklist

Supplementary file 2

Detailed search strategy in PubMed database

Supplementary file 3

Quality appraisal criteria for qualitative studies

Suppplementary file 4

Quality appraisal criteria for quantitative studies

Supplementary file 5

Data extraction sheet

Authors contribution

MP and SVB led the conceptualization and the design of the protocol. MP drafted the manuscript. All authors (MP,SVB,BC,NE,AK,PBN and PNS) provided inputs , have read and approved this manuscript.

For peer review only - http://bmjopen.bmj.com/site/about/guidelines.xhtml

Funding statement

This work is supported by the PhD fellowship offered to Meena Putturaj by the Institute of Tropical Medicine, Antwerp, Directorate of Development Cooperation, Belgium.The time and the contributions of PNS for this project is supported by the Wellcome Trust/DBT India Alliance Intermediate Clinical and Public Health Research Fellowship awarded to him [IA/CPHI/16/1/502648].

Competing interests

The authors declare that they have no competing interests. The funders do not have any role in the design and the outcomes of this research project.

For peer review only

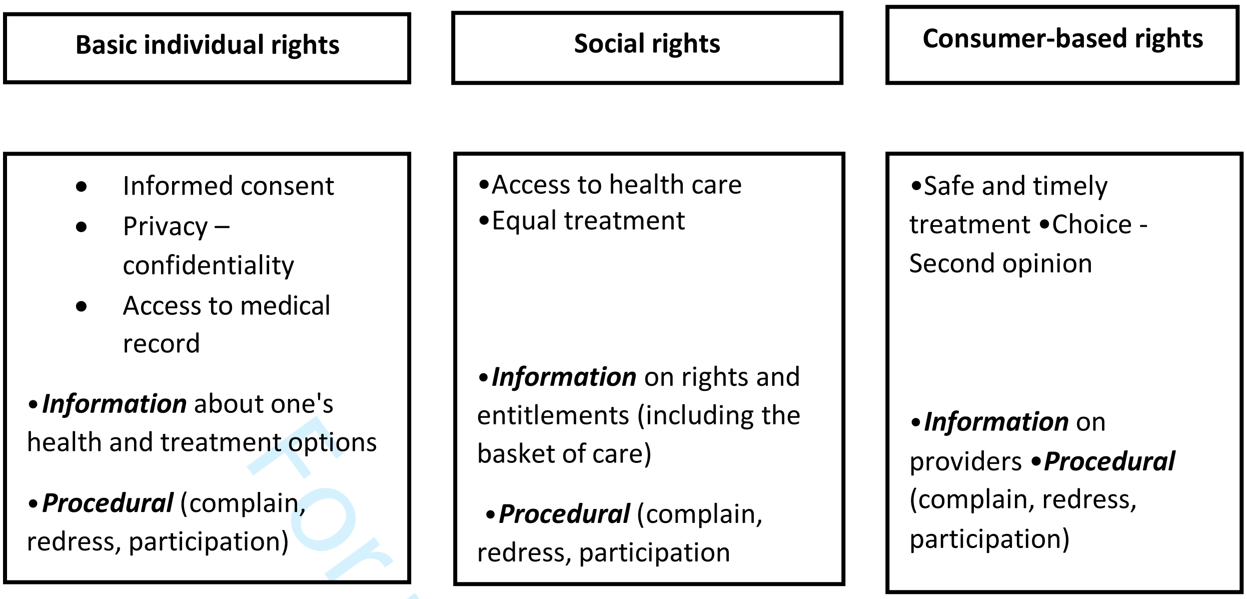

Fig 1:The three main and the two cross cutting categories of patient rights

Source: Townend et al, 2016

For peer review only

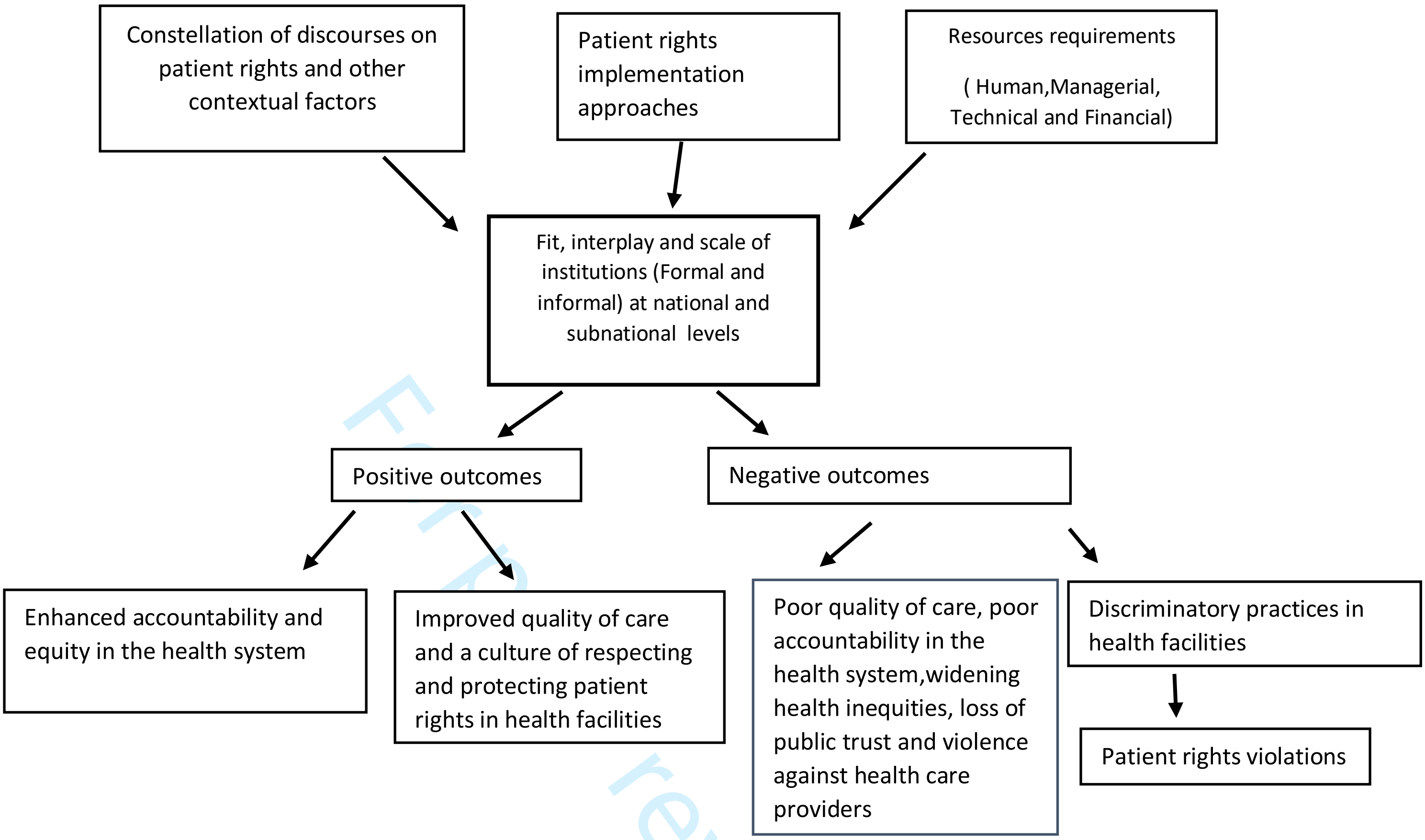

Fig 2:The initial conceptual framework on the implementation of patient rights in health facilities from a multi-level governance perspective

For peer review only

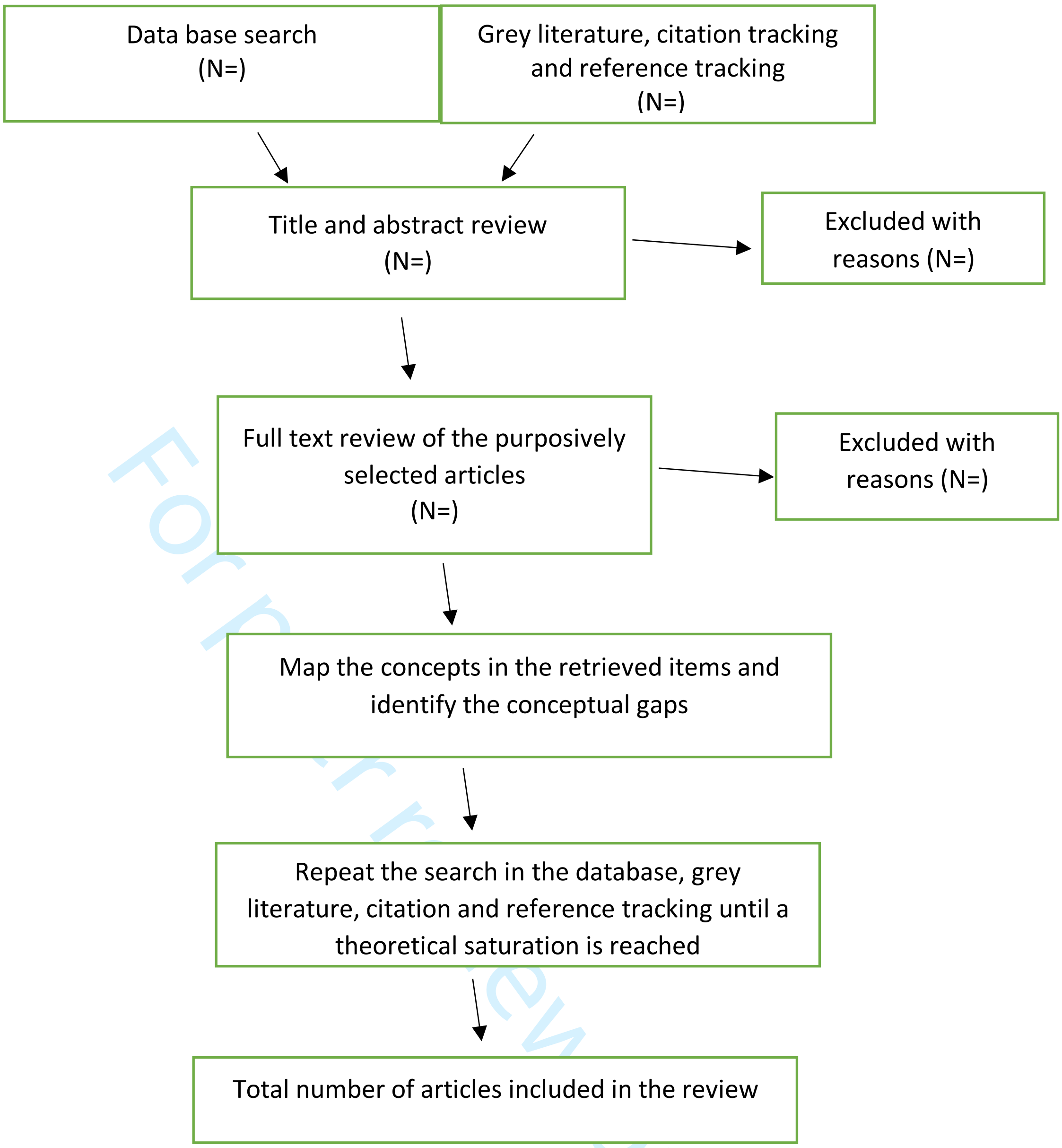

Fig3 : Article selection and analysis algorithm

For peer review only

For peer review only - http://bmjopen.bmj.com/site/about/guidelines.xhtml

PRISMA-P (Preferred Reporting Items for Systematic review and Meta-Analysis Protocols) 2015 checklist: recommended items to address in a systematic review protocol*

Section and topic	Item No	Checklist item	Reported on page
**ADMINISTRATIVE INFORMATION**			
Title:			
Identification	1a	Identify the report as a protocol of a systematic review	2
Update	1b	If the protocol is for an update of a previous systematic review, identify as such	Not applicable
Registration	2	If registered, provide the name of the registry (such as PROSPERO) and registration number	3&24
Authors:			
Contact	3a	Provide name, institutional affiliation, e-mail address of all protocol authors; provide physical mailing address of corresponding author	1
Contributions	3b	Describe contributions of protocol authors and identify the guarantor of the review	33
Amendments	4	If the protocol represents an amendment of a previously completed or published protocol, identify as such and list changes; otherwise, state plan for documenting important protocol amendments	Not applicable
Support:			
Sources	5a	Indicate sources of financial or other support for the review	34
Sponsor	5b	Provide name for the review funder and/or sponsor	34
Role of sponsor or funder	5c	Describe roles of funder(s), sponsor(s), and/or institution(s), if any, in developing the protocol	34
**INTRODUCTION**			
Rationale	6	Describe the rationale for the review in the context of what is already known	4-12
Objectives	7	Provide an explicit statement of the question(s) the review will address with reference to participants, interventions, comparators, and outcomes (PICO)	13
**METHODS**			
Eligibility criteria	8	Specify the study characteristics (such as PICO, study design, setting, time frame) and report characteristics (such as years considered, language, publication status) to be used as criteria for eligibility for the review	18-21
Information sources	9	Describe all intended information sources (such as electronic databases, contact with study authors, trial registers or other grey literature sources) with planned dates of coverage	18-21

For peer review only

Search strategy	10	Present draft of search strategy to be used for at least one electronic database, including planned limits, such that it could be repeated	18-21, Supplementary file 1
Study records:			
Data management	11a	Describe the mechanism(s) that will be used to manage records and data throughout the review	22
Selection process	11b	State the process that will be used for selecting studies (such as two independent reviewers) through each phase of the review (that is, screening, eligibility and inclusion in meta-analysis)	21,23
Data collection process	11c	Describe planned method of extracting data from reports (such as piloting forms, done independently, in duplicate), any processes for obtaining and confirming data from investigators	22,23
Data items	12	List and define all variables for which data will be sought (such as PICO items, funding sources), any pre-planned data assumptions and simplifications	13,14-18
Outcomes and prioritization	13	List and define all outcomes for which data will be sought, including prioritization of main and additional outcomes, with rationale	13,14-18
Risk of bias in individual studies	14	Describe anticipated methods for assessing risk of bias of individual studies, including whether this will be done at the outcome or study level, or both; state how this information will be used in data synthesis	22
Data synthesis	15a	Describe criteria under which study data will be quantitatively synthesised	Not applicable
	15b	If data are appropriate for quantitative synthesis, describe planned summary measures, methods of handling data and methods of combining data from studies, including any planned exploration of consistency (such as I^2, Kendall's τ)	Not applicable
	15c	Describe any proposed additional analyses (such as sensitivity or subgroup analyses, meta-regression)	Not applicable
	15d	If quantitative synthesis is not appropriate, describe the type of summary planned	23
Meta-bias(es)	16	Specify any planned assessment of meta-bias(es) (such as publication bias across studies, selective reporting within studies)	Not applicable
Confidence in cumulative evidence	17	Describe how the strength of the body of evidence will be assessed (such as GRADE)	Not applicable

*** It is strongly recommended that this checklist be read in conjunction with the PRISMA-P Explanation and Elaboration (cite when available) for important clarification on the items. Amendments to a review protocol should be tracked and dated.** **The copyright for PRISMA-P (including checklist) is held by the PRISMA-P Group and is distributed under a Creative Commons Attribution Licence 4.0.**

From: Shamseer L, Moher D, Clarke M, Ghersi D, Liberati A, Petticrew M, Shekelle P, Stewart L, PRISMA-P Group. Preferred reporting items for systematic review and meta-analysis protocols (PRISMA-P) 2015: elaboration and explanation. BMJ. 2015 Jan 2;349(jan02 1):g7647.

For peer review only

Supplementary file 2

Detailed search strategy in the PubMed database

Table 1: Key concepts and related free text terms for search in the database

	**Key concept 1**	**Key concept 2**	**Key concept 3**
	**Patient rights**	**Patient rights instruments**	**Person centered care**
**Related free text terms**	Patient bill of rights, patient rights charter, quality of health care, social(community) accountability in health, Patient accountability, patient safety, discrimination, health equity, equality, right to health, patient rights and ethics	Patient rights legislation, patient bill of rights, health ombudsman, Right to information act, health councils, patient welfare committees, patient grievance redressal systems/ committees, patient complaints system, patient advocates, parliamentary hearings, public hearing, public protests, strategic litigation, consumer forums, professional associations, social audit, audit bodies, patient suggestion box, court system for patient rights, human rights commissions, health care quality councils	Patient centered care, people centered care, patient autonomy, patient engagement, Patient participation, Patient empowerment

The free text terms for each of the key concepts mentioned in Table 1 were pilot tested in the PubMed database. Each free text terms were searched separately to see whether the search results are relevant. The results of the initial search in the PubMed database are shown in Table 2. Since the initial search yielded unmanageable number of records, the search terms were made more specific. Also, use of Boolean operators such as "AND" and application of "free text filter" yielded relevant and manageable number of records. Since the governance aspect (not a core medical topic) of the patient rights implementation is the focus in the review, it was expected that the key concepts of the review may not be well indexed in the PubMed database. Hence, the search field included "All fields". The refined search strategy is shown in Table 3.

For peer review only

Table 2: Results of the pilot testing of the free text terms in the PubMed database

Free text terms	Search results
Patient* bill of rights	529
Community accountability	8128
Patient safety	157872
Health equity	24519
Right to health	66755
Equality	367726
Patient rights and ethics	33598
Quality of health care	7025620
Social accountability	36577
Patient rights legislation	31773
Health ombudsman	321
Right to information act	1952
Health councils	117769
Patient welfare committees	1149
Patient grievance redressal systems	1
Patient complaint system	7540
Patient advocates	30901
Parliamentary hearings	11
Strategic litigation	1501
Consumer forums	1025
Professional associations	74651
Social audit	3558
Audit bodies	433
Patient suggestion box	13669
Court system for patient rights	301
Human rights commission	2252
Health care quality councils	97728
Patient centered care	33517
People centered care	14100
Patient autonomy	21190
Patient engagement	66813
Patient participation	55600
Patient empowerment	58745

For peer review only

Table 3: Refined search strategy in the PubMed database

Data source	Search terms	Search fields	Filters	Search results
PubMed database	Patient bill of rights	All fields	Full text	253
	Patient bill of rights AND implementation	All fields	Full text	23
	Patient charter of rights	All fields	Full text	147
	Patient charter of rights AND implementation	All fields	Full text	22
	Health ombudsman	All fields	Full text	186
	Patient grievance redressal	All fields	Full text	3
	Patient complaints AND Patient rights	All fields	Full text	270
	complaints procedures AND patient rights	All fields	Full text	173
	Rights AND strategic litigation	All fields	Full text	951
	Patient rights AND Consumer forums	All fields	Full text	14
	Consumer Health Forums AND patient rights	All fields	Full text	12
	Quality Health care AND patient bill of rights	All fields	Full text	80

Similar steps will be followed in the Web of Science and Lexis Nexis Database as well. The filters, search fields and the search terms will be chosen based on what is feasible in those databases.

For peer review only

Quality appraisal criteria for the qualitative studies

Criteria	Study 1	Study 2	Study 3	Study …
Credibility				
Transferability				
Dependability				
Confirmability				
Overall quality	H/L/Judge			
Comments				

Note:

- To give more weight to studies that score high on quality
- Sensitivity analyses could be done to strike a balance between the methodological rigor and conceptual breadth and depth

For peer review only

QUALITY ASSESSMENT TOOL FOR QUANTITATIVE STUDIES

EPHPP
Effective Public Health Practice Project

COMPONENT RATINGS

A) SELECTION BIAS

(Q1) Are the individuals selected to participate in the study likely to be representative of the target population?
1 Very likely
2 Somewhat likely
3 Not likely
4 Can't tell

(Q2) What percentage of selected individuals agreed to participate?
1 80 - 100% agreement
2 60 – 79% agreement
3 less than 60% agreement
4 Not applicable
5 Can't tell

RATE THIS SECTION	STRONG	MODERATE	WEAK
See dictionary	1	2	3

B) STUDY DESIGN

Indicate the study design
1 Randomized controlled trial
2 Controlled clinical trial
3 Cohort analytic (two group pre + post)
4 Case-control
5 Cohort (one group pre + post (before and after))
6 Interrupted time series
7 Other specify ____________________
8 Can't tell

Was the study described as randomized? If NO, go to Component C.
No Yes

If Yes, was the method of randomization described? (See dictionary)
No Yes

If Yes, was the method appropriate? (See dictionary)
No Yes

RATE THIS SECTION	STRONG	MODERATE	WEAK
**See dictionary**	1	2	3

For peer review only - http://bmjopen.bmj.com/site/about/guidelines.xhtml

C) CONFOUNDERS

(Q1) Were there important differences between groups prior to the intervention?

1 Yes
2 No
3 Can't tell

The following are examples of confounders:

1 Race
2 Sex
3 Marital status/family
4 Age
5 SES (income or class)
6 Education
7 Health status
8 Pre-intervention score on outcome measure

(Q2) If yes, indicate the percentage of relevant confounders that were controlled (either in the design (e.g. stratification, matching) or analysis)?

1 80 – 100% (most)
2 60 – 79% (some)
3 Less than 60% (few or none)
4 Can't Tell

RATE THIS SECTION	STRONG	MODERATE	WEAK
**See dictionary**	1	2	3

D) BLINDING

(Q1) Was (were) the outcome assessor(s) aware of the intervention or exposure status of participants?

1 Yes
2 No
3 Can't tell

(Q2) Were the study participants aware of the research question?

1 Yes
2 No
3 Can't tell

RATE THIS SECTION	STRONG	MODERATE	WEAK
**See dictionary**	1	2	3

E) DATA COLLECTION METHODS

(Q1) Were data collection tools shown to be valid?

1 Yes
2 No
3 Can't tell

(Q2) Were data collection tools shown to be reliable?

1 Yes
2 No
3 Can't tell

RATE THIS SECTION	STRONG	MODERATE	WEAK
**See dictionary**	1	2	3

For peer review only - http://bmjopen.bmj.com/site/about/guidelines.xhtml

F) WITHDRAWALS AND DROP-OUTS

(Q1) Were withdrawals and drop-outs reported in terms of numbers and/or reasons per group?
1 Yes
2 No
3 Can't tell
4 Not Applicable (i.e. one time surveys or interviews)

(Q2) Indicate the percentage of participants completing the study. (If the percentage differs by groups, record the lowest).
1 80 -100%
2 60 - 79%
3 less than 60%
4 Can't tell
5 Not Applicable (i.e. Retrospective case-control)

RATE THIS SECTION	STRONG	MODERATE	WEAK	
See dictionary	1	2	3	Not Applicable

G) INTERVENTION INTEGRITY

(Q1) What percentage of participants received the allocated intervention or exposure of interest?
1 80 -100%
2 60 - 79%
3 less than 60%
4 Can't tell

(Q2) Was the consistency of the intervention measured?
1 Yes
2 No
3 Can't tell

(Q3) Is it likely that subjects received an unintended intervention (contamination or co-intervention) that may influence the results?
4 Yes
5 No
6 Can't tell

H) ANALYSES

(Q1) Indicate the unit of allocation (circle one)
community organization/institution practice/office individual

(Q2) Indicate the unit of analysis (circle one)
community organization/institution practice/office individual

(Q3) Are the statistical methods appropriate for the study design?
1 Yes
2 No
3 Can't tell

(Q4) Is the analysis performed by intervention allocation status (i.e. intention to treat) rather than the actual intervention received?
1 Yes
2 No
3 Can't tell

For peer review only - http://bmjopen.bmj.com/site/about/guidelines.xhtml

GLOBAL RATING

COMPONENT RATINGS

Please transcribe the information from the gray boxes on pages 1-4 onto this page. See dictionary on how to rate this section.

**A**	**SELECTION BIAS**	**STRONG**	**MODERATE**	**WEAK**	
		1	2	3	
**B**	**STUDY DESIGN**	**STRONG**	**MODERATE**	**WEAK**	
		1	2	3	
**C**	**CONFOUNDERS**	**STRONG**	**MODERATE**	**WEAK**	
		1	2	3	
**D**	**BLINDING**	**STRONG**	**MODERATE**	**WEAK**	
		1	2	3	
**E**	**DATA COLLECTION METHOD**	**STRONG**	**MODERATE**	**WEAK**	
		1	2	3	
**F**	**WITHDRAWALS AND DROPOUTS**	**STRONG**	**MODERATE**	**WEAK**	
		1	2	3	Not Applicable

<u>GLOBAL RATING FOR THIS PAPER (circle one):</u>

1	STRONG	(no WEAK ratings)
2	MODERATE	(one WEAK rating)
3	WEAK	(two or more WEAK ratings)

With both reviewers discussing the ratings:

Is there a discrepancy between the two reviewers with respect to the component (A-F) ratings?

No Yes

If yes, indicate the reason for the discrepancy

1 Oversight
2 Differences in interpretation of criteria
3 Differences in interpretation of study

<u>Final decision of both reviewers (circle one)</u>:

1 STRONG
2 MODERATE
3 WEAK

For peer review only - http://bmjopen.bmj.com/site/about/guidelines.xhtml

Supplementary file 5

Data extraction sheet

	Authors	Author affiliations	Funding for the study	Year of publication	Article type	Study design (for research papers)	Study setting	Country
Record 1								
Record 2								
Record 3								
….								

For peer review only
